# Global trends in incidence and death of neonatal disorders and its specific causes in 204 countries/territories during 1990–2019

**DOI:** 10.1186/s12889-022-12765-1

**Published:** 2022-02-19

**Authors:** Zejin Ou, Danfeng Yu, Yuanhao Liang, Huan He, Wenqiao He, Yongzhi Li, Minyi Zhang, Yuhan Gao, Fei Wu, Qing Chen

**Affiliations:** 1 Department of Central Laboratory, Guangzhou Twelfth People’s Hospital, Guangzhou, China; 2 Department of MICU, Guangdong Women and Children Hospital, Guangzhou, China; 3 Guangdong Provincial Key Laboratory of Tropical Disease Research, Department of Epidemiology, School of Public Health, Southern Medical University, 1838 Guangzhou North Road, Guangzhou, 510515 China

**Keywords:** Neonatal disorder, Age-standardized rate, Estimated annual percentage changes, Global burden of disease, Epidemiological trend

## Abstract

**Background:**

Neonatal disorders (ND) are a significant global health issue. This article aimed to track the global trends of neonatal disorders in 204 countries/territories from 1990 to 2019.

**Methods:**

Data was explored from the Global Burden of Disease study 2019. Estimated annual percentage change (EAPC) and age-standardized rate (ASR) were calculated to quantify the trends of neonatal disorders and their specific causes, mainly included neonatal preterm birth (NPB), neonatal encephalopathy due to birth asphyxia and trauma (NE), neonatal sepsis and other neonatal infections (NS), and hemolytic disease and other neonatal jaundice (HD).

**Results:**

In 2019, there were 23,532.23 × 10^3^ incident cases of ND, and caused 1882.44 × 10^3^ death worldwide. During 1990–2019, trends in the overall age-standardized incidence rate (ASIR) of ND was relatively stable, but that of age-standardized death rate (ASDR) declined (EAPC = -1.51, 95% confidence interval [CI]: -1.66 to -1.36). Meanwhile, decreasing trends of ASDR were observed in most regions and countries, particularly Cook Islands and Estonia, in which the respective EAPCs were -9.04 (95%CI: -9.69 to -8.38) and -8.12 (95%CI: -8.46 to -7.77). Among the specific four causes, only the NPB showed decreasing trends in the ASIR globally (EAPC = -0.19, 95%CI: -0.26 to -0.11). Decreasing trends of ASDR caused by ND underlying specific causes were observed in most regions, particularly the HD in Armenia, with the EAPC was -13.08 (95%CI: -14.04 to -12.11).

**Conclusions:**

Decreasing trends of death caused by neonatal disorders were observed worldwide from 1990 to 2019. However, the burden of neonatal disorders is still a considerable challenge, especially in low-resource settings, which need more effective health strategies.

**Supplementary Information:**

The online version contains supplementary material available at 10.1186/s12889-022-12765-1.

## Introduction

Neonatal health is a critical global concern, reflecting the national and global progress and challenges of health systems. Over the past decades, significant progress had made in improving neonatal survival [1, 2], with the death decreased by 42.4% since1990, and caused 2.6 million deaths in 2015 [3]. Neonatal disorders ranked the second leading cause of YLLs (accounted for 1.78 million) in 2017, with a decrease of 24.1% since 2007 [4]. Dramatical progress in neonatal diseases was mainly due to socioeconomic factors, including economic growth, international collaboration, improved health systems and service [5–7].

However, the considerable disequilibrium of neonatal disorders was found across regions and countries, deeply dependent on the local demographics and socioeconomic status [2, 8, 9]. Neonatal diseases are still the leading public problems in low-resource settings, e.g., serious infections [10], neonatal encephalopathy, and neonatal preterm birth complications [11, 12]. In sub-Saharan Africa, neonatal sepsis caused an estimated 5.3–8.7 million disability-adjusted life-years (DALYs), and an economic burden of over 469 billion US dollars in 2014 [13]. The challenges of maternal malnutrition, poor living conditions, and health resources were still substantial [14–16]. Neonatal disorders were a critical part of the UN’s Sustainable Development Goals (SDG) [17], and their epidemiological patterns were essential to health decisions.

The Global Burden of Disease study (GBDs) comprehensively assessed and quantified the burden of diseases and causes globally, which provided an opportunity to track the changing trends of neonatal disorders. Therefore, this article aimed to estimate the trends of neonatal disorders and underlying specific causes from 1990 to 2019.

## Methods

### Study data

The GBD 2019 comprehensively assessed 369 diseases and injuries and risk factors for 204 countries/territories [18]. The GBD collaborators collect related data sources from censuses, civil registration, and vital statistics, and so on. The data is estimated using a Bayesian meta-regression tool named DisMod-MR model, which is detailly described in previous articles [3, 19]. Data on the incidence and death of neonatal disorders was explored through the Global Health Data Exchange query tool (http://ghdx.healthdata.org/gbd-results-tool). According to the GBD online tools instruction, data was collated by specific causes, and multiple geographic dimensions from 1990 to 2019, without any inclusion/exclusion criteria. The specific causes of neonatal disorders mainly included (1) neonatal preterm birth (NPB), (2) neonatal encephalopathy due to birth asphyxia and trauma (NE), (3) neonatal sepsis and other neonatal infections (NS), and (4) hemolytic disease and other neonatal jaundice (HD). The GBD study provided a comprehensive assessment of neonatal disorders worldwide, including 21 geographic regions (e.g., South Asia, Caribbean, and Central Europe), and 204 countries/territories (e.g. United Kingdom, China, and Greece). By the socio-demographic index (SDI), regions and countries were categorized into 5 levels, including low, low-middle, middle, high-middle, and high.

### Statistical analysis

Age-standardization is a necessary and representative step when data involved in multiple population with different age structures or for the same population over time. The age-standardized rate (ASR)/100,000 population was estimated with the following formula:

$$\mathrm{ASR }= \frac{\sum \genfrac{}{}{0pt}{}{A}{i=1}{a}_{i}{w}_{i}}{\sum \genfrac{}{}{0pt}{}{A}{i=1}{w}_{i}}\times 10\mathrm{0,000}$$

In the above formula, *a*_*i*_ meant the age-specific rate in the *i*^*th*^ age group, *w* meant the number of people (or the weight) in the corresponding *i*^*th*^ age group from among the selected reference standard population, and *A* meant the number of age groups.

Estimated annual percentage change (EAPC) is a well-accepted method of quantifying the trends of ASR, which have been wildly used in public health studies [20, 21]. A regression line was fitted to the natural logarithm of the rates. The EAPC and its 95% confidence interval (CI) were estimated using the linear regression model. The formulas were as following:

$${\varvec{y}}={\varvec{a}}+{\varvec{\beta}}{\varvec{x}}+{\varvec{\varepsilon}}$$

$$\mathbf{E}\mathbf{A}\mathbf{P}\mathbf{C}=100\times (\mathbf{e}\mathbf{x}\mathbf{p}\left({\varvec{\upbeta}}\right)-1)$$

Thereinto, *y* = ln (ASR) and *x* = calendar year. The trends were determined as follows: 1) An increasing trend was determined if both EAPC and 95% CI were > 0; 2) a decreasing trend was determined if both EAPC and 95% CI were < 0; 3) Others were deemed to be “stable” over time. In order to explore the influential factors of EAPC, the associations between EAPCs and ASDR in 1990, and between EAPCs and HDI in 2019 were calculated using Pearson correlation analysis. Data were analyzed using R 3.6.2 (Lucent Technologies, Jasmine Mountain, USA). A *p* value of less than 0.05 was considered to be statistically significant.

## Results

### Trends in incidence and death of neonatal disorders

**Fig. 1:**
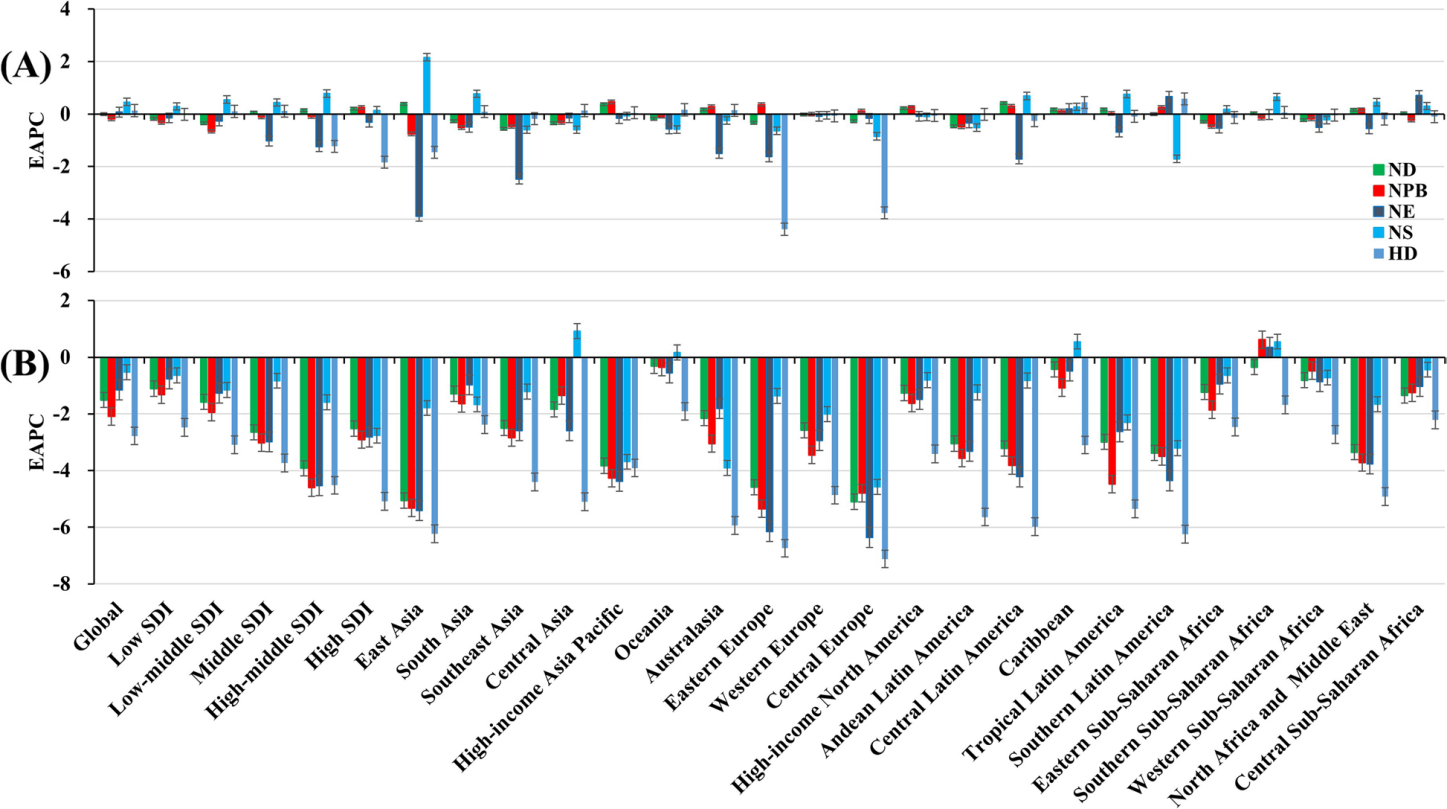
Trends in ASR of neonatal disorders globally, and in SDI areas and geographic regions, 1990–2019. **A** and (**B**) were that of incidence and death of neonatal disorders, respectively. ASR, age-standardized rate; EAPC, Estimated annual percentage change; SDI, sociodemographic index

**Fig. 2:**
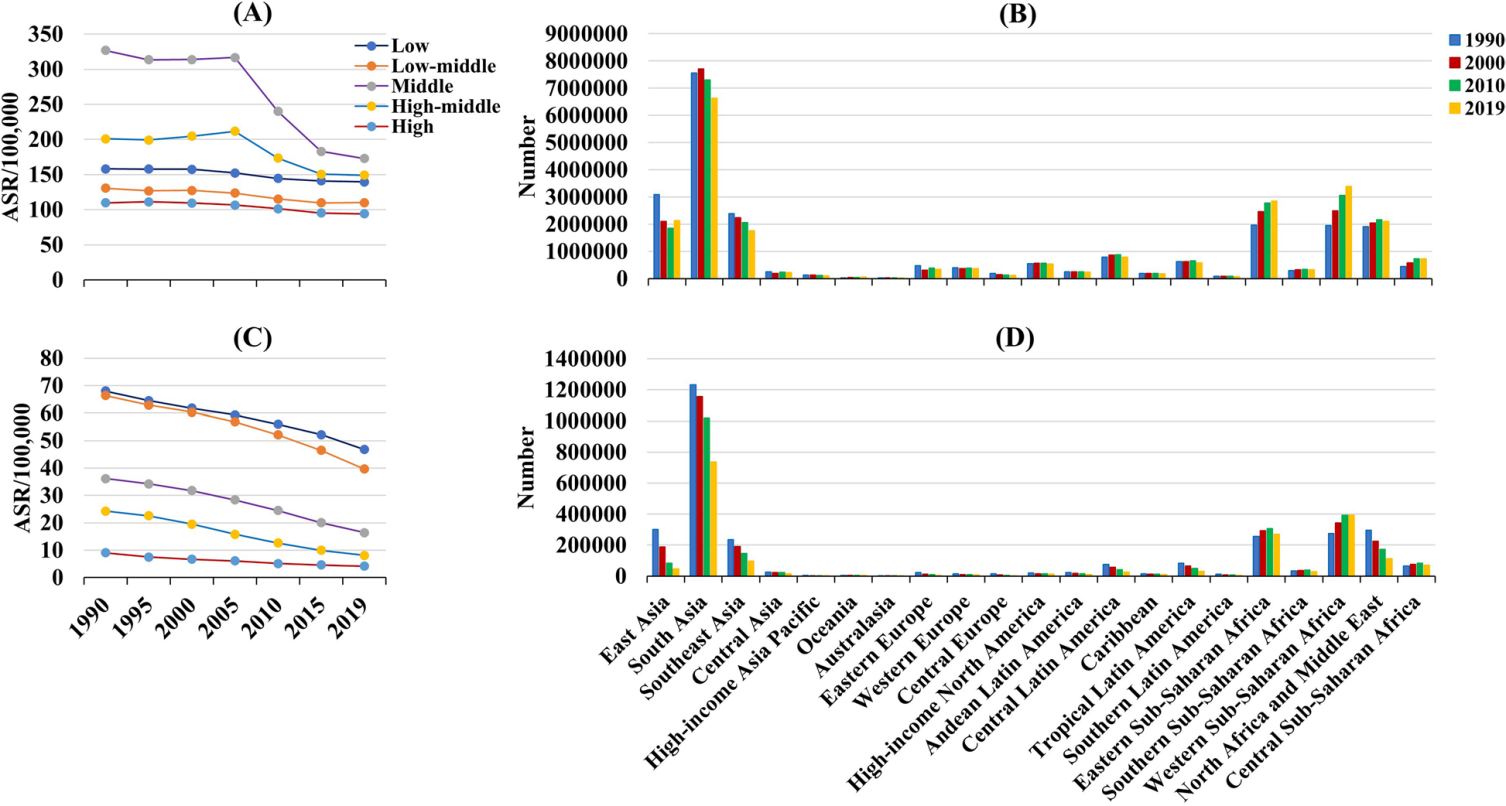
The distribution of incidence and death of neonatal disorders in SDI areas and regions. **A** was the ASR of incidnece in SDI areas from 1990 to 2019; **B** was the incident number in geographical regions; **C** was the ASR of death in SDI areas; **D** was the death number in geographical regions. ASR, age-standardized rate SDI, sociodemographic index

**Fig. 3:**
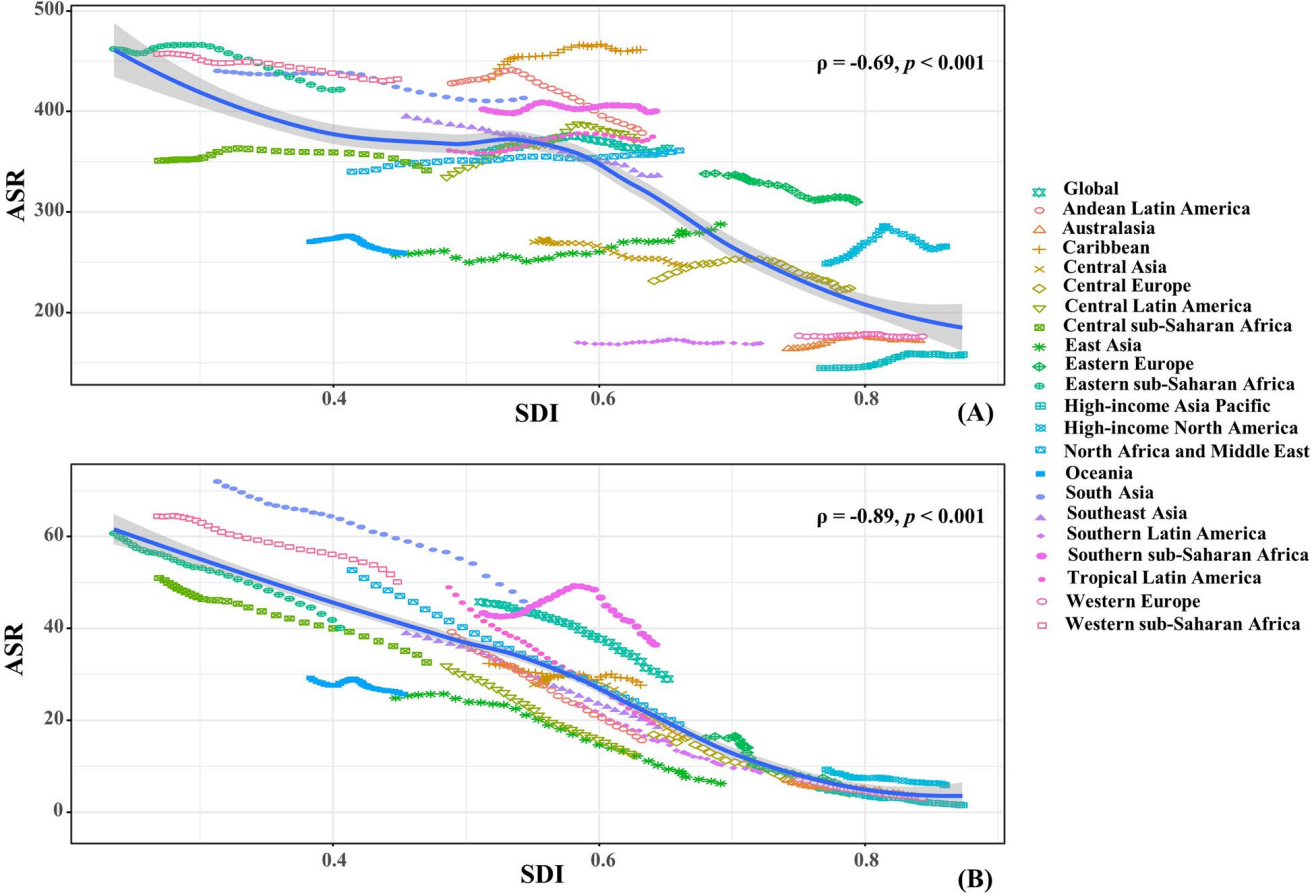
The associations between ASRs of neonatal disorders and SDI among regions. **A** and (**B**) were that of incidence and death, respectively. ASRs were that of from 1990 to 2019, and the abscissa was SDI in 2019. The associations were calculated with Pearson correlation analysis. The symbols were the countries/territories in the corresponding regions. ASR, age-standardized rate; SDI, socio-demographic index

**Fig. 4:**
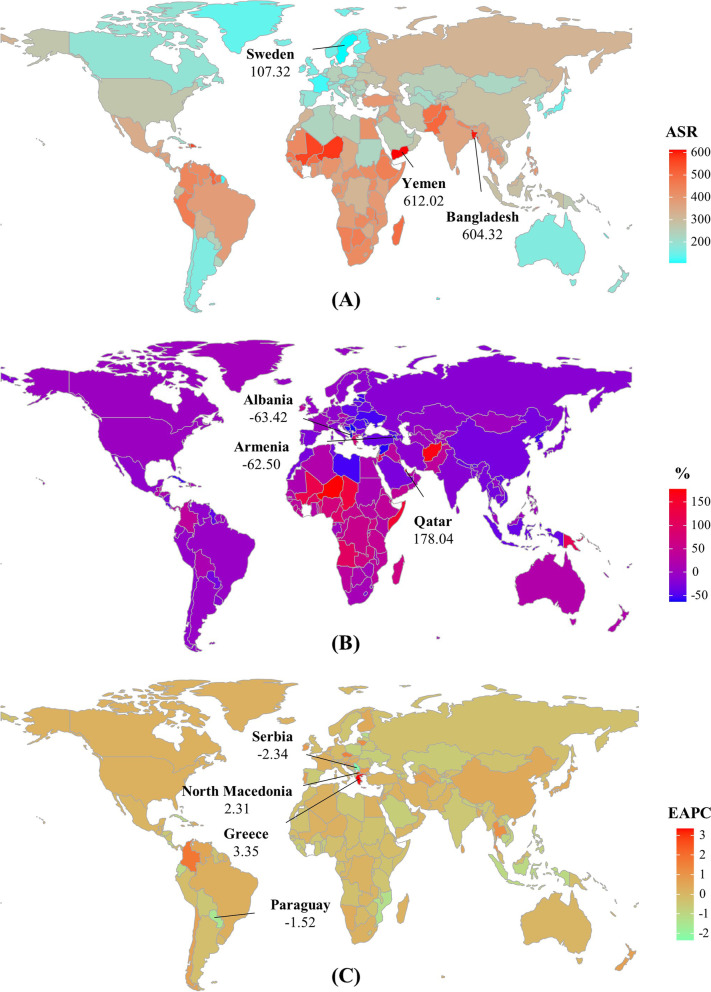
The distribution of ASR, percentage changes, and EAPCs of neonatal disorders incidence, 1990–2019. Countries/territories with an extreme value were annotated. ASR, age-standardized rate; EAPC, estimated annual percentage change

The incident number of neonatal disorders was 23,532.23 × 10^3^ (95% uncertainty interval (UI): 21,672.53 × 10^3^–25,701.96 × 10^3^) worldwide in 2019, with a decrease of 0.26% since 1990. The overall trend in age-standardized incidence rate (ASIR) was relatively stable during 1990–2019. The decreasing incident trends were observed in low and low-middle SDI areas, particularly the letter (EAPC = -0.35, 95% CI: -0.41 to -0.29). Whereas increasing trends occurred in the high SDI areas, with the EAPC was 0.21 (95%CI: 0.11–0.31). At the regional level, decreasing trends were observed in nine regions, particularly Southeast Asia (EAPC = -0.57, 95%CI: -0.60 to -0.54). Conversely, increasing trends were seen in eight regions, and the most pronounced one occurred in Central Latin America (EAPC = 0.42, 95%CI: 0.33–0.50), followed by High-income Asia Pacific and East Asia (Table 1; Figs. 1A and 2A-B). A negative correlation was found between ASIRs and SDI in 2019 among regions (*ρ* = -0.69, *p* < 0.001; Fig. 3A). The ASIR of neonatal disorders in 2019 was heterogeneous among 204 countries/territories, ranging from 107.32/100,000 in Sweden to 612.02/100,000 in Yemen. 1990–2019, the percentages of incident numbers changed from -63.42% in Albania to 178.04% in Qatar. Decreasing trends of ASIR were observed in 115 countries/territories, particularly in Serbia and Paraguay, in which the respective EAPCs were -2.34 (95%CI: -2.58 to -2.10) and -1.52 (95%CI: -1.62 to -1.43). Whereas increasing trends were seen in 77 countries/territories, and the largest ones were Greece (EAPC = 3.35, 95% CI: 3.13–3.56), followed by North Macedonia, and Colombia (Supplementary table 1; Fig. 4A-C).

**Table 1:** The characteristics and trend of incidence and death of neonatal disorders in global, SDI areas and geographic regions from 1990 to 2019

**Characteristics**	Incidence				Death			
	2019		1990–2019		2019		1990–2019	
	Number × 10^3^ (95% UI)	ASR/100,000 (95% UI)	Percentage (%)	EAPC (95%CI)	Number × 10^3^ (95% UI)	ASR/100,000 (95% UI)	Percentage (%)	EAPC (95%CI)
**Overall**	23,532.23 (21,672.53–25,701.96)	363.34 (334.63–396.84)	-0.26	0 (-0.06–0.06)	1882.44 (1605.83–2237.48)	29.05 (24.78–34.53)	-37.38	-1.51 (-1.66–-1.36)
**SDI**								
Low	7391.71 (6920.05–7964.27)	409.53 (383.39–441.23)	41.52	-0.19 (-0.25–-0.13)	843.27 (697.74–1027.87)	46.79 (38.71–57.02)	10.27	-1.11 (-1.19–-1.04)
Low-middle	6495.69 (6006.89–7056.88)	382.44 (353.64–415.47)	-15.05	-0.35 (-0.41–-0.29)	674.01 (572.36–788.39)	39.69 (33.7–46.42)	-43.82	-1.58 (-1.73–-1.43)
Middle	5480.71 (4906.17–6154.04)	319.16 (285.75–358.23)	-18.13	0.05 (-0.01–0.10)	281.95 (239.05–332.82)	16.39 (13.89–19.34)	-62.24	-2.65 (-2.88–-2.42)
High-middle	2236.56 (1987.56–2519.84)	295.87 (263–333.23)	-21.17	0.14 (0.12–0.17)	61.33 (52.38–71.3)	8.07 (6.90–9.39)	-74.64	-3.92 (-4.11–-3.72)
High	1089.95 (1054.99–1127.07)	219.55 (212.54–226.99)	-7.44	0.21 (0.11–0.31)	20.81 (18.55–23.25)	4.18 (3.72–4.67)	-59.65	-2.52 (-2.6–-2.44)
**Regions**								
East Asia	2136.20 (1771.25–2569.02)	287.93 (238.83–345.89)	-31.01	0.38 (0.29–0.48)	47.14 (40.28–54.58)	6.30 (5.39–7.30)	-84.29	-5.06 (-5.52–-4.59)
South Asia	6629.24 (6153.26–7174.37)	413.47 (383.73–447.43)	-12.31	-0.27 (-0.32–-0.22)	736.60 (625.57–870.78)	45.96 (39.03–54.33)	-40.23	-1.29 (-1.43–-1.15)
Southeast Asia	1757.89 (1535.71–2004.84)	335.96 (293.54–383.19)	-26.21	-0.57 (-0.60–-0.54)	96.72 (78.67–118.15)	18.46 (15.02–22.56)	-58.78	-2.50 (-2.61–-2.38)
Central Asia	223.78 (207.89–240.25)	247.21 (229.69–265.34)	-11.47	-0.36 (-0.41–-0.31)	14.81 (12.33–17.99)	16.33 (13.59–19.84)	-43.23	-1.84 (-2.29–-1.38)
High-income Asia Pacific	104.93 (101.37–108.62)	158.44 (153.06–163.99)	-23.45	0.37 (0.29–0.44)	1.06 (0.93–1.20)	1.59 (1.39–1.79)	-78.56	-3.83 (-3.99–-3.66)
Oceania	51.01 (47.73–54.57)	259.08 (242.45–277.19)	77.34	-0.19 (-0.25–-0.14)	5.06 (3.67–6.80)	25.73 (18.71–34.62)	63.27	-0.31 (-0.41–-0.22)
Australasia	30.46 (28.79–32.06)	172.42 (162.98–181.51)	21.85	0.18 (0.09–0.26)	0.59 (0.49–0.70)	3.32 (2.74–3.97)	-45.17	-2.15 (-2.33–-1.98)
Eastern Europe	336.35 (291.03–391.20)	310.06 (268.39–360.67)	-29.25	-0.34 (-0.38–-0.30)	5.23 (4.21–6.38)	4.79 (3.86–5.85)	-77.05	-4.59 (-4.85–-4.33)
Western Europe	365.37 (355.65–375.56)	176.52 (171.82–181.43)	-7.46	-0.02 (-0.04–0)	6.47 (5.40–7.64)	3.11 (2.60–3.67)	-59.80	-2.58 (-2.72–-2.44)
Central Europe	115.81 (108.22–124.70)	224.33 (209.70–241.47)	-37.77	-0.28 (-0.42–-0.13)	2.13 (1.67–2.65)	4.11 (3.23–5.12)	-84.42	-5.10 (-5.28–-4.93)
High-income North America	536.71 (520.57–555.11)	266.04 (258.06–275.15)	-1.94	0.22 (0.06–0.37)	11.91 (10.85–13.10)	5.89 (5.36–6.48)	-42.12	-1.26 (-1.38–-1.13)
Andean Latin America	238.24 (215.06–259.15)	378.77 (341.91–412.02)	-3.65	-0.48 (-0.58–-0.38)	9.91 (7.41–12.85)	15.72 (11.76–20.39)	-56.27	-3.05 (-3.21–-2.89)
Central Latin America	785.71 (699.02–879.68)	372.29 (331.29–416.66)	-1.12	0.42 (0.33–0.50)	26.14 (20.14–32.73)	12.36 (9.52–15.47)	-65.44	-3.22 (-3.30–-3.13)
Caribbean	180.21 (169.85–190.65)	461.44 (434.91–488.20)	-3.83	0.17 (0.12–0.22)	10.83 (8.18–13.92)	27.72 (20.93–35.63)	-22.97	-0.43 (-0.50–-0.36)
Tropical Latin America	579.79 (523.43–652.13)	375.11 (338.70–421.77)	-6.29	0.18 (0.14–0.23)	30.21 (23.87–37.33)	19.47 (15.39–24.06)	-63.96	-2.99 (-3.15–-2.82)
Southern Latin America	78.60 (74.43–83.46)	169.46 (160.45–179.91)	-7.93	0 (-0.04–0.03)	4.01 (3.08–5.09)	8.63 (6.62–10.95)	-65.95	-3.38 (-3.46–-3.30)
Eastern Sub-Saharan Africa	2846.01 (2601.34–3150.78)	422.06 (385.74–467.23)	44.70	-0.30 (-0.39–-0.22)	270.67 (215.65–340.55)	40.19 (32.01–50.55)	5.40	-1.23 (-1.34–-1.12)
Southern Sub-Saharan Africa	318.92 (296.48–348.02)	400.68 (372.46–437.24)	7.60	0.02 (-0.01–0.05)	29.05 (22.64–37.35)	36.49 (28.43–46.91)	-9.08	-0.35 (-0.67–-0.03)
Western Sub-Saharan Africa	3387.57 (3156.76–3678.13)	432.39 (402.89–469.46)	73.32	-0.23 (-0.25–-0.21)	392.48 (328.33–476.7)	50.17 (41.98–60.94)	42.95	-0.81 (-0.87–-0.75)
North Africa and Middle East	2102.53 (2016.48–2198.11)	361.32 (346.55–377.75)	10.21	0.16 (0.13–0.18)	111.94 (93.40–134.35)	19.19 (16.02–23.04)	-61.99	-3.35 (-3.44–-3.26)
Central Sub-Saharan Africa	726.89 (672.38–791.57)	341.45 (315.84–371.84)	62.23	0.02 (-0.04–0.08)	69.48 (57.97–83.64)	32.65 (27.24–39.31)	7.22	-1.35 (-1.50–-1.21)

*EAPC* estimated annual percentage change, *ASR* age-standardized rate, *CI* confidence interval, *UI* uncertainty interval, *SDI*, socio-demographic index

**Fig. 5:**
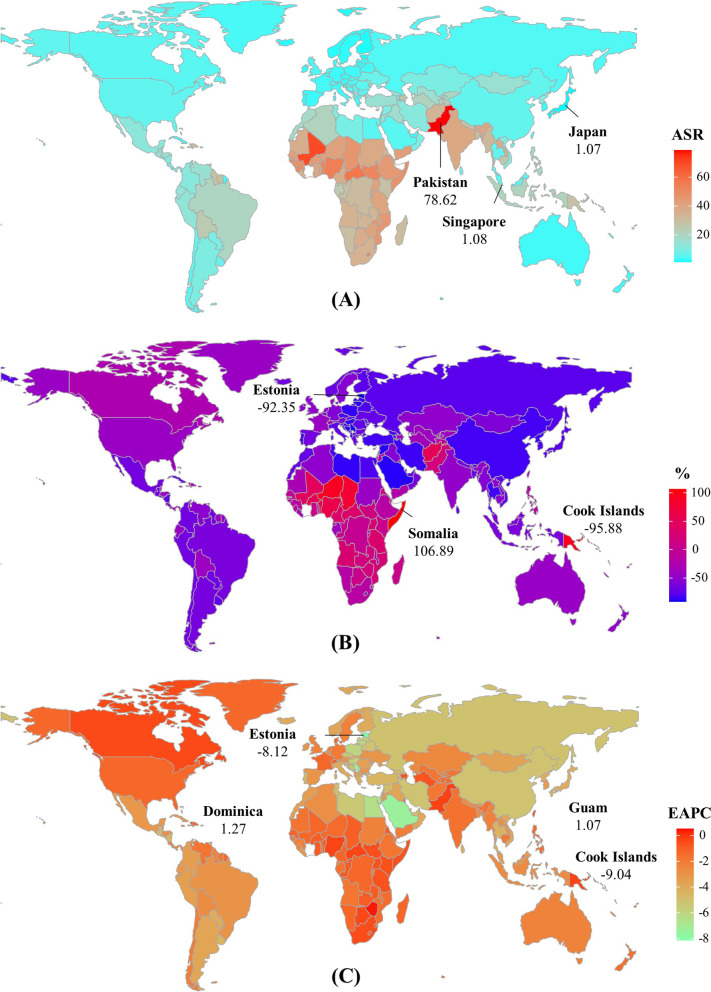
The distribution of ASR, percent changes, and EAPCs of death caused by neonatal disorders, 1990–2019. Countries/territories with an extreme value were annotated. ASR, age-standardized rate; EAPC, estimated annual percentage change

Globally, the death number of neonatal disorders decreased 37.38% between 1990–2019, and it was 1882.44 × 10^3^ (95% UI: 1605.83 × 10^3^–2237.48 × 10^3^) in 2019. The age-standardized death rate (ASDR) decreased by an average 1.51% per year in 1990–2019 (EAPC = -1.51, 95%CI: -1.66 to -1.36). Decreasing trends of neonatal disorders were observed in all SDI areas and geographic regions, particularly Central Europe and East Asia, and their respective EAPCs were -5.10 (95%CI: -5.28 to -4.93) and -5.06 (95%CI: -5.52 to -4.59) (Table 1; Figs. 1B and 2C-D). A negative correlation was found between ASDRs and SDI in 2019 among regions (*ρ* = -0.89, *p* < 0.001; Fig. 3B). The ASDR of neonatal disorders in 2019 was heterogeneous among 204 countries/territories, ranging from 1.07/100,000 in Japan to 78.62/100,000 in Pakistan. In the past three decades, the percentage changes in number varied from -95.88% in the Cook Islands to 106.89% in Somalia. The trends of ASDR declined in 197 countries/territories, and the Cook Islands and Estonia had the most pronounced ones, with the respective EAPCs were -9.04 (95%CI: -9.69 to -8.38) and -8.12 (95%CI: -8.46 to -7.77). Conversely, increasing trends were seen in four countries, particularly Dominica (EAPC = 1.27, 95%CI: 1.02–1.53) (Supplementary table 1; Fig. 5A-C).

### Trends in incidence and death of neonatal preterm birth (NPB)

During 1990–2019, the overall ASIR of NPB showed a decreasing trend (EAPC = -0.19, 95%CI: -0.26 to -0.11). Meanwhile, decreasing trends were observed in most SDI areas, and the most pronounced one was in the low-middle SDI area (EAPC = -0.66, 95%CI: -0.75 to -0.58). In contrast, only increasing one occurred in high SDI area (EAPC = 0.25, 95%CI: 0.13–0.38). At the regional level, decreasing trends were seen in nine geographic regions, particularly East Asia (EAPC = -0.76, 95%CI: -0.89 to -0.63). On the other hand, increasing trends were seen in nine regions, especially High-income Asia Pacific (EAPC = 0.46, 95%CI: 0.37–0.56) (Supplementary table 2; Fig. 1A, and Supplementary Fig. 1A-B). In 1990–2019, the percentage changes among 204 countries/territories ranged from -67.71% in Albania to 182.10% in Niger. Decreasing trends of ASIR were demonstrated in 104 countries/territories, and the largest ones were seen in Mozambique (EAPC = -1.82, 95%CI: -2.04 to -1.59), followed by Cambodia and Cuba. On the contrary, increasing trends were observed in eighty countries/territories, particularly Greece (EAPC = 3.91, 95%CI: 3.66–4.17) (Supplementary table 3; Supplementary Figs. 5A and 6A).

Globally, decreasing trends in the ASDR of NPB were observed worldwide in 1990–2019, with the EAPC of -2.09 (95%CI: -2.19 to -2.00). Meanwhile, decreasing trends also were seen in SDI areas and most regions, and the most pronounced ones were Eastern Europe and East Asia, and the respective EAPCs were -5.35 (95%CI: -5.69 to -5.02) and -5.32 (95%CI: -5.59 to -5.06) (Supplementary table 4; Fig. 1B, and Supplementary Fig. 1C-D). At the national level, the highest increasing percentage in death number of NPB was observed in Niger (105.52%) between 1990 and 2019. Whereas the largest decreasing one occurred in the Cook Islands (-97.15%). Decreasing trends in the ASDR of NPB were demonstrated in 188 countries/territories, and the largest one was seen in Cook Islands (EAPC = -10.23, 95%CI: -10.98 to -9.48), followed by Saudi Arabia and Czechia. Conversely, increasing trends were seen in 7 countries, especially Guam (EAPC = 1.93, 95%CI: 1.47–2.39) (Supplementary table 5; Supplementary Figs. 7A and 8A).

### Trends in incidence and death of neonatal encephalopathy due to birth asphyxia and trauma (NE)

The overall ASIR of NE was relatively stable during 1990–2019. However, decreasing trends of ASIR were observed in most SDI areas and regions, particularly East Asia (EAPC = -3.90, 95%CI: -4.20 to -3.60) (Supplementary table 2; Fig. 1A, and Supplementary Fig. 2A-B). During 1990–2019, the percentage changes of NE were observed from -76.84% in China to 183.47% in Qatar. Decreasing trends were seen in 133 countries/territories, and the largest ones occurred in China (EAPC = -3.96, 95%CI: -4.27 to -3.66), followed by Indonesia and Nicaragua. Whereas increasing trends occurred in fifty-two ones, particularly Dominica (EAPC = 2.40, 95%CI: 2.34–2.47) (Supplementary table 3; Supplementary Figs. 5B and 6B).

Decreasing trend in the ASDR of NE was observed worldwide in 1990–2019 (EAPC = -1.16, 95%CI: -1.38 to -0.94). Meanwhile, decreasing trends were observed in SDI areas and most regions, particularly Central Europe (EAPC = -6.36, 95%CI: -6.56 to -6.15) (Supplementary table 4; Fig. 1B, and Supplementary Fig. 2C-D). Over the past three decades, the percentage changes in the death number of NE varied from -96.49% in Cook Islands to 139.53% in Somalia. Decreasing trends of ASDR were observed in 191 countries/territories, especially the Cook Islands and Hungary, with the EAPCs of -9.60 (95%CI: -10.29 to -8.90) and -9.44 (95%CI: -10.99 to -7.87). However, increasing trends were found in six countries, and the most pronounced one occurred in Dominica (EAPC = 2.05, 95%CI: 1.64–2.46) (Supplementary table 5; Supplementary Figs. 7B and 8B).

### Trends in incidence and death of neonatal sepsis and other neonatal infections (NS)

The global trend in the ASIR of NS increased from 1990 to 2019, with the EAPC of 0.46 (95%CI: 0.43–0.48). Meanwhile, increasing trends were seen in SDI areas and most regions, particularly East Asia (EAPC = 2.16, 95%CI: 2.04–2.27). Whereas decreasing trends occurred in ten regions, and the most pronounced ones were in Southern Latin America (EAPCs = -1.71, 95%CI: -1.85 to -1.57) (Supplementary table 2; Fig. 1A, and Supplementary Fig. 3A-B). At the national level, the percentages in the incident number of NS changed from -78.64% in Serbia to 300.45% in North Macedonia during 1990–2019. Decreasing trends in the ASIR were demonstrated in 86 countries/territories, particularly Serbia (EAPC = -4.76, 95%CI: -5.26 to -4.26). On the other hand, increasing trends were seen in 101 countries/territories, and the most pronounced one was North Macedonia (EAPC = 8.41, 95%CI: 7.72–9.10), followed by Bulgaria and Lithuania (Supplementary table 3; Supplementary Figs. 5C and 6C).

The ASDR of NS showed a decreasing trend globally from 1990 to 2019 (EAPC = -0.53, 95%CI: -0.72 to -0.35). Decreasing trends of ASDR were observed in all SDI areas, especially the high SDI area (EAPC = -2.77, 95%CI: -2.98 to -2.55). Decreasing trends were seen in seventeen regions, and the largest ones were seen in Central Europe (EAPC = -4.58, 95%CI: -5.03 to -4.13). However, increasing trends were observed in three regions, including Central Asia, Caribbean, and Southern sub-Saharan Africa (Supplementary table 4; Fig. 1B, and Supplementary Fig. 3C-D). In 1990–2019, the percentages in NS number altered -93.00% in Greece from 227.49% in North Macedonia. Increasing trends were observed in thirty-eight countries/territories, and the largest one was seen in North Macedonia (EAPC = 9.31, 95%CI: 7.81–10.83), followed by Bulgaria and Taiwan. Conversely, decreasing trends were seen in 156 countries/territories, particularly Serbia and Greece, with the respective EAPCs were -8.66 (95%CI: -9.83 to -7.47) and -7.41 (95%CI: -9.32 to -5.46) (Supplementary table 5; Supplementary Figs. 7C and 8C).

### Trends in incidence and death of hemolytic disease and other neonatal jaundice (HD)

The ASIR of HD showed an increasing trend over the past 30 years, with the EAPC of 0.13 (95%CI: 0.03–0.23). Meanwhile, increasing trends were also seen in low-middle and middle SDI areas. Whereas decreasing trends occurred in high-middle and high SDI areas, in which the respective EAPCs were -1.23 (95%CI: -1.57 to -0.90) and -1.84 (95%CI: -2.30 to -1.38). Among 21 regions, the most pronounced decreasing trends were observed in Eastern Europe (EAPC = -4.39, 95%CI: -5.17 to -3.61) (Supplementary table 2; Fig. 1A, and Supplementary Fig. 4A-B). During 1990–2019, the percentages altered from -98.22% in Libya to 179.84% in Afghanistan. There were 109 countries/territories had decreasing trends in the ASIR, and the most pronounced one was seen in Latvia (EAPC = -14.43, 95%CI: -16.64 to -12.16), followed by Montenegro and Libya. On the other hand, there were sixty ones had increasing trends, particularly the United Kingdom and Georgia, with the respective EAPCs were 0.71 (95%CI: 0.52–0.90) and 0.69 (95%CI: 0.64–0.75) (Supplementary table 3; Supplementary Figs. 5D and 6D).

Compared with other causes of neonatal disorders, HD had a most pronounced decreasing trend in the ASDR globally from 1990 to 2019 (EAPC = -2.78, 95%CI: -3.00 to -2.57). Meanwhile, decreasing trends of ASDR were observed in all SDI areas and regions, especially Central Europe (EAPC = -7.12, 95%CI: -7.37 to -6.86) (Supplementary table 4; Fig. 1B, and Supplementary Fig. 4C-D). In 1990–2019, the percentages in death number altered from -98.46% in Armenia to 64.24% in Somalia. Decreasing trends in the ASDR were observed in 200 countries/territories, and the largest one was seen in Armenia (EAPC = -13.08, 95%CI: -14.04 to -12.11), followed by the Cook Islands and Nicaragua. Furthermore, the trends were stable in the rest four countries/territories over time, including Azerbaijan, Guatemala, Zimbabwe, and Sri Lanka (Supplementary table 5; Supplementary Figs. 7D and 8D).

### Analysis on the influential factors of EAPCs

**Fig. 6:**
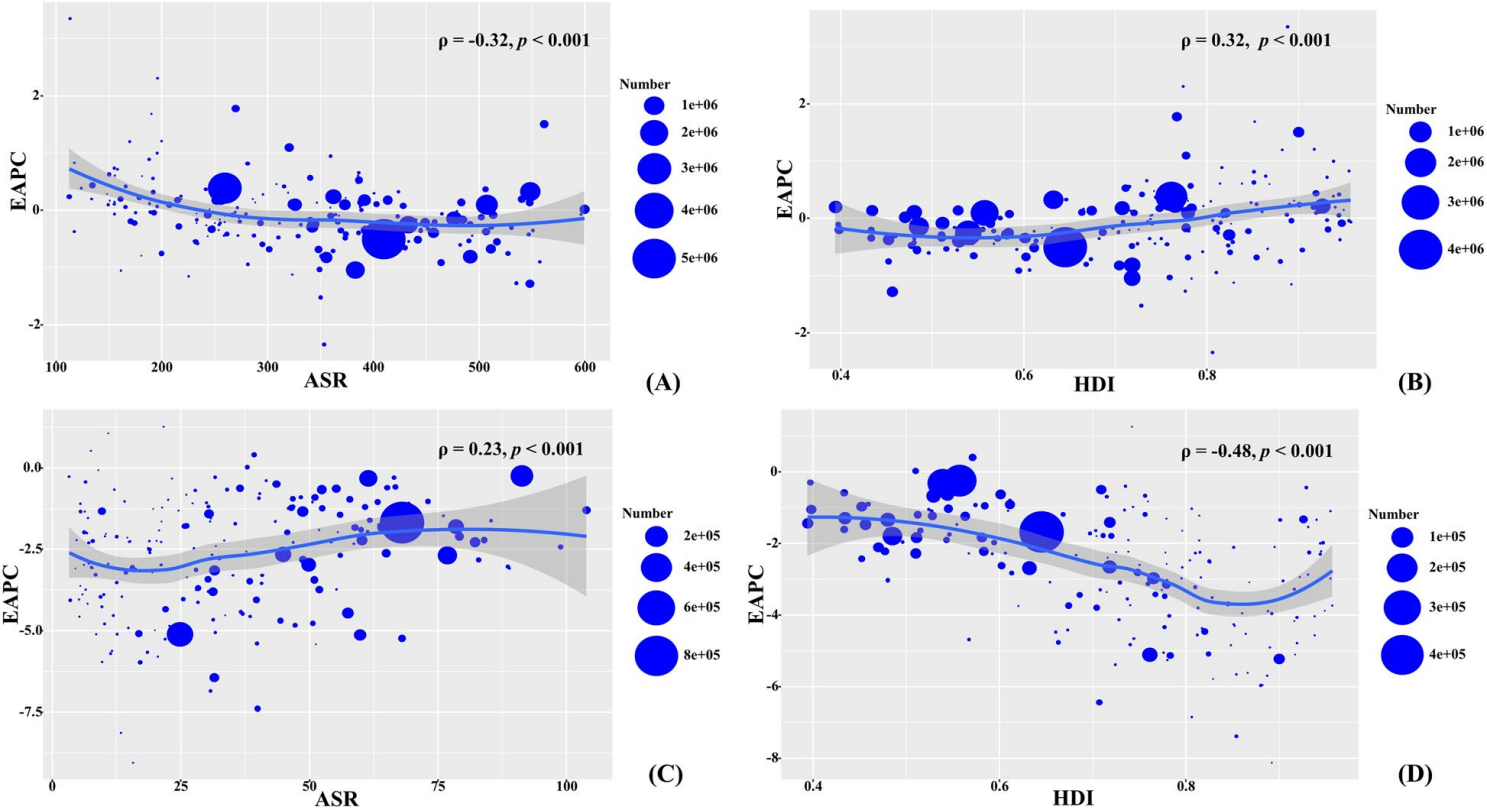
The association between EAPCs of neonatal disorders and ASR in 1990, and between EAPCs and HDI in 2019. **A** is the association between EAPCs and ASIR in 1990; **B** is the association between EAPCs of incidence and HDI in 2019; **C** is the association between EAPCs and ASDR in 1990; **D** is the association between EAPCs of death and HDI in 2019. The associations were calculated with Pearson correlation analysis. The size of circle is increased with the numbers in 1990 and 2019, respectively. EAPC, estimated annual percentage change; ASIR, age-standardized incidence rate; ASDR, age-standardized death rate

The ASR in 1990 means the disease reservoir at baseline, and the HDI is a critical index reflecting the level of human development and health resources in regions and countries. At the national level, EAPCs had a negative association with the ASIR in 1990 (*ρ* = -0.32, *p* < 0.001, Fig. 6A), and a positive association with the HDI in 2019 (*ρ* = 0.32, *p* < 0.001; Fig. 6B). Meanwhile, in terms of death caused by neonatal disorders, EAPCs had a positive association with the ASDR in 1990 (*ρ* = 0.23, *p* < 0.001, Fig. 6C), and a negative association with HDI in 2019 (*ρ* = -0.48, *p* < 0.001, Fig. 6D).

## Discussion

Neonatal health is an important public issue worldwide, which attracted huge funding, domestic policies, and international corporations in recent decades [22–24]. EAPC and ASR were used to demonstrate the changing trends of ND, facilitating the horizontally comparison among countries over a long-term interval. The results would be helpful to identify progress and obstacles in disease control, which informed the adjustment and formulation of health strategies. In the present article, the incident trends of neonatal disorders were relatively stable globally from 1990 to 2019, probably being impacted by the population growth, imbalanced allocation of resources, weak health systems, and local socio-economic factors [25–27]. The results showed that the burden of ND was significantly associated with the social and economic development status. Low and middle SDI settings undertook the heaviest burden, in where there faced rapid population growth, maternal malnutrition, high prevalence infections, lack of prenatal screening, and limited medical resources [28, 29]. The high morbidity and mortality caused a considerable economic cost, and also exacerbated the social welfare and resource [30], which became a vicious circle subsequently. Among specific causes, neonatal preterm birth had a decreasing incident trend probably related to the improved health systems [31, 32]. Whereas increasing trends were found in NS and HD from 1990 to 2019. The high prevalence of infectious diseases constantly threated the neonates in low- and middle-income countries [26], driving the increasing trends of NS. However, it was reported that the incidence and prevalence of alloimmune hemolytic disease had decreased over the past decades [33]. The difference between ours and the above studies were probably due to disease categories and data sources. Decreasing trends of neonatal disorders commonly occurred in the former Soviet Union countries, probably because of the reconstruction of economy and health systems in recent years, especially in Serbia [34, 35]. Meanwhile, primary healthcare and newborn screening effectively decreased the incidence of neonatal disorders in Paraguay [36, 37]. However, Greece had the largest increasing incident trends of neonatal disorders, in where the economic crisis in 2008 brought about considerable impacts on perinatal outcomes [38, 39]. Adolescent mothers were very common in north Macedonia, who had considerably higher frequencies of perinatal and neonatal disorders [40], and prenatal and maternity care were also influenced by racism and discrimination [41].

Decreasing trends of death caused by neonatal disorders were demonstrated worldwide, and in most regions and countries from 1990 to 2019, indicating that considerable progress in the decline of neonatal mortality during the past decades [42–44]. The reasons involved the improvement of mental health care, health infrastructure, and poverty elimination [45, 46]. However, the relatively slow trends occurred in low- and middle-SDI areas were also related to the high non-health government expenditure [47]. Meanwhile, substantial heterogeneity in trends were observed between regions and countries. The Pacific low- and middle-income countries had taken actions to improve to improve the quality of maternal and newborn care over the past decades, including improvements of health infrastructure, considerable financial investment, and training program of healthcare workers [48]. Several island countries launched the Early Essential Newborn Care program (EENC) introduced from the WHO [49]. Meanwhile, the international medical program [50], and the reproductive health study [51] probably promoted trends of neonatal disorders declined pronouncedly in Cook Islands. In Armenia, the reductions in infant mortality were due entirely to a decline in post-neonatal mortality [52]. Studies have linked the pronounced decreasing trends in China to rapid economic growth, rising maternal education, and health policies [5, 53]. In low-resource regions and countries still face increasing challenges of maternal and neonatal disorders for the health systems [54].

Several limitations in this study should be interpreted. First, the GBD estimates depended upon the quality and quantity of data, and the accuracy and reliability of the results were impacted by the potential bias due to misclassification and miscoding. Second, the diagnostic standards and measures of neonatal disorders and specific causes had refined over time, which also affected the estimation of trends. Last but not least, studies on neonatal disorders and specific causes were few in some countries, thus the causes of trends could not be fully discussed in those countries.

## Conclusions

Neonatal disorders were a critical issue of the UN’s SDG. This article demonstrated the progress and challenges in the control and management of neonatal disorders using the GBD data. The results showed that global trends of death caused by neonatal disorders and its specific causes declined in most regions and countries from 1990 to 2019. However, the overall trend in the incidence of neonatal disorders declined slowly. The burden of neonatal disorders remained a considerable challenge to the global public health, particularly in low-resource settings. These findings highlighted the progress and challenges in the control and management of neonatal disorders, which would facilitate the adjustment of health strategies to the UN’s SDG at the global, regional, and national levels.

## Supplementary Information

**Additional file 1: Figure S1.** The distribution of number and ASR of incidence and death of neonatal preterm birth in SDI areas, and geographic regions from1990 to 2019. **Figure S2.** The distribution of number and ASR of incidence and death of neonatal encephalopathy due to birth asphyxia and trauma in SDI areas, and geographic regions from1990 to 2019. **Figure S3.** The distribution of number and ASR of incidence and death of neonatal sepsis and other neonatal infections in SDI areas, and geographic regions from1990 to 2019. **Figure S4.** The distribution of number and ASR of incidence and death of hemolytic disease and other neonatal jaundice in SDI areas, and geographic regions from1990 to 2019. **Figure S5.** The distribution of percentage changes in number of neonatal disorders incidence in specific causes at the national level. **Figure S6.** The distribution of EAPCs of neonatal disorders incidence in specific causes at the national level. **Figure S7.** The distribution of percentage changes in number of death caused by neonatal disorders in specific causes at the national level. **Figure S8.** The distribution of percentage changes in number of death caused by neonatal disorders in specific causes at the national level. **Table S1.** the age-standardized rate of incidence and death of neonatal disorders at national level in 2019, and percentage changes in number and the EAPCs from 1990 to 2019. **Table S2.** The percentage changes in number and the EAPCs of neonatal disorders incidence due to specific causes in global, SDI areas and geographic regions from 1990 to 2019. **Table S3.** the percentage changes in number and the EAPCs of incidence of neonatal disorders due to etiologies at national level from 1990 to 2019. **Table S4.** The percentage changes in number and the EAPCs of death caused by neonatal disorders in etiologies in global, SDI areas and geographic regions from 1990 to 2019. **Table S5.** the percentage changes in number and the EAPCs of death caused by neonatal disorders in etiologies at national level from 1990 to 2019.

## Data Availability

All data were included in this article and its supplementary files. Public link to the database of GBD study is open, and data can be downloaded via the Global Health Data Exchange (GHDx) (http://ghdx.healthdata.org). which is supported by the Institute for Health Metrics and Evaluation (IHME), University of Washington, USA.

## Abbreviations

GBD: Global Burden of Disease
ND: Neonatal disorders
NPB: Neonatal preterm birth
NE: Neonatal encephalopathy due to birth asphyxia and trauma
NS: Neonatal sepsis and other neonatal infections
HD: Hemolytic disease and other neonatal jaundice
UN: United Nations
SDG: Sustainable Development Goal
EAPC: Estimated annual percentage change
GHDx: Global health data exchange
SDI: Socio-demographic index
ASR: Age-standardized rate
CI: Confidence interval
UI: Uncertainty interval
