# Supplementary material for: Global trends in incidence and death of neonatal disorders and its specific causes in 204 countries/territories during 1990–2019
Source: BMC Public Health. 2022 Feb 19;22:360. doi: 10.1186/s12889-022-12765-1 (PMC8858498; doi:10.1186/s12889-022-12765-1)
Supplement: Supplementary file 1 — Additional file 1: Figure S1. The distribution of number and ASR of incidence and death of neonatal preterm birth in SDI areas, and geographic regions from1990 to 2019. Figure S2. The distribution of number and ASR of incidence and death of neonatal encephalopathy due to birth asphyxia and trauma in SDI areas, and geographic regions from1990 to 2019. Figure S3. The distribution of number and ASR of incidence and death of neonatal sepsis and other neonatal infections in SDI areas, and geographic regions from1990 to 2019. Figure S4. The distribution of number and ASR of incidence and death of hemolytic disease and other neonatal jaundice in SDI areas, and geographic regions from1990 to 2019. Figure S5. The distribution of percentage changes in number of neonatal disorders incidence in specific causes at the national level. Figure S6. The distribution of EAPCs of neonatal disorders incidence in specific causes at the national level. Figure S7. The distribution of percentage changes in number of death caused by neonatal disorders in specific causes at the national level. Figure S8. The distribution of percentage changes in number of death caused by neonatal disorders in specific causes at the national level. Table S1. the age-standardized rate of incidence and death of neonatal disorders at national level in 2019, and percentage changes in number and the EAPCs from 1990 to 2019. Table S2. The percentage changes in number and the EAPCs of neonatal disorders incidence due to specific causes in global, SDI areas and geographic regions from 1990 to 2019. Table S3. the percentage changes in number and the EAPCs of incidence of neonatal disorders due to etiologies at national level from 1990 to 2019. Table S4. The percentage changes in number and the EAPCs of death caused by neonatal disorders in etiologies in global, SDI areas and geographic regions from 1990 to 2019. Table S5. the percentage changes in number and the EAPCs of death caused by neonatal disorders in etiologies [file 12889_2022_12765_MOESM1_ESM.docx]

**Supplementary figure 1**. the distribution of number and ASR of incidence and death of neonatal preterm birth in SDI areas, and geographic regions from1990 to 2019. A. the ASIR in SDI areas; B. the incident number in geographic regions; C. the ASDR in SDI areas; D. the death number in geographic regions. ASIR, age-standardized incidence rate; ASDR, age-standardized death rate; SDI, sociodemographic index.


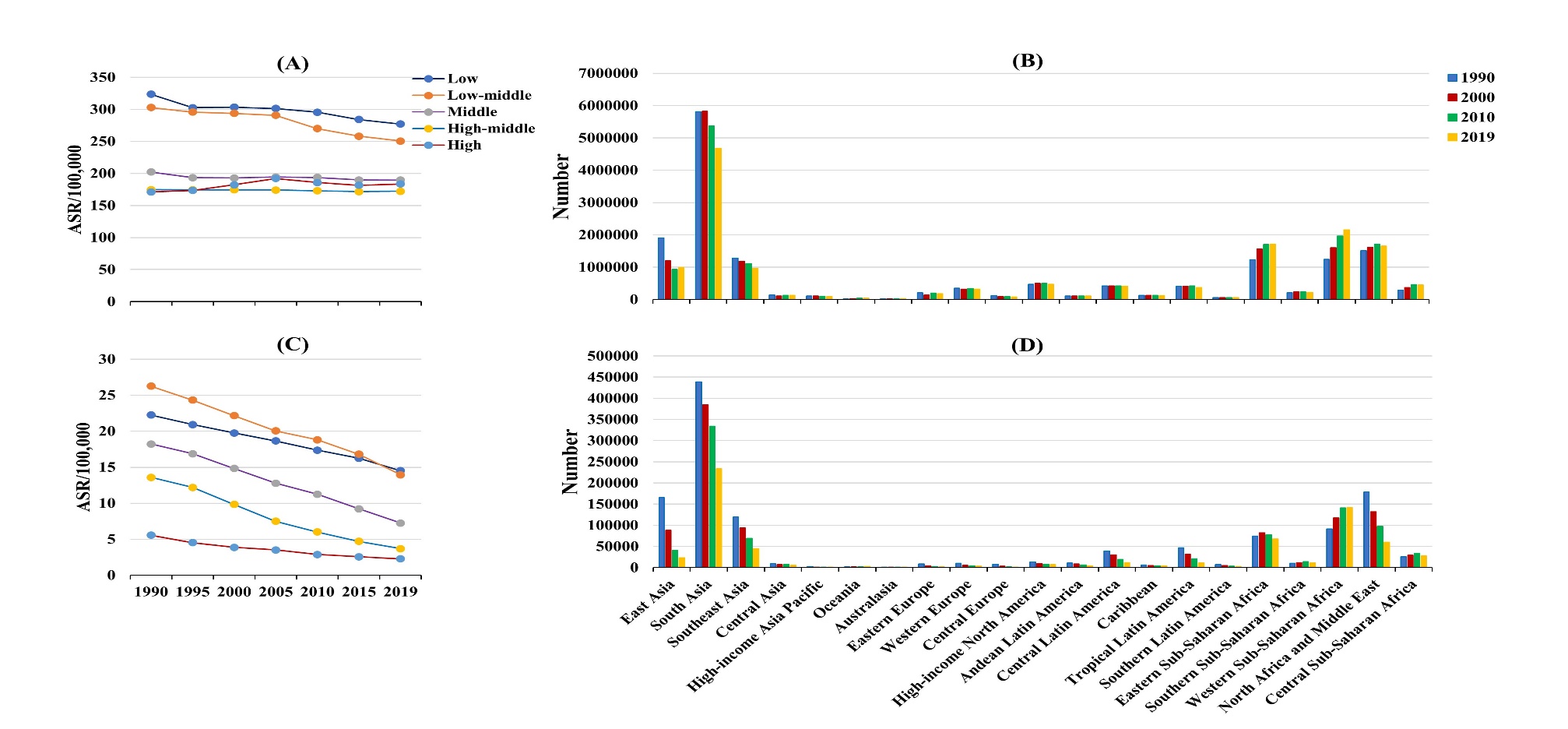


**Supplementary figure 2**. the distribution of number and ASR of incidence and death of neonatal encephalopathy due to birth asphyxia and trauma in SDI areas, and geographic regions from1990 to 2019. A. the ASIR in SDI areas; B. the incident number in geographic regions; C. the ASDR in SDI areas; D. the death number in geographic regions. ASIR, age-standardized incidence rate; ASDR, age-standardized death rate; SDI, sociodemographic index.


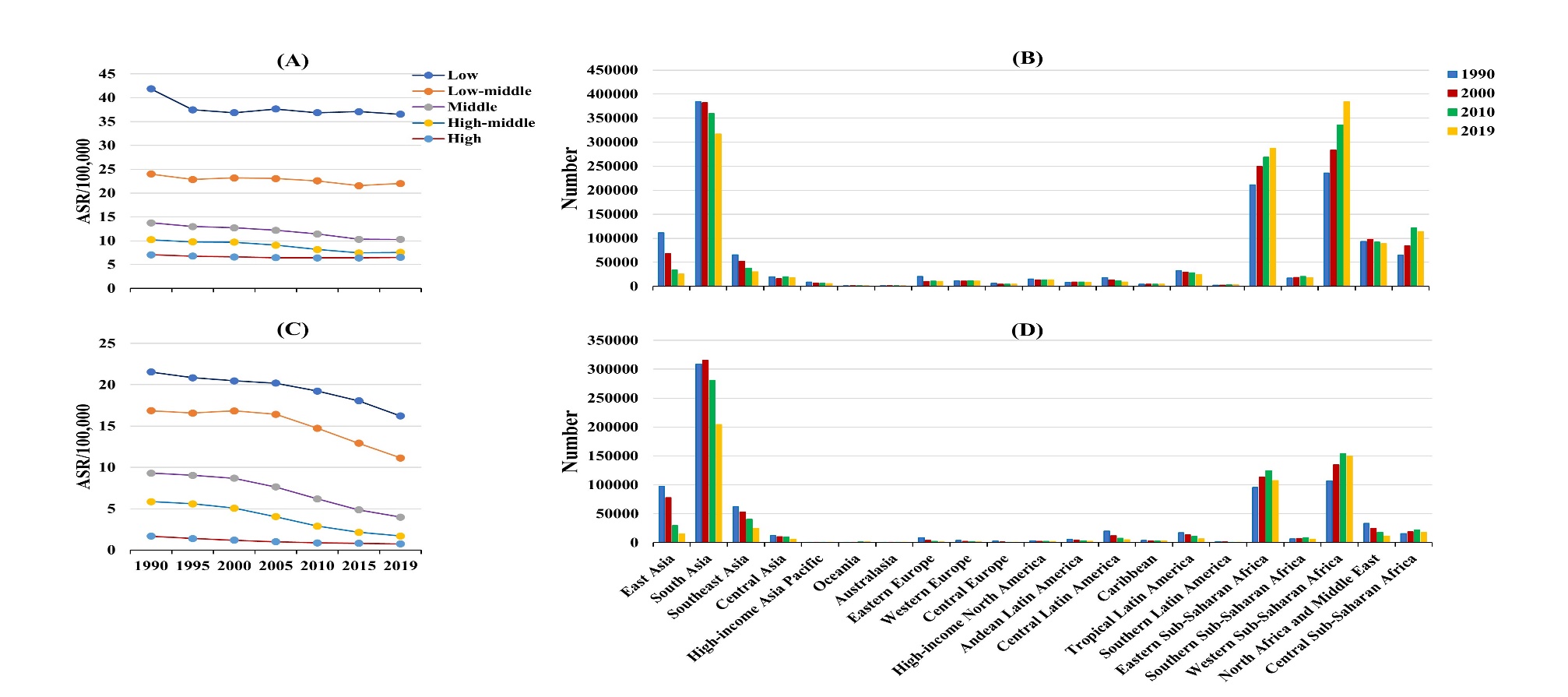


**Supplementary figure 3**. the distribution of number and ASR of incidence and death of neonatal sepsis and other neonatal infections in SDI areas, and geographic regions from1990 to 2019. A. the ASIR in SDI areas; B. the incident number in geographic regions; C. the ASDR in SDI areas; D. the death number in geographic regions. ASIR, age-standardized incidence rate; ASDR, age-standardized death rate; SDI, sociodemographic index.


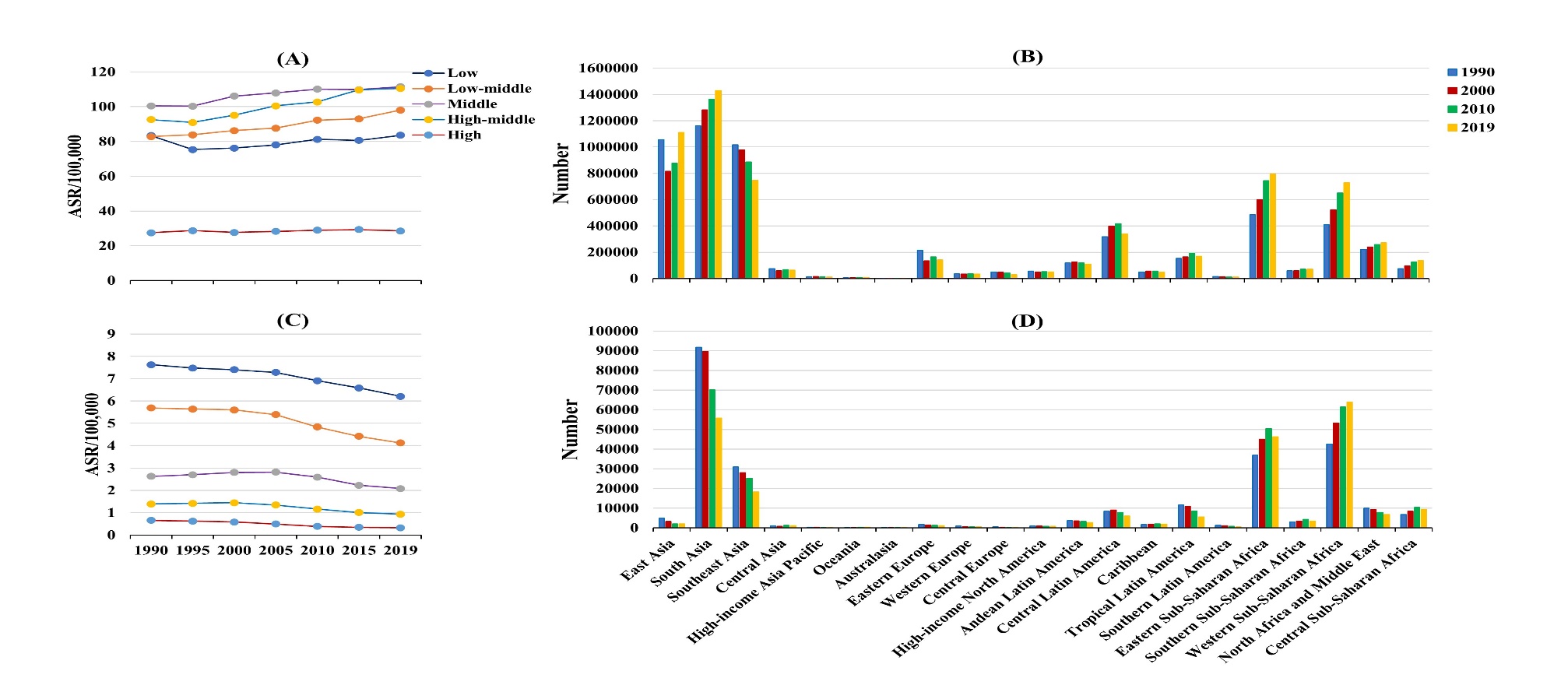


**Supplementary figure 4**. the distribution of number and ASR of incidence and death of hemolytic disease and other neonatal jaundice in SDI areas, and geographic regions from1990 to 2019. A. the ASIR in SDI areas; B. the incident number in geographic regions; C. the ASDR in SDI areas; D. the death number in geographic regions. ASIR, age-standardized incidence rate; ASDR, age-standardized death rate; SDI, sociodemographic index.


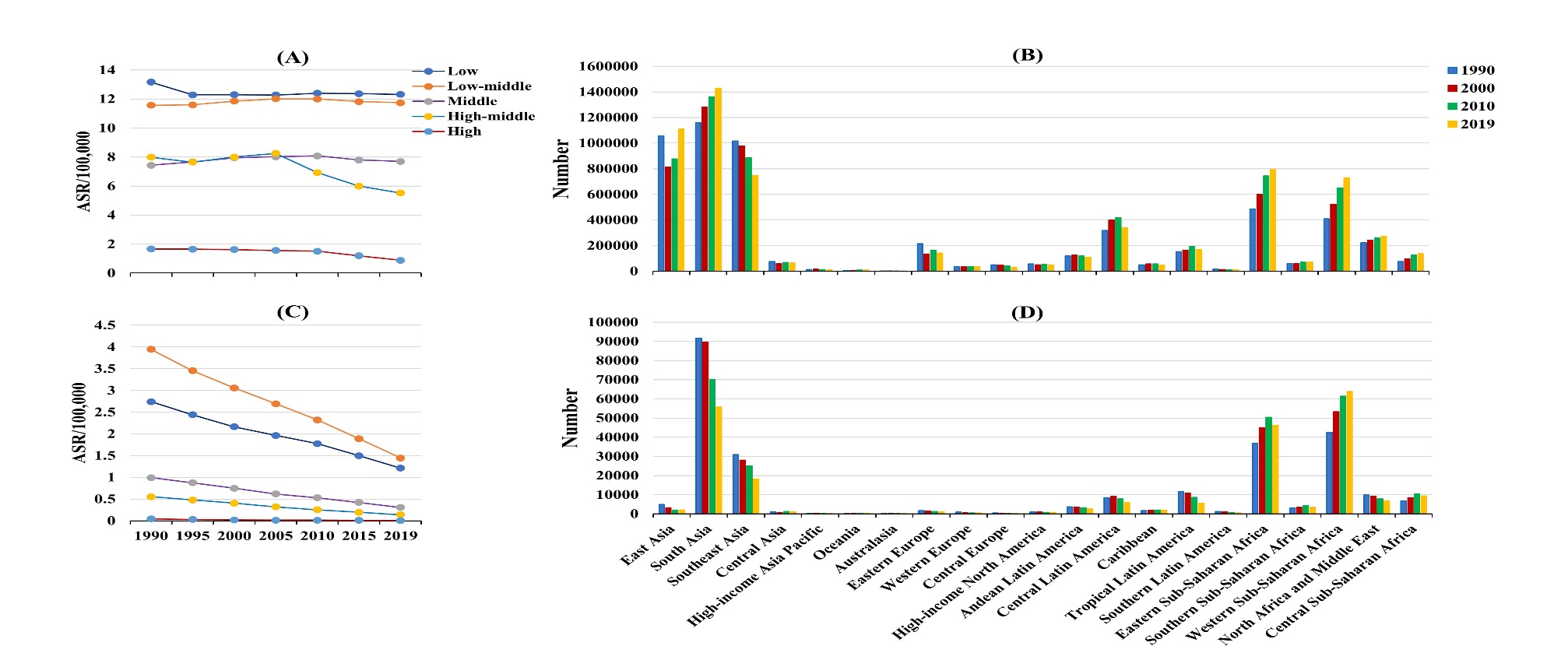


**Supplementary figure 5**. the distribution of percentage changes in number of neonatal disorders incidence in specific causes at the national level. A, B, C, and D respectively presented neonatal preterm birth, neonatal encephalopathy due to birth asphyxia and trauma, neonatal sepsis and other neonatal infections, and hemolytic disease and other neonatal jaundice. Countries/territories with an extreme value were annotated. ASIR, age-standardized incidence rate.


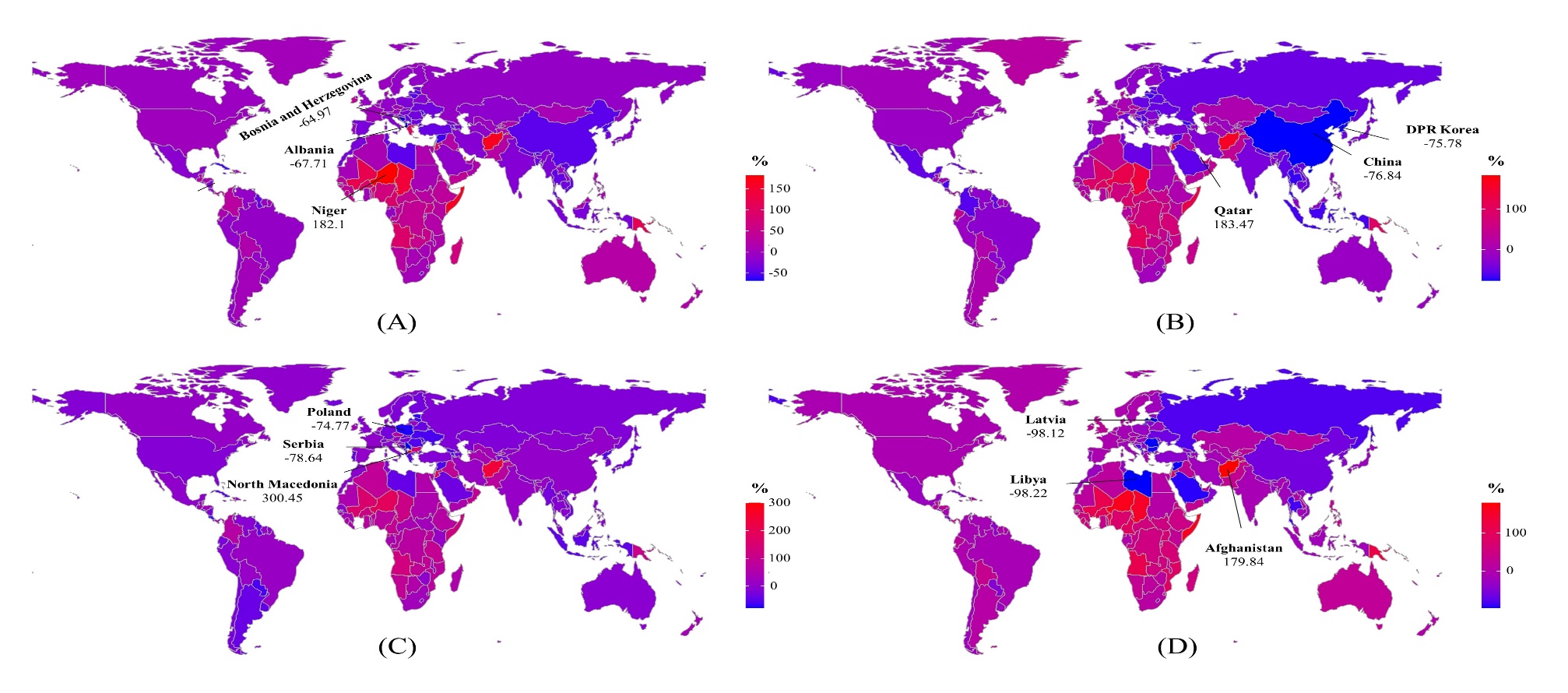


**Supplementary figure 6**. the distribution of EAPCs of neonatal disorders incidence in specific causes at the national level. A, B, C, and D respectively presented neonatal preterm birth, neonatal encephalopathy due to birth asphyxia and trauma, neonatal sepsis and other neonatal infections, and hemolytic disease and other neonatal jaundice. Countries/territories with an extreme value were annotated. ASIR, age-standardized incidence rate.


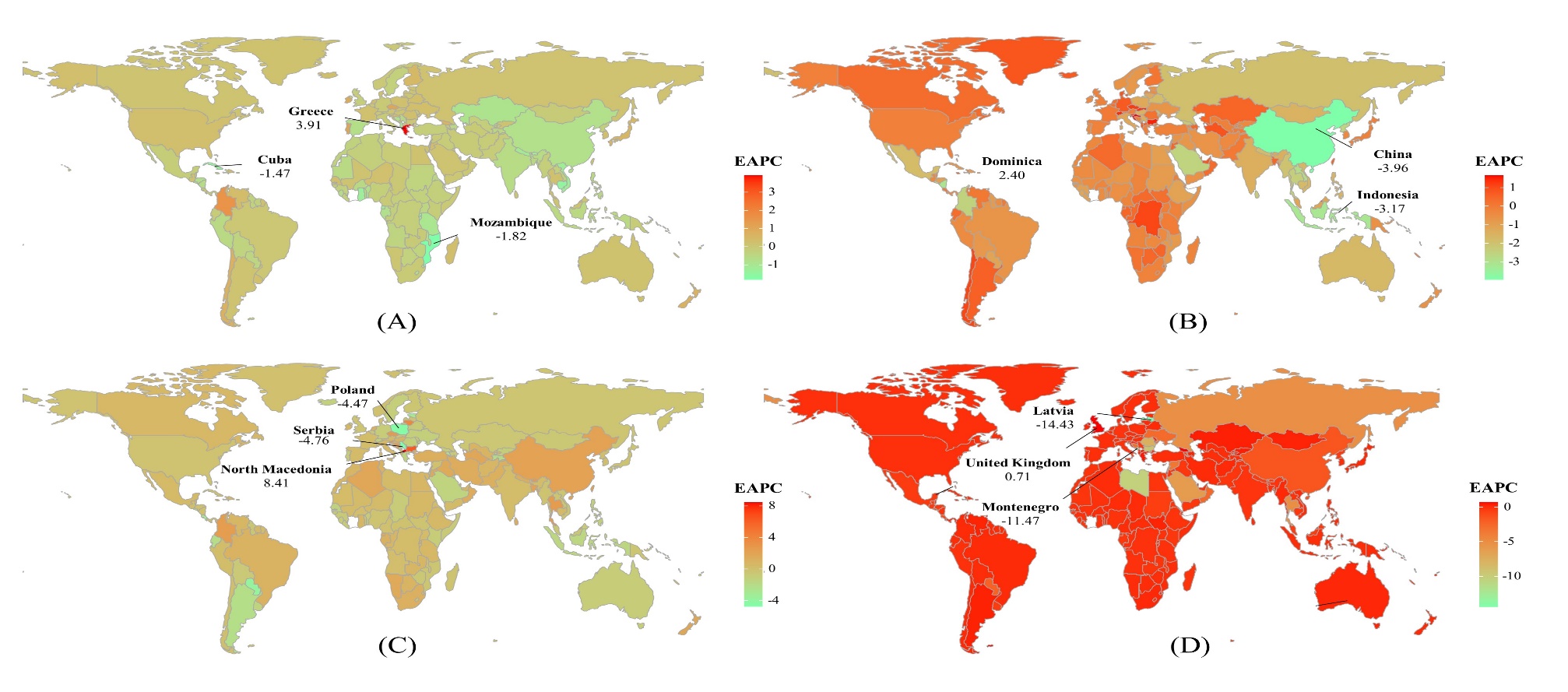


**Supplementary figure 7**. the distribution of percentage changes in number of death caused by neonatal disorders in specific causes at the national level. A, B, C, and D respectively presented neonatal preterm birth, neonatal encephalopathy due to birth asphyxia and trauma, neonatal sepsis and other neonatal infections, and hemolytic disease and other neonatal jaundice. Countries/territories with an extreme value were annotated. ASDR, age-standardized death rate.


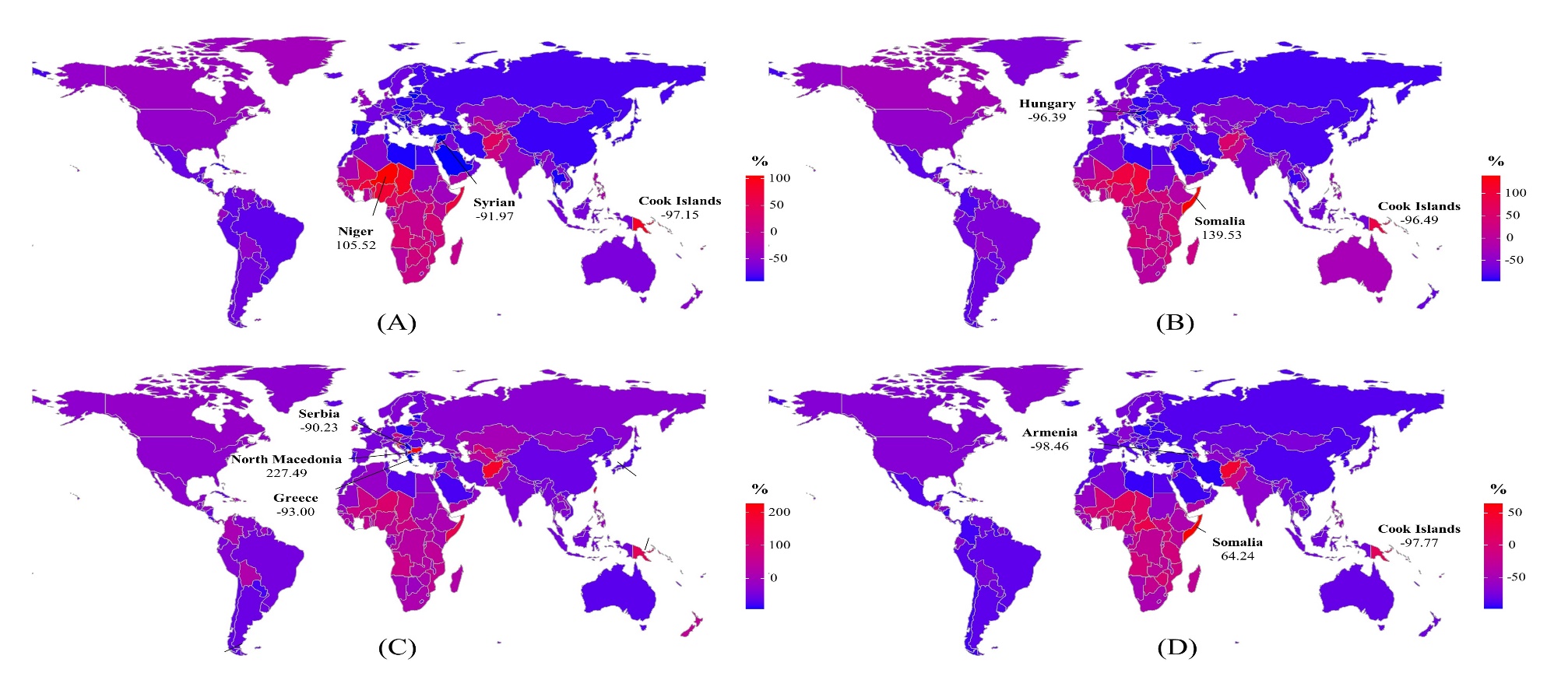


**Supplementary figure 8**. the distribution of percentage changes in number of death caused by neonatal disorders in specific causes at the national level. A, B, C, and D respectively presented neonatal preterm birth, neonatal encephalopathy due to birth asphyxia and trauma, neonatal sepsis and other neonatal infections, and hemolytic disease and other neonatal jaundice. Countries/territories with an extreme value were annotated. ASDR, age-standardized death rate.

**
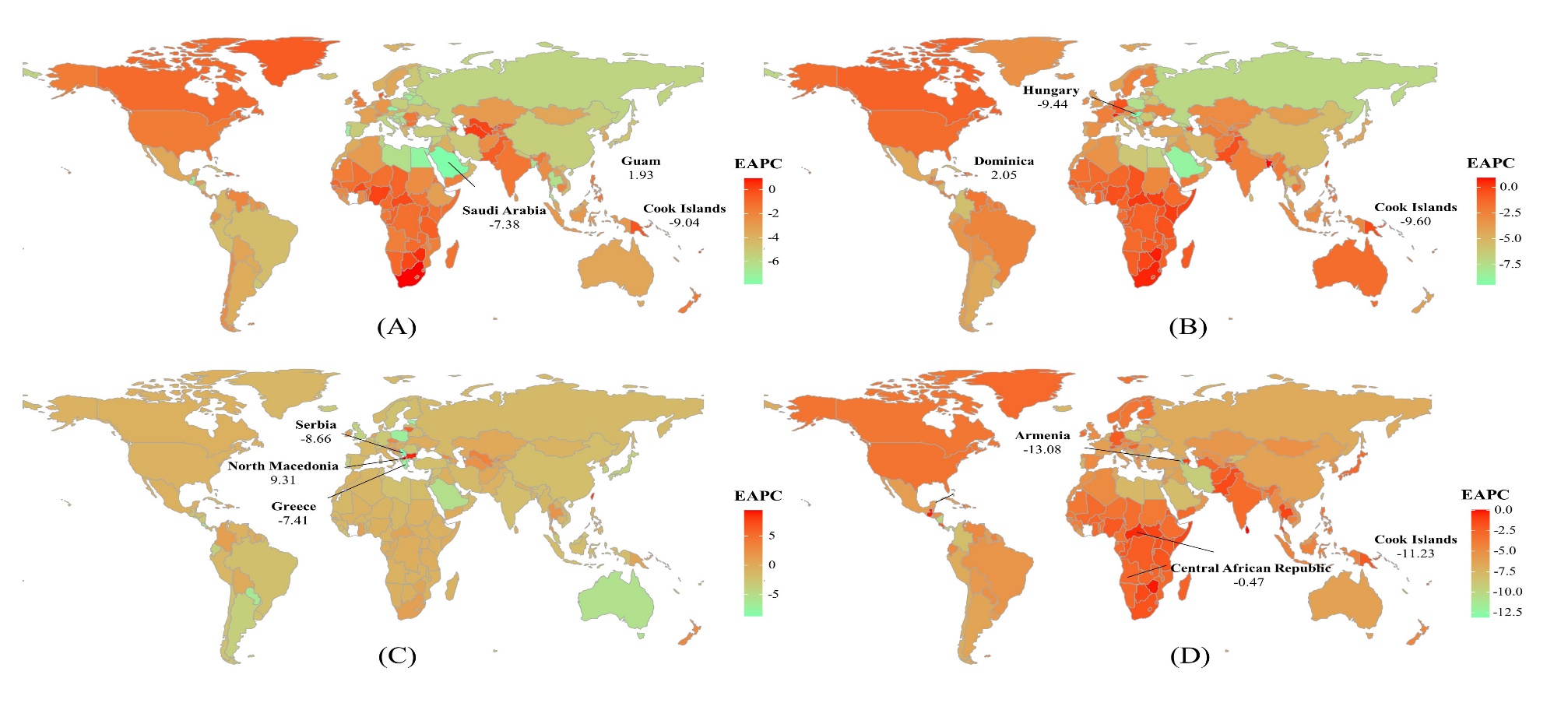
**

**Supplementary table 1**. the age-standardized rate of incidence and death of neonatal disorders at national level in 2019, and percentage changes in number and the EAPCs from 1990 to 2019.

|  | **Incidence** | | | **Death** | | |
| --- | --- | --- | --- | --- | --- | --- |
| **Characteristics** | ASR/100,000  (95% UI) | Percentage  (%) | EAPC  (95%CI) | ASR/100,000  (95% UI) | Percentage  (%) | EAPC  (95%CI) |
| Afghanistan | 481.18(24.78–34.53) | 168.48 | -0.08(-0.26–0.1) | 33.46(25.4–43.13) | 51.81 | -1.83(-2.04–-1.62) |
| Albania | 209.14(68.41–137.9) | -63.42 | -0.58(-0.67–-0.49) | 9.59(6.56–14.29) | -81.46 | -2.97(-3.75–-2.19) |
| Algeria | 231.98(92.95–182.64) | 22.72 | 0.18(0.12–0.23) | 20.82(16.3–26.01) | -51.17 | -2.8(-2.93–-2.67) |
| American Samoa | 214.89(217.27–372.02) | -45.48 | -0.45(-0.52–-0.38) | 8.04(5.74–10.75) | -65.68 | -2.13(-2.33–-1.93) |
| Andorra | 209.62(85.23–168.61) | 3.3 | 0.6(0.45–0.75) | 1.54(1.13–2.04) | -69.05 | -3.08(-3.49–-2.66) |
| Angola | 381.5(394.47–913.24) | 100.13 | -0.28(-0.31–-0.25) | 34.37(28.45–40.6) | 28 | -1.82(-1.97–-1.68) |
| Antigua and Barbuda | 287.82(250.89–377.22) | -21.25 | 0.35(0.28–0.41) | 9.13(6.46–12.56) | -55.82 | -2.24(-2.53–-1.95) |
| Argentina | 164.69(275.7–408.93) | -5.17 | -0.22(-0.24–-0.19) | 9.8(7.63–12.28) | -65.21 | -3.68(-3.77–-3.58) |
| Armenia | 404.35(179.36–273.31) | -62.5 | -1.27(-1.47–-1.07) | 8.58(6.49–11.12) | -86.52 | -5.04(-5.4–-4.69) |
| Australia | 168.88(55.7–72.27) | 22.29 | 0.06(-0.01–0.14) | 3.28(2.74–3.9) | -44.19 | -2.29(-2.46–-2.11) |
| Austria | 161.44(67.77–96.65) | -1.13 | 0.11(0.1–0.13) | 2.69(2.23–3.16) | -63.84 | -2.93(-3.15–-2.72) |
| Azerbaijan | 298.62(139.57–263.71) | -31.29 | -0.45(-0.55–-0.35) | 26.5(21.59–32.27) | -40.87 | -0.66(-1.26–-0.05) |
| Bahamas | 352.48(270.7–456.68) | -26.22 | -0.09(-0.14–-0.04) | 10.47(7.82–13.64) | -60.97 | -1.91(-2.16–-1.67) |
| Bahrain | 275.34(56.8–99.68) | 37.17 | 1.69(1.55–1.84) | 5.22(4–6.8) | -76.66 | -4.29(-4.9–-3.67) |
| Bangladesh | 604.32(100.78–277.14) | -24.56 | 0.33(0.24–0.43) | 31.18(23.43–40.08) | -72.09 | -2.68(-2.96–-2.39) |
| Barbados | 439.95(308.51–484.21) | -21.99 | 0.61(0.53–0.68) | 14.36(9.81–20.37) | -53.92 | -1.21(-1.28–-1.14) |
| Belarus | 285.47(142.45–272.77) | -32.6 | -0.47(-0.56–-0.38) | 3.22(2.23–4.21) | -81.22 | -5.1(-5.39–-4.81) |
| Belgium | 189.99(68.32–88.47) | -1.46 | -0.03(-0.08–0.02) | 2.88(2.31–3.51) | -60.08 | -2.86(-3.16–-2.55) |
| Belize | 343.23(470.62–678.98) | 29.04 | 0.14(0.06–0.22) | 15.45(12.43–18.7) | -36.57 | -2.39(-2.45–-2.32) |
| Benin | 373.68(428.27–861.22) | 83.9 | -0.65(-0.72–-0.58) | 52.39(42.23–65.47) | 52.21 | -1.02(-1.07–-0.97) |
| Bermuda | 290.02(72.57–119.44) | -37.14 | 0.41(0.38–0.44) | 4.69(3.21–6.61) | -74.03 | -2.02(-2.61–-1.41) |
| Bhutan | 408.83(131.29–352.43) | -44.06 | -0.32(-0.37–-0.27) | 36.53(26.02–50.01) | -74.85 | -3.07(-3.2–-2.93) |
| Bolivia  (Plurinational State of) | 322.49(474.07–941.17) | 14.68 | -0.48(-0.55–-0.42) | 24.63(18.88–31.17) | -41.23 | -2.68(-2.8–-2.56) |
| Bosnia and Herzegovina | 258.73(120.09–235.69) | -59.96 | -0.22(-0.37–-0.07) | 5.67(4.56–6.92) | -89.72 | -5.25(-5.76–-4.73) |
| Botswana | 457.77(468.54–1263.81) | 16.35 | -0.06(-0.09–-0.02) | 36.78(26.73–51.59) | 4.64 | -0.39(-0.61–-0.17) |
| Brazil | 380.42(249.59–309.88) | -5.41 | 0.24(0.19–0.29) | 19.9(15.73–24.66) | -63.86 | -2.96(-3.12–-2.8) |
| Brunei Darussalam | 182.69(245.47–395.91) | 6.57 | 0.61(0.56–0.65) | 7.5(5.66–9.93) | -8.7 | 0.53(0.28–0.78) |
| Bulgaria | 228.53(189.85–389.9) | -25.74 | 0.89(0.8–0.97) | 5.37(4.13–6.89) | -69.71 | -2.94(-3.25–-2.63) |
| Burkina Faso | 488.96(507.68–922.64) | 97.37 | -0.37(-0.44–-0.3) | 43.04(33.16–55.38) | 52.55 | -0.96(-1.11–-0.81) |
| Burundi | 398.39(504.47–1231.79) | 62.15 | -0.34(-0.44–-0.25) | 39.51(32.29–48.81) | 13.28 | -1.6(-1.74–-1.45) |
| Cabo Verde | 411.85(288.33–535.6) | -4.63 | 0.17(0.15–0.19) | 17.91(12.85–24.3) | -46.64 | -1.64(-2.21–-1.06) |
| Cambodia | 387.95(215.02–460.08) | -29.95 | -0.91(-1.05–-0.77) | 25.28(19.05–33.9) | -53.39 | -1.97(-2.16–-1.78) |
| Cameroon | 352.14(403.6–955.34) | 89.92 | -0.09(-0.12–-0.06) | 36.34(28.13–45.12) | 34.66 | -1.23(-1.29–-1.17) |
| Canada | 188.82(66.46–86.75) | -1.96 | 0.22(0.19–0.24) | 5.04(4.4–5.76) | -27.11 | -0.43(-0.61–-0.25) |
| Central African Republic | 407.43(567.83–1427.5) | 50.39 | -0.1(-0.13–-0.06) | 56.94(43.72–73.31) | 34.15 | -0.29(-0.41–-0.17) |
| Chad | 347.55(523.81–1026.84) | 135.57 | -0.2(-0.26–-0.14) | 46.06(36.87–57.41) | 81.52 | -1.04(-1.14–-0.93) |
| Chile | 171.76(188.53–253.96) | -12.82 | 0.63(0.56–0.7) | 5.44(3.9–7.2) | -66.93 | -2.1(-2.38–-1.82) |
| China | 290.9(86.9–202.91) | -30.28 | 0.39(0.29–0.48) | 6.22(5.32–7.19) | -84.28 | -5.1(-5.57–-4.64) |
| Colombia | 437.12(199.02–347.9) | 37.76 | 1.78(1.56–1.99) | 11.16(7.52–15.66) | -68.54 | -3.41(-3.62–-3.2) |
| Comoros | 571.8(443.12–1051.25) | -19.59 | -0.07(-0.11–-0.02) | 48.04(36.74–61.74) | -46.78 | -1.46(-1.67–-1.25) |
| Congo | 381.88(473.04–1086.01) | 41.6 | 0.01(-0.03–0.05) | 29.08(23.94–34.61) | 4.55 | -0.92(-1.09–-0.75) |
| Cook Islands | 265.18(74.97–146.52) | -44.82 | -0.13(-0.15–-0.12) | 1.14(0.68–1.68) | -95.88 | -9.04(-9.69–-8.38) |
| Costa Rica | 214.06(164.39–298.15) | -37.52 | -0.94(-1.02–-0.86) | 7.45(5.08–10.2) | -59.09 | -2.26(-2.39–-2.12) |
| Croatia | 364.12(80.3–142.98) | -8.07 | 0.82(0.15–1.5) | 3.62(2.67–4.82) | -80.71 | -4.25(-4.44–-4.07) |
| Cuba | 212.36(184.66–304.63) | -56.29 | -1.05(-1.15–-0.94) | 3.56(2.47–4.53) | -78.5 | -3.09(-3.38–-2.8) |
| Cyprus | 161.61(49.31–85.95) | 10.75 | 0.07(0.03–0.12) | 3.36(2.45–4.55) | -77.06 | -5.69(-6.18–-5.2) |
| Czechia | 273.53(105.37–168.05) | 19.93 | 1.51(1.15–1.86) | 2.44(1.78–3.19) | -81.46 | -5.22(-5.8–-4.63) |
| Cote d'Ivoire | 512.52(385.3–795.51) | 41.26 | -0.25(-0.32–-0.18) | 54.55(43.71–66.44) | 24.76 | -0.66(-0.86–-0.45) |
| Democratic People's Republic of Korea | 205.36(175.29–415.39) | -54.51 | -0.28(-0.33–-0.22) | 11.22(8.65–14.58) | -86.36 | -4.04(-4.41–-3.67) |
| Democratic Republic of  the Congo | 318.42(440.04–957.89) | 53.33 | 0.11(0.01–0.22) | 30.77(24.42–38.51) | -0.89 | -1.33(-1.48–-1.18) |
| Denmark | 139.58(82.88–128.45) | 4.17 | 0.2(0.18–0.22) | 3.59(2.68–4.57) | -43.1 | -1.33(-1.56–-1.1) |
| Djibouti | 426.54(387.57–1185.74) | 51.65 | -0.16(-0.22–-0.1) | 38.26(30–48.64) | 18.92 | -1.04(-1.19–-0.88) |
| Dominica | 419.77(401.54–695.38) | -46.81 | 0.48(0.43–0.54) | 28.64(19.88–40.47) | -35.42 | 1.27(1.02–1.53) |
| Dominican Republic | 567.52(281.44–573.79) | 7.19 | 0.37(0.25–0.5) | 30.22(21.11–41.43) | -38.11 | -1.18(-1.33–-1.04) |
| Ecuador | 288.2(286.38–523.03) | -3.46 | -1.03(-1.26–-0.81) | 12.88(8.76–18.04) | -45.98 | -3.12(-3.67–-2.57) |
| Egypt | 408.55(30.67–65.66) | 17.2 | 0.18(0.12–0.24) | 5.23(3.01–7.91) | -81.22 | -6.43(-7.1–-5.75) |
| El Salvador | 304.46(328.34–631.33) | -42.16 | -0.71(-0.81–-0.6) | 8.9(6–12.65) | -83.19 | -4.41(-4.61–-4.21) |
| Equatorial Guinea | 373.86(302.02–846.19) | 45.96 | -0.54(-0.63–-0.45) | 24.48(15.6–35.26) | -11.12 | -2.28(-2.54–-2.02) |
| Eritrea | 453.29(614.4–1424.77) | 37.23 | -0.3(-0.38–-0.22) | 33.19(23.87–46.35) | 10.43 | -0.89(-1–-0.79) |
| Estonia | 177.41(119.98–213.43) | -51.06 | -1.15(-1.49–-0.81) | 1.64(1.04–2.09) | -92.35 | -8.12(-8.46–-7.77) |
| Eswatini | 292.23(400.57–1360.64) | -13.43 | -0.22(-0.26–-0.19) | 31.28(25.11–38.75) | -28.37 | -0.78(-0.9–-0.67) |
| Ethiopia | 453.58(343.9–810.21) | 40.75 | -0.14(-0.27–-0.01) | 44.31(34.92–56.99) | -15.95 | -1.79(-1.98–-1.6) |
| Fiji | 248.36(283.37–939.18) | -15.74 | -0.44(-0.52–-0.35) | 17.72(12.55–24.26) | -27.3 | -0.97(-1.21–-0.74) |
| Finland | 139.82(38.57–54.33) | -13.91 | 0.39(0.32–0.46) | 1.57(1.09–1.96) | -76.8 | -4.03(-4.25–-3.82) |
| France | 120.79(71.37–93.13) | 0.45 | 0.24(0.21–0.26) | 3.31(2.73–3.93) | -43.28 | -1.33(-1.52–-1.13) |
| Gabon | 408.49(318.3–731.05) | 3.02 | -0.24(-0.34–-0.15) | 24.86(17.36–34.84) | -37.6 | -1.63(-1.9–-1.36) |
| Gambia | 419.17(356.56–732.28) | 28.65 | -0.6(-0.63–-0.56) | 33.57(24.3–45.99) | -18.63 | -1.95(-2.12–-1.78) |
| Georgia | 340.85(203.26–320.31) | -43.68 | -0.25(-0.52–0.03) | 12.55(9.69–15.99) | -82.03 | -4.55(-5.28–-3.82) |
| Germany | 246.97(77.18–97.32) | -12.84 | -0.08(-0.14–-0.02) | 2.97(2.55–3.33) | -58.87 | -1.97(-2.14–-1.8) |
| Ghana | 399.23(344.09–709.92) | 27.69 | -0.51(-0.56–-0.45) | 39.26(27.67–53.82) | 8.9 | -0.9(-1.18–-0.61) |
| Greece | 241.78(77.46–96.73) | 77.04 | 3.35(3.13–3.56) | 3.36(2.67–4.16) | -72.03 | -3.88(-4.55–-3.21) |
| Greenland | 223.6(224.13–393.34) | -35.98 | -0.05(-0.15–0.05) | 9.31(6–13.97) | -81.47 | -4.52(-4.74–-4.3) |
| Grenada | 348.87(427.55–613.68) | -23.45 | 0.31(0.29–0.32) | 15.82(11.01–22.2) | -54.82 | -1.3(-1.57–-1.02) |
| Guam | 284.59(142.79–236.93) | -15.28 | -0.14(-0.39–0.1) | 13.11(10.11–16.66) | 5.24 | 1.07(0.91–1.24) |
| Guatemala | 400.78(395–734.61) | 21.86 | 0.11(0.05–0.16) | 14.31(10.11–19.56) | -65.22 | -4.76(-5–-4.52) |
| Guinea | 379.58(813.77–1527.84) | 37.22 | -0.47(-0.53–-0.41) | 47.81(36.93–60.39) | -13.07 | -2.21(-2.34–-2.09) |
| Guinea-Bissau | 499.65(586.74–1325.59) | 21.49 | -0.35(-0.37–-0.33) | 51.97(42.88–62.06) | -17.98 | -1.61(-1.72–-1.49) |
| Guyana | 499.78(502.45–914.26) | -41.41 | -0.35(-0.4–-0.3) | 27.94(19.56–38.37) | -69.17 | -2.32(-2.48–-2.15) |
| Haiti | 484.14(419.09–1364.03) | 26.87 | -0.28(-0.31–-0.24) | 37.05(27.65–47.45) | 31.66 | 0.03(-0.05–0.11) |
| Honduras | 357.43(210.79–530.99) | 16.72 | -0.53(-0.6–-0.47) | 14.34(10.75–18.4) | -41.92 | -2.77(-2.98–-2.55) |
| Hungary | 265.54(132.84–217.74) | -29.23 | 0.25(0.17–0.33) | 3.38(2.23–4.6) | -87.7 | -5.65(-5.85–-5.46) |
| Iceland | 159.88(45.72–64.42) | -5.09 | 0.15(0.12–0.18) | 2.05(1.24–3.26) | -67.6 | -3.87(-4.22–-3.53) |
| India | 365.36(184.74–325.15) | -20.21 | -0.5(-0.58–-0.43) | 38.91(31.9–48.38) | -48.64 | -1.67(-1.83–-1.51) |
| Indonesia | 289.49(143.05–352.41) | -38.98 | -1.04(-1.07–-1.01) | 20.67(16.16–25.96) | -62.72 | -2.65(-2.78–-2.52) |
| Iran  (Islamic Republic of) | 274.09(38.94–62.2) | -17.55 | 0.18(0.13–0.23) | 11.29(8.98–13.88) | -85.65 | -5.12(-5.62–-4.62) |
| Iraq | 377.13(49.79–95.2) | 32.96 | 0.14(0.05–0.23) | 15.79(11.15–21.96) | -58.2 | -3.73(-4.1–-3.37) |
| Ireland | 146.87(63.79–105.93) | 41.18 | 0.83(0.65–1.02) | 2.54(1.94–3.25) | -53.34 | -2.97(-3.27–-2.67) |
| Israel | 154.28(56.03–82.16) | 93.15 | 0.1(0.07–0.12) | 2.41(1.87–3.03) | -55.53 | -4.78(-4.92–-4.64) |
| Italy | 194.7(40.66–71.73) | -19.4 | -0.04(-0.12–0.04) | 2.81(2.3–3.29) | -75.98 | -3.95(-4.22–-3.67) |
| Jamaica | 436.62(379.85–672.19) | -27.2 | 0.66(0.55–0.78) | 23.04(16.23–31.63) | -50.43 | -1.06(-1.21–-0.92) |
| Japan | 147.81(67.46–99.86) | -19.31 | 0.44(0.35–0.53) | 1.07(0.86–1.23) | -77.35 | -4.06(-4.19–-3.94) |
| Jordan | 351.76(39.79–73.64) | 101.29 | 0.42(0.33–0.51) | 13.34(9.69–18.77) | -22.09 | -3.09(-3.27–-2.91) |
| Kazakhstan | 253.23(217.61–317.76) | -14.45 | -0.59(-0.66–-0.53) | 9.33(6.61–12.29) | -50.55 | -2.49(-3.2–-1.78) |
| Kenya | 360.55(266.45–641.69) | 23.53 | -0.34(-0.43–-0.25) | 28.47(22.82–35.22) | 5.03 | -0.62(-0.7–-0.54) |
| Kiribati | 238.53(1534.43–2781.26) | 10.09 | -0.16(-0.19–-0.13) | 31.09(21.43–43.43) | -33.65 | -1.96(-2.05–-1.86) |
| Kuwait | 252.82(33.14–62.29) | 51.01 | -0.03(-0.1–0.04) | 6.97(5.35–9.02) | -20.95 | -1.02(-1.48–-0.55) |
| Kyrgyzstan | 225.44(233.19–347.31) | 11.78 | -0.16(-0.22–-0.11) | 18.7(16.07–21.48) | -39.21 | -1.71(-2.21–-1.2) |
| Lao People's Democratic Republic | 337.86(180.22–437.83) | -9.6 | -0.39(-0.48–-0.3) | 37.09(29.12–45.85) | -53.33 | -2.82(-3.03–-2.62) |
| Latvia | 191.98(96.35–188.74) | -56.16 | -0.53(-0.63–-0.44) | 2.93(1.88–3.73) | -86.68 | -5.61(-6.07–-5.14) |
| Lebanon | 265.4(48.58–99.59) | 5.79 | 0.37(0.27–0.48) | 7.87(5.1–11.51) | -75.68 | -4.65(-4.75–-4.56) |
| Lesotho | 369.58(542.05–1883.99) | -33.76 | -0.55(-0.56–-0.53) | 51.17(34.89–69.44) | -43.4 | -1.09(-1.22–-0.96) |
| Liberia | 468.2(413.31–875.05) | 43.34 | -0.42(-0.53–-0.3) | 34.84(24.8–48.07) | -33.85 | -3.02(-3.2–-2.85) |
| Libya | 246.25(82.41–167.15) | -53.17 | -0.44(-0.55–-0.33) | 8.83(6.12–11.78) | -87.78 | -5.38(-5.56–-5.19) |
| Lithuania | 214.98(126.83–212.66) | -38.83 | 1.2(0.97–1.43) | 2.2(1.53–2.66) | -88.99 | -5.95(-6.33–-5.56) |
| Luxembourg | 159.04(46.35–69.27) | 40.08 | 0.17(0.13–0.21) | 1.97(1.21–3.12) | -61.51 | -4.16(-4.62–-3.7) |
| Madagascar | 488.91(462.52–1047.43) | 62.82 | 0.14(0.05–0.22) | 30.54(23.62–38.82) | 6.36 | -1.22(-1.3–-1.13) |
| Malawi | 462.25(531.31–1198.96) | 5.47 | -0.55(-0.65–-0.44) | 38.96(29.78–51.09) | -28.49 | -1.67(-1.78–-1.57) |
| Malaysia | 314.82(174.02–323.1) | 1.07 | -0.17(-0.24–-0.09) | 5.04(3.98–6.31) | -61.39 | -3.21(-3.8–-2.6) |
| Maldives | 385.38(85.88–144.15) | -4.19 | 0.06(-0.01–0.12) | 18.45(13.47–25.5) | -67.75 | -3.72(-3.97–-3.47) |
| Mali | 560.89(400.89–803.01) | 122.79 | 0.14(0.07–0.21) | 69.67(55.64–87.27) | 46.43 | -1.29(-1.46–-1.11) |
| Malta | 179.63(42.29–61.79) | -19.04 | 0.31(0.18–0.44) | 5.35(3.5–7.83) | -59.88 | -2.09(-2.44–-1.74) |
| Marshall Islands | 216(400.6–1093.95) | -24.58 | -0.22(-0.26–-0.18) | 16.2(12.24–21.5) | -49.76 | -1.79(-2.2–-1.38) |
| Mauritania | 426.41(357.58–733.13) | 16.08 | -0.32(-0.36–-0.27) | 36.1(28.86–45.03) | -30.07 | -1.74(-1.85–-1.64) |
| Mauritius | 439.42(126.99–211.25) | -37.2 | 0.3(0.17–0.43) | 14.06(10.47–18.38) | -70.68 | -2.46(-2.82–-2.1) |
| Mexico | 341.93(226.19–380.52) | -11.31 | 0.1(0.01–0.18) | 12.27(9.33–15.54) | -66.98 | -3.14(-3.22–-3.07) |
| Micronesia  (Federated States of) | 229.94(363.15–1000.78) | -49.14 | -0.34(-0.38–-0.3) | 9.93(7.13–13.33) | -80.01 | -3.74(-3.87–-3.62) |
| Monaco | 145.31(57.89–112.07) | -6.27 | -0.79(-0.84–-0.74) | 1.81(1.34–2.35) | -60.82 | -3.62(-3.69–-3.55) |
| Mongolia | 225.16(262.7–509.99) | 0.14 | -0.35(-0.41–-0.29) | 14.65(11.42–18.75) | -60.2 | -3.54(-4.06–-3.03) |
| Montenegro | 238.09(127.8–201.84) | -50.63 | -1.12(-1.26–-0.98) | 4.13(3.3–5.03) | -86.5 | -5.78(-6.41–-5.14) |
| Morocco | 319.11(150.1–326.2) | -29.63 | -0.24(-0.27–-0.2) | 17.77(12.55–23.65) | -73.32 | -3.43(-3.71–-3.15) |
| Mozambique | 392.24(561.97–1372.26) | 37.48 | -1.28(-1.44–-1.12) | 45.31(36.46–56.13) | 24.31 | -1.46(-1.6–-1.32) |
| Myanmar | 370.36(167.17–404.93) | -28.93 | -0.25(-0.34–-0.16) | 32.12(23.77–41.68) | -57.81 | -2.21(-2.45–-1.96) |
| Namibia | 448.4(316.49–722.51) | 36.73 | 0.33(0.29–0.37) | 29.43(22.31–39.88) | -17.01 | -1.23(-1.34–-1.13) |
| Nauru | 235.76(356.11–930.95) | -32.05 | -0.27(-0.32–-0.22) | 18.79(14.31–24.27) | -42.19 | -0.32(-0.96–0.32) |
| Nepal | 437.15(161.28–373.19) | -35.33 | -0.67(-0.8–-0.54) | 28.49(22.9–34.53) | -66.69 | -2.61(-2.87–-2.35) |
| Netherlands | 181.47(57.67–73.69) | -0.96 | 0.42(0.39–0.46) | 3.56(2.76–4.44) | -51.76 | -1.99(-2.11–-1.87) |
| New Zealand | 190.76(62.75–80.39) | 19.86 | 0.72(0.6–0.85) | 3.51(2.72–4.41) | -49.48 | -1.53(-1.91–-1.16) |
| Nicaragua | 374.47(359.89–626.32) | -23.06 | -0.35(-0.53–-0.18) | 9.98(7.62–12.6) | -75.26 | -4.47(-4.56–-4.39) |
| Niger | 578.33(487.57–984.2) | 176.56 | 0.2(0.07–0.33) | 41.1(34.57–48.54) | 90.85 | -1.43(-1.61–-1.24) |
| Nigeria | 403.79(239.42–520.17) | 70.2 | -0.25(-0.28–-0.22) | 54.24(43.51–68.25) | 61.45 | -0.31(-0.4–-0.22) |
| Niue | 251.59(213.73–501.51) | -51.75 | 0.02(-0.03–0.07) | 14.5(10.4–19.32) | -65.25 | -1.25(-1.54–-0.97) |
| North Macedonia | 328.63(137.26–255.65) | 6.62 | 2.31(2.12–2.51) | 10.57(8.58–12.89) | -80.84 | -3.29(-3.84–-2.74) |
| Northern Mariana Islands | 233.88(341.68–603.48) | -59.01 | 0.2(0.05–0.35) | 6.88(5.23–8.73) | -70.05 | -0.06(-0.63–0.51) |
| Norway | 153.49(73.11–90.05) | -5.41 | -0.06(-0.23–0.12) | 1.9(1.35–2.33) | -71.87 | -3.73(-3.94–-3.52) |
| Oman | 278.58(58.73–96.62) | 21.25 | 0.05(-0.03–0.12) | 9.03(7.25–10.76) | -64.65 | -3.79(-4.78–-2.79) |
| Pakistan | 504.24(107.17–210.77) | 37.48 | 0.1(0.04–0.15) | 78.62(64.03–95.2) | 19.25 | -0.24(-0.38–-0.11) |
| Palau | 258.29(641.92–1196) | -49.46 | -0.09(-0.15–-0.02) | 8.13(5.67–11.25) | -77.99 | -2.53(-2.68–-2.38) |
| Palestine | 276.92(44.73–86.02) | 30.35 | -0.06(-0.16–0.04) | 13.46(10.05–17.82) | -56.01 | -3.29(-3.62–-2.95) |
| Panama | 356.95(234.3–425.46) | 27.08 | -0.07(-0.13–-0.01) | 9.77(6.87–13.25) | -32.74 | -2.24(-2.39–-2.1) |
| Papua New Guinea | 262.48(262.37–687.26) | 110.92 | -0.19(-0.24–-0.13) | 27.92(20.14–37.86) | 94.64 | -0.28(-0.39–-0.17) |
| Paraguay | 245.05(376.32–724.12) | -30.83 | -1.52(-1.62–-1.43) | 9.02(5.92–12.78) | -68.39 | -4.12(-4.61–-3.62) |
| Peru | 456.65(250.6–507.31) | -8.9 | -0.16(-0.27–-0.06) | 12.8(8.78–17.82) | -67.67 | -3.47(-3.6–-3.33) |
| Philippines | 390.81(164.63–303.9) | 1.91 | -0.81(-0.93–-0.69) | 18.62(13.79–24.34) | -21.47 | -1.4(-1.57–-1.23) |
| Poland | 175.2(138.08–229.12) | -40.57 | -0.75(-0.93–-0.57) | 3.1(2.22–4.21) | -87.62 | -5.96(-6.26–-5.67) |
| Portugal | 192.71(83.79–110.44) | -13.4 | 0.74(0.66–0.82) | 2.54(1.91–3.32) | -85 | -4.52(-4.97–-4.07) |
| Puerto Rico | 435.79(91.04–165.92) | -53.32 | 0.95(0.76–1.15) | 8.89(6.47–11.77) | -81.59 | -2.46(-2.66–-2.25) |
| Qatar | 315.68(78.22–139.27) | 178.04 | 0.28(0.24–0.31) | 6.11(4.58–8.07) | -29.99 | -4.84(-5–-4.69) |
| Republic of Korea | 171.17(63.89–97.95) | -35.3 | 0.2(0.09–0.3) | 2.71(2.25–3.2) | -80.21 | -3.27(-3.68–-2.87) |
| Republic of Moldova | 400.59(174.7–270.94) | -59.46 | -0.35(-0.54–-0.16) | 9.15(7.06–11.87) | -79.87 | -2.59(-3.07–-2.11) |
| Romania | 238.49(249.97–466.88) | -46.56 | -0.41(-0.59–-0.24) | 5.25(4.16–6.44) | -75.42 | -3.51(-3.98–-3.05) |
| Russian Federation | 321.16(145.46–250.63) | -18.78 | -0.29(-0.34–-0.23) | 4.39(3.45–5.45) | -77.33 | -5.08(-5.43–-4.74) |
| Rwanda | 341.89(423.28–974.37) | 1.55 | -0.41(-0.6–-0.22) | 33.39(27–41.24) | -38.01 | -1.89(-2.17–-1.62) |
| Saint Kitts and Nevis | 298.86(276.56–564.04) | -19.79 | 0.14(0.12–0.16) | 17.61(13.72–22.3) | -63.83 | -2.56(-2.85–-2.27) |
| Saint Lucia | 339.58(343.36–525.68) | -48.1 | 0.46(0.37–0.54) | 18.63(12.7–26.39) | -66.25 | -0.82(-1–-0.65) |
| Saint Vincent and the Grenadines | 356.3(538.29–781.93) | -43.35 | 0.05(-0.09–0.19) | 17.4(12.18–24.19) | -70.19 | -2.22(-2.39–-2.06) |
| Samoa | 244.38(244.4–590.47) | 5.47 | -0.2(-0.25–-0.14) | 9.06(5.97–13.26) | -61.39 | -3.35(-3.44–-3.25) |
| San Marino | 160.85(40.94–108.48) | 24.75 | 0.29(0.27–0.3) | 3.3(2.53–4.21) | -51.8 | -3.07(-3.3–-2.84) |
| Sao Tome and Principe | 450.4(560.01–1102.19) | -7.92 | -0.44(-0.5–-0.38) | 18.82(13.25–25.78) | -38.12 | -1.54(-1.72–-1.35) |
| Saudi Arabia | 254.63(48.08–88.08) | -27.97 | -0.68(-0.74–-0.62) | 4.89(3.53–6.46) | -89.48 | -7.38(-7.73–-7.03) |
| Senegal | 466.29(423.11–792.84) | 25.88 | -0.12(-0.18–-0.06) | 42.32(34.59–51.35) | -5.33 | -1.19(-1.28–-1.09) |
| Serbia | 214.78(196.42–359.46) | -62.23 | -2.34(-2.58–-2.10) | 4.31(3.34–5.44) | -91.25 | -6.84(-7.45–-6.22) |
| Seychelles | 389.57(371.07–612.83) | -8.22 | 0.22(0.15–0.28) | 10.77(7.95–14.13) | -55.27 | -1.64(-1.91–-1.38) |
| Sierra Leone | 433.53(450.32–974.43) | 45.92 | -0.75(-0.81–-0.69) | 50.86(40.12–63.85) | -8.37 | -2.42(-2.49–-2.35) |
| Singapore | 232.47(62.24–85.34) | 33.49 | 1(0.76–1.24) | 1.08(0.72–1.57) | -76.67 | -4.56(-5.1–-4.02) |
| Slovakia | 197.89(110.45–225.67) | -25.44 | 0.12(0.07–0.16) | 4.22(3.09–5.54) | -77.67 | -3.94(-4.25–-3.63) |
| Slovenia | 267.32(62.74–121.35) | 14.39 | 1.21(1.11–1.31) | 1.69(1.1–2.33) | -83.11 | -5.1(-5.33–-4.87) |
| Solomon Islands | 235.51(457.59–1570.95) | 32.93 | -0.44(-0.5–-0.37) | 11.17(7.28–15.74) | -22.67 | -2.27(-2.48–-2.06) |
| Somalia | 431.96(591.13–1575.14) | 148.5 | -0.24(-0.32–-0.17) | 47.62(35.29–64.15) | 106.89 | -0.6(-0.73–-0.46) |
| South Africa | 426.04(407.83–596.02) | 9.22 | 0.18(0.13–0.22) | 34.66(26.53–45.17) | -15.49 | -0.49(-0.95–-0.04) |
| South Sudan | 422.47(328.63–932) | 32.88 | -0.2(-0.27–-0.14) | 51.88(40.95–65.46) | 12.53 | -0.58(-0.69–-0.47) |
| Spain | 183.99(49.51–79.92) | -26.58 | -0.55(-0.67–-0.44) | 2.71(2.18–3.32) | -66.69 | -3.04(-3.3–-2.78) |
| Sri Lanka | 380.2(67.8–140.67) | -6.9 | 0.57(0.49–0.66) | 7.49(4.99–10.64) | -75.42 | -4.02(-4.37–-3.67) |
| Sudan | 229.33(56.31–137.99) | 12.09 | -0.33(-0.37–-0.29) | 38.1(28.53–50.36) | -43.2 | -2.27(-2.4–-2.14) |
| Suriname | 469.84(419.2–691.2) | -3.02 | -0.01(-0.08–0.05) | 30.37(21.25–42) | -42 | -1.9(-2.06–-1.75) |
| Sweden | 107.32(68.77–84.65) | -12.58 | -0.37(-0.42–-0.33) | 2.18(1.59–2.64) | -55.58 | -2.21(-2.58–-1.83) |
| Switzerland | 176.87(50.73–66.63) | 7.54 | -0.04(-0.07–-0.01) | 3.46(2.98–3.99) | -32.1 | -1.09(-1.32–-0.86) |
| Syrian Arab Republic | 248.31(33.24–64.92) | -60.69 | -0.22(-0.41–-0.03) | 9.68(7.17–12.33) | -89.84 | -4.68(-5.85–-3.49) |
| Taiwan  (Province of China) | 196.9(100.83–187.46) | -44.94 | 0.32(0.22–0.43) | 3.9(3.16–4.83) | -35.76 | -0.26(-1.09–0.59) |
| Tajikistan | 292.81(103.44–212.47) | 3.66 | -0.8(-0.91–-0.7) | 22.3(18.04–27.23) | -8.53 | -1.18(-1.47–-0.88) |
| Thailand | 389.39(143.64–302.08) | -32.78 | 1.1(0.9–1.29) | 5.99(4.53–7.66) | -84.9 | -4.33(-4.48–-4.18) |
| Timor-Leste | 446(170.96–426.31) | -13.9 | -0.9(-0.97–-0.84) | 28.53(22.62–35.5) | -42.95 | -2.26(-2.39–-2.13) |
| Togo | 429.4(430.7–842.25) | 26.59 | -0.53(-0.57–-0.48) | 40(31.31–50.46) | -10.56 | -1.63(-1.66–-1.6) |
| Tokelau | 236.27(337.09–755.94) | -40.9 | -0.22(-0.3–-0.14) | 4.79(3.38–6.47) | -85.02 | -4.97(-5.24–-4.7) |
| Tonga | 262.67(363.43–697.2) | -13.75 | -0.19(-0.23–-0.15) | 10.39(7.25–14.21) | -53.83 | -2.23(-2.37–-2.09) |
| Trinidad and Tobago | 324.92(244.6–463.63) | -34.19 | 0.02(-0.07–0.1) | 16.87(11.85–23.55) | -64.22 | -2.66(-3.04–-2.29) |
| Tunisia | 259.01(50.45–104.04) | -31.4 | -0.08(-0.14–-0.01) | 11.07(8.35–14) | -83.34 | -4.82(-4.93–-4.71) |
| Turkey | 393.24(42.93–78.27) | -37.14 | 0.1(0.05–0.15) | 14.7(11.33–18.44) | -84.64 | -4.45(-4.56–-4.34) |
| Turkmenistan | 223.3(176.46–332.23) | -0.27 | 0.56(0.5–0.61) | 20.7(17.18–25.37) | -36.14 | -0.68(-1.1–-0.27) |
| Tuvalu | 226.39(354.32–816.44) | -29.2 | -0.26(-0.3–-0.23) | 10.74(7.53–14.93) | -84.14 | -5.41(-5.5–-5.31) |
| Uganda | 420.18(534.93–996.21) | 60.02 | -0.21(-0.36–-0.07) | 41.07(32.59–51.61) | 27.72 | -0.63(-0.79–-0.47) |
| Ukraine | 278.43(128.53–238.82) | -52.72 | -0.68(-0.78–-0.59) | 6.94(5.44–8.69) | -73.56 | -3.04(-3.35–-2.73) |
| United Arab Emirates | 231.66(117.32–230.98) | 29.29 | 0.25(0.18–0.32) | 3.03(1.87–4.63) | -74.76 | -4.9(-5.33–-4.46) |
| United Kingdom | 160.21(85.09–110.18) | -7.16 | -0.19(-0.27–-0.11) | 3.9(3.2–4.68) | -50.79 | -2.24(-2.34–-2.13) |
| United Republic of Tanzania | 393.95(469.38–980.09) | 46.55 | -0.39(-0.5–-0.29) | 40.18(31.12–51.62) | 30.83 | -0.66(-0.76–-0.56) |
| United States of America | 273.66(86.19–104.29) | -1.93 | 0.22(0.06–0.38) | 5.97(5.45–6.54) | -43.05 | -1.32(-1.45–-1.19) |
| United States Virgin Islands | 293.58(167.92–293.95) | -42.61 | 0.2(0.16–0.23) | 7.25(5.07–9.99) | -79.13 | -3.21(-3.34–-3.07) |
| Uruguay | 230.28(240.42–311.8) | -16.96 | 0.03(-0.03–0.09) | 6.71(4.8–9.23) | -74.75 | -4.36(-4.58–-4.15) |
| Uzbekistan | 214.05(219.9–339.05) | 3.8 | 0.1(0.06–0.14) | 15.31(12.15–19.48) | -40.25 | -1.78(-2.22–-1.35) |
| Vanuatu | 230.23(298.12–842.03) | 24.9 | -0.29(-0.33–-0.25) | 18.59(13.83–24.64) | -7.63 | -1.08(-1.31–-0.86) |
| Venezuela  (Bolivarian Republic of) | 421(363.38–688.29) | -9.65 | 0.53(0.43–0.64) | 14.66(10.31–20.36) | -53.26 | -1.77(-2.06–-1.48) |
| Viet Nam | 290.24(173.03–325.09) | -40.23 | -0.82(-0.89–-0.75) | 10.08(7.33–13.69) | -76.35 | -3.79(-3.9–-3.67) |
| Yemen | 612.02(72.58–172.6) | 41.23 | 0.02(-0.02–0.06) | 42.49(31.13–55.25) | -24.47 | -2.1(-2.13–-2.07) |
| Zambia | 429.06(568.8–1229.52) | 60.67 | 0.08(0.03–0.14) | 33.58(25.24–45.19) | 15.79 | -0.94(-1.17–-0.72) |
| Zimbabwe | 339.17(639–1349.6) | 6.63 | -0.35(-0.54–-0.17) | 40.53(31.4–52.38) | 19.87 | 0.41(0.24–0.57) |

EAPC: estimated annual percentage change; ASR, age-standardized rate; CI, confidence interval; UI: uncertainty interval.

**Supplementary table 2.** The percentage changes in number and the EAPCs of neonatal disorders incidence due to specific causes in global, SDI areas and geographic regions from 1990 to 2019

|  | **NPB** | | **NE** | | | **NS** | | | **HD** | |
| --- | --- | --- | --- | --- | --- | --- | --- | --- | --- | --- |
| **Characteristics** | Percentage (%) | EAPC  (95%CI) | | Percentage (%) | EAPC  (95%CI) | Percentage (%) | EAPC  (95%CI) | Percentage (%) | | EAPC  (95%CI) |
| **Overall** | -5.26 | -0.19(-0.26–-0.11) | | 3.26 | 0.07(-0.01–0.15) | 12.79 | 0.46(0.43–0.48) | 4.04 | | 0.13(0.03–0.23) |
| **SDI** |  |  | |  |  |  |  |  | |  |
| Low | 36.66 | -0.33(-0.40–-0.27) | | 39.28 | -0.15(-0.24–-0.07) | 60.36 | 0.29(0.19–0.38) | 49.22 | | -0.01(-0.07–0.04) |
| Low-middle | -22.61 | -0.66(-0.75–-0.58) | | -14.2 | -0.27(-0.33–-0.21) | 11.02 | 0.55(0.52–0.59) | -5.06 | | 0.1(0.04–0.15) |
| Middle | -22.13 | -0.11(-0.15–-0.06) | | -37.83 | -1.03(-1.13–-0.92) | -7.66 | 0.44(0.36–0.52) | -13.97 | | 0.11(0–0.22) |
| High-middle | -25.03 | -0.10(-0.13–-0.07) | | -43.83 | -1.25(-1.37–-1.13) | -9.1 | 0.78(0.72–0.84) | -47.47 | | -1.23(-1.57–-0.9) |
| High | -6.23 | 0.25(0.13–0.38) | | -19.29 | -0.31(-0.36–-0.25) | -9.17 | 0.14(0.07–0.20) | -53.58 | | -1.84(-2.3–-1.38) |
| **Regions** |  |  | |  |  |  |  |  | |  |
| East Asia | -48.1 | -0.76(-0.89–-0.63) | | -76.74 | -3.90(-4.20–-3.60) | 5.41 | 2.16(2.04–2.27) | -61.73 | | -1.46(-1.72–-1.19) |
| South Asia | -19.37 | -0.52(-0.59–-0.45) | | -17.65 | -0.50(-0.60–-0.41) | 23.06 | 0.77(0.71–0.84) | -2.48 | | 0.09(0.07–0.12) |
| Southeast Asia | -24.56 | -0.48(-0.49–-0.46) | | -54.4 | -2.48(-2.59–-2.37) | -26.66 | -0.60(-0.67–-0.52) | -17.98 | | -0.18(-0.23–-0.13) |
| Central Asia | -10.16 | -0.33(-0.37–-0.30) | | -9.26 | -0.15(-0.27–-0.02) | -16.38 | -0.60(-0.72–-0.47) | -3.12 | | 0.13(0.06–0.19) |
| High-income Asia Pacific | -22.58 | 0.46(0.37–0.56) | | -34.56 | -0.17(-0.27–-0.06) | -23.27 | -0.08(-0.2–0.04) | -29.88 | | 0.04(0.01–0.07) |
| Oceania | 80.72 | -0.09(-0.14–-0.04) | | 62.63 | -0.57(-0.6–-0.53) | 65.54 | -0.59(-0.72–-0.45) | 93.08 | | 0.16(0.14–0.18) |
| Australasia | 25.30 | 0.28(0.18–0.39) | | -12.22 | -1.50(-1.76–-1.23) | 3.94 | -0.25(-0.32–-0.18) | 19.76 | | 0.14(0.12–0.15) |
| Eastern Europe | -16.68 | 0.36(0.33–0.38) | | -50.30 | -1.63(-1.89–-1.36) | -33.54 | -0.64(-0.70–-0.58) | -76.93 | | -4.39(-5.17–-3.61) |
| Western Europe | -7.44 | -0.01(-0.04–0.01) | | -8.36 | -0.09(-0.12–-0.07) | -7.33 | -0.05(-0.11–0.01) | -7.82 | | -0.07(-0.1–-0.04) |
| Central Europe | -33.16 | 0.12(0.09–0.14) | | -36.38 | -0.17(-0.26–-0.08) | -42.38 | -0.85(-1.26–-0.44) | -79.51 | | -3.77(-4.51–-3.03) |
| High-income North America | -0.47 | 0.26(0.08–0.45) | | -12.16 | -0.09(-0.17–-0.01) | -11.38 | -0.11(-0.20–-0.01) | -9.43 | | -0.06(-0.13–0.02) |
| Andean Latin America | -1.78 | -0.49(-0.54–-0.44) | | 3.42 | -0.34(-0.50–-0.17) | -7.03 | -0.52(-0.68–-0.36) | 9.02 | | -0.02(-0.03–0) |
| Central Latin America | -3.71 | 0.30(0.26–0.34) | | -48.51 | -1.71(-1.86–-1.56) | 6.93 | 0.69(0.45–0.93) | -18.04 | | -0.26(-0.29–-0.22) |
| Caribbean | -5.15 | 0.12(0.08–0.16) | | -1.96 | 0.2(0.17–0.24) | -1.14 | 0.27(0.05–0.48) | 3.92 | | 0.44(0.41–0.48) |
| Tropical Latin America | -10.38 | 0.02(-0.01–0.04) | | -25.61 | -0.69(-0.75–-0.62) | 9.91 | 0.76(0.65–0.88) | -15.27 | | -0.09(-0.21–0.02) |
| Southern Latin America | -2.63 | 0.25(0.23–0.28) | | 5.5 | 0.67(0.51–0.84) | -37.75 | -1.71(-1.85–-1.57) | 6.46 | | 0.58(0.49–0.66) |
| Eastern Sub- Saharan Africa | 38.39 | -0.48(-0.59–-0.36) | | 35.95 | -0.54(-0.6–-0.48) | 64.03 | 0.18(0.09–0.28) | 52.04 | | -0.14(-0.16–-0.13) |
| Southern Sub- Saharan Africa | 4.06 | -0.16(-0.24–-0.09) | | 3.71 | -0.02(-0.11–0.06) | 21.35 | 0.65(0.45–0.84) | 9.43 | | 0.06(0.04–0.07) |
| Western Sub- Saharan Africa | 73.32 | -0.19(-0.22–-0.16) | | 63.04 | -0.51(-0.58–-0.44) | 78.05 | -0.23(-0.27–-0.18) | 80.24 | | -0.05(-0.06–-0.03) |
| North Africa and Middle East | 9.91 | 0.17(0.15–0.20) | | -5.4 | -0.56(-0.65–-0.46) | 22.62 | 0.45(0.39–0.51) | 0.03 | | -0.2(-0.28–-0.13) |
| Central Sub- Saharan Africa | 54.46 | -0.23(-0.28–-0.17) | | 75.59 | 0.71(0.49–0.94) | 80.75 | 0.3(0.26–0.33) | 61.08 | | -0.1(-0.13–-0.07) |

NPB, neonatal preterm birth; NE, neonatal encephalopathy due to birth asphyxia and trauma; NS, neonatal sepsis and other neonatal infections; HD, hemolytic disease and other neonatal jaundice; EAPC: estimated annual percentage change; CI, confidence interval; SDI: socio-demographic index.

**Supplementary table 3**. the percentage changes in number and the EAPCs of incidence of neonatal disorders due to etiologies at national level from 1990 to 2019

|  | **NPB** | | | **NE** | | | **NS** | | | | **HD** | | |
| --- | --- | --- | --- | --- | --- | --- | --- | --- | --- | --- | --- | --- | --- |
| **Characteristics** | Percentage (%) | EAPC  (95%CI) | Percentage (%) | | EAPC  (95%CI) | Percentage (%) | | EAPC  (95%CI) | | Percentage (%) | | EAPC  (95%CI) |  |
| Afghanistan | 164.78 | -0.12(-0.31–0.08) | 161.25 | | -0.33(-0.49–-0.16) | 241.37 | | 0.68(0.55–0.8) | 179.84 | | | 0.11(-0.03–0.25) |  |
| Albania | -67.71 | -1.07(-1.18–-0.97) | -49.57 | | 0.76(0.62–0.9) | -43.57 | | 0.93(0.66–1.2) | | -56.96 | | 0.22(0.14–0.3) |  |
| Algeria | 10.24 | -0.22(-0.26–-0.17) | 34.97 | | 0.38(0.31–0.44) | 87.04 | | 1.78(1.63–1.94) | | 14.59 | | 0.05(-0.01–0.11) |  |
| American Samoa | -42.36 | -0.25(-0.26–-0.24) | -58.32 | | -1.66(-1.76–-1.55) | -58.98 | | -1.41(-1.85–-0.97) | | -38.08 | | 0(-0.03–0.04) |  |
| Andorra | 5.98 | 0.7(0.54–0.86) | -3.49 | | 0.49(0.28–0.7) | -23.67 | | -0.73(-0.83–-0.63) | | 1.36 | | 0.54(0.39–0.69) |  |
| Angola | 91.29 | -0.4(-0.45–-0.36) | 107.86 | | -0.24(-0.33–-0.16) | 127.68 | | 0.12(0.09–0.15) | | 110.92 | | -0.11(-0.13–-0.1) |  |
| Antigua and Barbuda | -21.75 | 0.26(0.24–0.28) | -11.57 | | 0.43(0.28–0.58) | -20.16 | | 0.65(0.42–0.88) | | -25.85 | | 0.07(0.06–0.08) |  |
| Argentina | 3.71 | 0.17(0.14–0.19) | 10.29 | | 0.6(0.44–0.75) | -40.15 | | -2.19(-2.34–-2.03) | | 6.9 | | 0.3(0.26–0.33) |  |
| Armenia | -50.13 | -0.04(-0.12–0.04) | -57.11 | | -0.38(-0.51–-0.26) | -69.02 | | -2.13(-2.44–-1.83) | | -72.34 | | -1.09(-1.7–-0.49) |  |
| Australia | 26.35 | 0.2(0.11–0.3) | -12.17 | | -1.71(-2.06–-1.37) | -4.66 | | -0.85(-0.93–-0.77) | | 24.09 | | 0.15(0.13–0.17) |  |
| Austria | -1.66 | 0.11(0.09–0.12) | -3.42 | | -0.1(-0.23–0.03) | 7.59 | | 0.35(0.21–0.49) | | -5.16 | | -0.03(-0.04–-0.01) |  |
| Azerbaijan | -33.26 | -0.53(-0.66–-0.41) | -18.39 | | 0.44(0.24–0.64) | -32.44 | | -0.72(-0.84–-0.6) | | -23.08 | | 0.25(0.17–0.32) |  |
| Bahamas | -20.71 | 0.03(-0.01–0.07) | -22.74 | | -0.1(-0.16–-0.03) | -35.44 | | -0.32(-0.48–-0.16) | | -20.04 | | 0.04(-0.01–0.09) |  |
| Bahrain | 43.09 | 1.84(1.7–1.99) | -14.5 | | -0.65(-0.89–-0.41) | 31 | | 2.13(1.72–2.54) | | -5.12 | | -0.25(-0.71–0.2) |  |
| Bangladesh | -39.72 | -0.5(-0.6–-0.41) | -27.28 | | 0.47(0.21–0.73) | 22.49 | | 2.1(1.92–2.29) | | -33.52 | | -0.09(-0.11–-0.06) |  |
| Barbados | -30.27 | 0.05(0.04–0.06) | -25.82 | | 0.21(0.17–0.24) | -6.24 | | 1.51(1.31–1.71) | | -28.11 | | 0.17(0.16–0.19) |  |
| Belarus | -21.42 | 0.29(0.27–0.32) | -53.53 | | -1.79(-2.01–-1.56) | -37.15 | | -0.83(-0.97–-0.69) | | -25.22 | | 0.07(0.05–0.08) |  |
| Belgium | 0.09 | 0.02(-0.01–0.05) | 3.15 | | 0.25(0.16–0.34) | -16.71 | | -0.6(-0.9–-0.3) | | -2.58 | | -0.04(-0.05–-0.03) |  |
| Belize | 26.01 | -0.02(-0.08–0.04) | 36.97 | | 0.02(-0.05–0.1) | 36.49 | | 0.55(0.34–0.76) | | 23.26 | | -0.17(-0.2–-0.14) |  |
| Benin | 78.74 | -0.74(-0.83–-0.66) | 101.28 | | -0.22(-0.27–-0.16) | 84.02 | | -0.76(-0.83–-0.68) | | 113.36 | | 0.06(0.05–0.07) |  |
| Bermuda | -37.34 | 0.35(0.32–0.38) | -41.17 | | 0.03(-0.06–0.12) | -35.33 | | 0.77(0.62–0.92) | | -42.2 | | 0.05(0.04–0.07) |  |
| Bhutan | -53.79 | -1.07(-1.12–-1.02) | -55.64 | | -1.29(-1.43–-1.16) | 12.85 | | 2.53(2.32–2.74) | | -43.79 | | -0.35(-0.4–-0.29) |  |
| Bolivia | 18 | -0.35(-0.39–-0.3) | 14.9 | | -0.75(-0.84–-0.65) | 9.27 | | -0.67(-0.79–-0.54) | | 32.08 | | 0.02(0–0.04) |  |
| Bosnia and Herzegovina | -64.97 | -0.76(-0.91–-0.61) | -58.31 | | -0.05(-0.21–0.11) | -30.45 | | 2.49(2.24–2.73) | | -77.38 | | -3(-3.58–-2.41) |  |
| Botswana | 8.05 | -0.31(-0.37–-0.24) | 29.45 | | 0.46(0.39–0.53) | 55.08 | | 0.86(0.69–1.02) | | 18.49 | | 0.02(0.01–0.02) |  |
| Brazil | -10.34 | 0.03(0.01–0.06) | -25.62 | | -0.67(-0.74–-0.61) | 13.27 | | 0.91(0.79–1.03) | | -11.94 | | 0.07(-0.02–0.15) |  |
| Brunei Darussalam | -0.27 | 0.36(0.32–0.39) | 29.17 | | 1.34(1.23–1.44) | 50.54 | | 1.94(1.83–2.05) | | -10.16 | | -0.02(-0.09–0.05) |  |
| Bulgaria | -39 | 0.04(0.01–0.07) | -7.71 | | 1.49(1.33–1.65) | 135.38 | | 6.24(5.69–6.79) | | -96 | | -9.3(-11.57–-6.97) |  |
| Burkina Faso | 90.91 | -0.56(-0.66–-0.46) | 96.55 | | -0.26(-0.34–-0.17) | 117.85 | | 0.13(0.08–0.17) | | 104.59 | | -0.03(-0.04–-0.01) |  |
| Burundi | 48.73 | -0.76(-0.87–-0.66) | 64.9 | | -0.38(-0.47–-0.3) | 96 | | 0.62(0.42–0.82) | | 75.53 | | -0.05(-0.12–0.01) |  |
| Cabo Verde | -20.86 | -0.55(-0.58–-0.52) | -7.42 | | 0.06(-0.03–0.15) | 55.85 | | 2.15(2.04–2.26) | | -11.14 | | -0.1(-0.15–-0.05) |  |
| Cambodia | -37.99 | -1.47(-1.66–-1.29) | -45.79 | | -1.75(-1.86–-1.65) | -6.38 | | 0.47(0.34–0.6) | | -21.82 | | -0.26(-0.29–-0.23) |  |
| Cameroon | 89.15 | -0.07(-0.14–0) | 83.83 | | -0.25(-0.31–-0.18) | 95.06 | | -0.09(-0.24–0.07) | | 88.75 | | -0.03(-0.07–0.01) |  |
| Canada | -3.14 | 0.17(0.15–0.18) | -3.39 | | 0.33(0.22–0.44) | 8.84 | | 0.6(0.49–0.72) | | -8.48 | | -0.06(-0.09–-0.04) |  |
| Central African  Republic | 48.22 | -0.2(-0.25–-0.15) | 69.93 | | 0.4(0.34–0.46) | 41.96 | | -0.12(-0.21–-0.03) | | 54.87 | | 0.04(0.01–0.08) |  |
| Chad | 142.7 | 0.01(-0.05–0.07) | 139.62 | | -0.25(-0.34–-0.17) | 112.84 | | -0.78(-0.96–-0.61) | | 152.24 | | 0.06(0.06–0.07) |  |
| Chile | -11.88 | 0.69(0.61–0.78) | -0.01 | | 1.07(0.72–1.41) | -22.68 | | -0.01(-0.13–0.11) | | -20.9 | | 0.09(0.03–0.15) |  |
| China | -47.92 | -0.78(-0.92–-0.65) | -76.84 | | -3.96(-4.27–-3.66) | 5.87 | | 2.12(2.01–2.24) | | -62.02 | | -1.5(-1.78–-1.23) |  |
| Colombia | 31.71 | 1.57(1.43–1.72) | -58.61 | | -2.5(-2.69–-2.31) | 64.22 | | 2.43(2.06–2.8) | | -12.66 | | 0.08(0.06–0.1) |  |
| Comoros | -20.72 | -0.14(-0.17–-0.1) | -25.56 | | -0.47(-0.56–-0.37) | -12.59 | | 0.35(0.21–0.49) | | -22.93 | | -0.31(-0.32–-0.3) |  |
| Congo | 34.01 | -0.21(-0.25–-0.18) | 61.39 | | 0.41(0.34–0.48) | 61.41 | | 0.68(0.54–0.82) | | 38.85 | | -0.08(-0.1–-0.05) |  |
| Cook Islands | -45.53 | -0.14(-0.19–-0.09) | -63.60 | | -1.87(-1.96–-1.77) | -41.85 | | -0.02(-0.12–0.09) | | -41.47 | | 0.07(0.04–0.1) |  |
| Costa Rica | -17.23 | 0.05(0.03–0.07) | -22.25 | | 0.03(-0.13–0.18) | -65.94 | | -3.11(-3.41–-2.81) | | -50.02 | | -0.97(-1.48–-0.45) |  |
| Croatia | -30.06 | 0.35(0.31–0.38) | -0.03 | | 1.65(1.55–1.75) | 10.53 | | 1.08(0.05–2.13) | | -36.2 | | 0.14(0.08–0.19) |  |
| Cuba | -59.26 | -1.47(-1.71–-1.23) | -49.55 | | -0.68(-0.75–-0.61) | -50.31 | | -0.23(-0.42–-0.03) | | -43.47 | | -0.11(-0.14–-0.08) |  |
| Cyprus | 13.23 | 0.15(0.11–0.19) | -7.15 | | -0.23(-0.4–-0.06) | -3.55 | | -0.62(-0.68–-0.56) | | 5.3 | | -0.07(-0.24–0.1) |  |
| Czechia | 4.4 | 1.05(0.88–1.23) | 24.75 | | 1.21(1.11–1.32) | 66.2 | | 2.49(1.67–3.33) | | -12.9 | | 0.01(-0.03–0.05) |  |
| Côte d'Ivoire | 42.74 | -0.13(-0.23–-0.03) | 46.36 | | -0.23(-0.29–-0.16) | 32.27 | | -0.74(-0.82–-0.65) | | 51.32 | | -0.08(-0.09–-0.08) |  |
| Democratic People's Republic of Korea | -53.82 | -0.21(-0.28–-0.15) | -75.78 | | -2.61(-2.79–-2.43) | -50.95 | | 0(-0.32–0.33) | | -56.21 | | -0.38(-0.42–-0.33) |  |
| Democratic Republic of the Congo | 45.56 | -0.18(-0.28–-0.09) | 67.32 | | 1.06(0.72–1.4) | 69.97 | | 0.3(0.25–0.34) | | 50.4 | | -0.11(-0.14–-0.07) |  |
| Denmark | 3.76 | 0.19(0.16–0.21) | 24.85 | | 0.82(0.63–1.01) | 0.8 | | 0.03(-0.02–0.08) | | -1.24 | | -0.03(-0.05–-0.01) |  |
| Djibouti | 49.37 | -0.3(-0.37–-0.24) | 56.5 | | -0.19(-0.29–-0.1) | 54.89 | | 0.17(0.05–0.28) | | 49.69 | | -0.31(-0.39–-0.23) |  |
| Dominica | -53.53 | -0.18(-0.22–-0.15) | -6.03 | | 2.4(2.34–2.47) | -37.91 | | 1.31(1.13–1.48) | | -51.56 | | -0.08(-0.12–-0.04) |  |
| Dominican Republic | 3.99 | 0.39(0.34–0.44) | -22.35 | | -0.77(-0.81–-0.73) | 14.16 | | 0.41(0.1–0.73) | | -5.09 | | -0.05(-0.07–-0.03) |  |
| Ecuador | 12.04 | -0.12(-0.23–0) | 40.66 | | 0.32(-0.11–0.75) | -22.78 | | -2.11(-2.63–-1.59) | | 16.7 | | -0.04(-0.06–-0.02) |  |
| Egypt | 16.35 | 0.2(0.12–0.28) | -8.27 | | -0.94(-1.04–-0.85) | 51.79 | | 0.73(0.57–0.89) | | 5.01 | | -0.16(-0.2–-0.12) |  |
| El Salvador | -34.31 | -0.21(-0.25–-0.17) | -65.77 | | -2.33(-2.54–-2.13) | -51.58 | | -1.5(-1.82–-1.17) | | -63.68 | | -1.34(-1.96–-0.72) |  |
| Equatorial Guinea | 23.49 | -1.25(-1.4–-1.1) | 78.75 | | 0.25(0.17–0.34) | 134.64 | | 1.57(1.34–1.79) | | 51.37 | | -0.41(-0.43–-0.4) |  |
| Eritrea | 25.61 | -0.48(-0.59–-0.37) | 44.21 | | -0.16(-0.28–-0.04) | 80.64 | | 0.24(0.18–0.29) | | 39.67 | | -0.21(-0.29–-0.12) |  |
| Estonia | -33.97 | 0.17(0.1–0.25) | -49.92 | | -0.57(-0.73–-0.4) | -69.46 | | -2.97(-3.72–-2.22) | | -36.25 | | 0.04(-0.02–0.1) |  |
| Eswatini | -19.22 | -0.46(-0.49–-0.44) | -4.08 | | 0.28(0.17–0.39) | 0.83 | | 0.26(0.11–0.41) | | -10.49 | | -0.09(-0.11–-0.06) |  |
| Ethiopia | 37.1 | -0.24(-0.44–-0.04) | 10.66 | | -1.06(-1.14–-0.98) | 67.14 | | 0.54(0.43–0.64) | | 43.23 | | -0.15(-0.18–-0.12) |  |
| Fiji | -10.66 | -0.12(-0.14–-0.11) | -13.89 | | -0.45(-0.55–-0.34) | -27.21 | | -1.19(-1.43–-0.94) | | -3.27 | | 0.16(0.14–0.17) |  |
| Finland | -11.58 | 0.48(0.41–0.55) | -20.51 | | 0.12(0.08–0.16) | -30.51 | | -0.37(-0.44–-0.31) | | -23.36 | | 0(-0.01–0.01) |  |
| France | 0.22 | 0.23(0.22–0.25) | -5.49 | | 0.02(-0.04–0.09) | 3.36 | | 0.31(0.12–0.51) | | -6.35 | | 0.01(-0.01–0.03) |  |
| Gabon | -10.33 | -0.79(-0.95–-0.63) | 22.89 | | 0.47(0.39–0.55) | 42.82 | | 1.05(0.96–1.14) | | 0.8 | | -0.26(-0.27–-0.25) |  |
| Gambia | 32.62 | -0.4(-0.41–-0.38) | 22.92 | | -0.8(-0.85–-0.74) | 20.76 | | -1.04(-1.14–-0.95) | | 36.28 | | -0.35(-0.39–-0.31) |  |
| Georgia | -31.57 | 0.64(0.47–0.82) | -68.81 | | -2.18(-2.43–-1.93) | -50.92 | | -0.94(-1.58–-0.29) | | -36.34 | | 0.69(0.64–0.75) |  |
| Germany | -12.31 | -0.05(-0.12–0.01) | 5.51 | | 0.71(0.65–0.77) | -26.95 | | -0.84(-0.93–-0.75) | | -15.46 | | -0.17(-0.21–-0.12) |  |
| Ghana | 4.3 | -1.28(-1.37–-1.18) | 24.39 | | -0.47(-0.51–-0.43) | 74.76 | | 0.63(0.55–0.7) | | 35.49 | | -0.08(-0.11–-0.06) |  |
| Greece | 108.26 | 3.91(3.66–4.17) | -18.09 | | -0.12(-0.21–-0.03) | -51.18 | | -1.4(-1.83–-0.98) | | -10.6 | | 0.43(0.34–0.51) |  |
| Greenland | -35.08 | 0(-0.1–0.11) | -61.76 | | -1.95(-2.07–-1.83) | -33.51 | | -0.03(-0.14–0.08) | | -37.94 | | -0.16(-0.27–-0.05) |  |
| Grenada | -27.55 | 0.12(0.1–0.13) | -19.72 | | 0.33(0.26–0.4) | -13.41 | | 0.75(0.69–0.81) | | -28.61 | | 0.1(0.07–0.12) |  |
| Guam | -15.99 | -0.17(-0.19–-0.14) | -6.81 | | 0.29(0.17–0.42) | -14.34 | | -0.12(-0.77–0.53) | | -13.34 | | -0.02(-0.05–0.02) |  |
| Guatemala | 22.72 | 0.15(0.03–0.26) | -1.6 | | -0.38(-0.56–-0.2) | 22.82 | | 0.1(-0.11–0.31) | | 10.44 | | -0.38(-0.45–-0.31) |  |
| Guinea | 41.54 | -0.28(-0.35–-0.21) | 20.09 | | -1.01(-1.09–-0.94) | 34.24 | | -0.74(-0.83–-0.65) | | 51.63 | | -0.1(-0.14–-0.06) |  |
| Guinea-Bissau | 23.36 | -0.25(-0.26–-0.24) | 14.4 | | -0.62(-0.67–-0.57) | 16.44 | | -0.67(-0.75–-0.59) | | 30.45 | | -0.11(-0.13–-0.09) |  |
| Guyana | -41.16 | -0.32(-0.36–-0.28) | -51.09 | | -0.87(-0.92–-0.82) | -41.8 | | -0.46(-0.63–-0.29) | | -35.67 | | 0.04(-0.01–0.08) |  |
| Haiti | 23.29 | -0.41(-0.46–-0.37) | 37.67 | | 0.04(0.02–0.07) | 51.83 | | 0.62(0.52–0.71) | | 29.89 | | -0.13(-0.14–-0.12) |  |
| Honduras | 11.26 | -0.75(-0.87–-0.63) | -2.05 | | -1.02(-1.24–-0.8) | 30.43 | | -0.06(-0.23–0.12) | | 21.15 | | -0.32(-0.38–-0.27) |  |
| Hungary | -30.09 | 0.17(0.14–0.19) | -53.34 | | -1.49(-1.92–-1.06) | -22.31 | | 0.72(0.42–1.02) | | -32.41 | | -0.03(-0.07–0.01) |  |
| Iceland | -2.48 | 0.25(0.22–0.29) | -5.14 | | 0.7(0.51–0.89) | -29.58 | | -1.17(-1.26–-1.07) | | -7.23 | | 0.08(0.05–0.11) |  |
| India | -24.28 | -0.61(-0.7–-0.52) | -39.03 | | -1.42(-1.53–-1.31) | 9.58 | | 0.23(0.07–0.4) | | -13.18 | | -0.17(-0.19–-0.15) |  |
| Indonesia | -31.17 | -0.51(-0.58–-0.45) | -65.21 | | -3.17(-3.3–-3.04) | -46.58 | | -1.63(-1.73–-1.54) | | -20.14 | | -0.01(-0.03–0.01) |  |
| Iran  (Islamic Republic of) | -23.43 | -0.1(-0.15–-0.05) | -32.94 | | -0.64(-0.8–-0.48) | 12.46 | | 1.36(1.2–1.52) | | -20.65 | | 0.08(0.02–0.13) |  |
| Iraq | 22.68 | -0.13(-0.24–-0.01) | 11.96 | | -0.74(-0.87–-0.6) | 90.61 | | 1.51(1.23–1.78) | | 21.01 | | -0.3(-0.37–-0.24) |  |
| Ireland | 42.65 | 0.9(0.68–1.11) | 22.91 | | -0.39(-0.73–-0.05) | 35.53 | | 0.74(0.69–0.8) | | 18.43 | | 0.16(0.14–0.18) |  |
| Israel | 99.07 | 0.23(0.22–0.25) | 86.45 | | -0.01(-0.09–0.08) | 43.13 | | -1.26(-1.4–-1.13) | | 93.26 | | 0.1(0.07–0.13) |  |
| Italy | -19.56 | -0.06(-0.16–0.03) | -42.92 | | -1.15(-1.36–-0.94) | -9.29 | | 0.48(0.42–0.54) | | -20.65 | | -0.17(-0.28–-0.05) |  |
| Jamaica | -37.71 | -0.03(-0.08–0.01) | -13.81 | | 0.55(0.28–0.81) | -4.05 | | 1.92(1.61–2.22) | | -34.64 | | 0.07(0.06–0.08) |  |
| Japan | -17.1 | 0.54(0.43–0.65) | -31.63 | | -0.14(-0.32–0.03) | -30.37 | | -0.18(-0.22–-0.14) | | -24.89 | | 0.09(0.07–0.1) |  |
| Jordan | 94.59 | 0.31(0.21–0.41) | 117.09 | | 0.47(0.36–0.58) | 171.95 | | 1.5(1.42–1.58) | | 70.18 | | -0.25(-0.34–-0.17) |  |
| Kazakhstan | -17.36 | -0.88(-0.97–-0.78) | 9.89 | | 0.51(0.39–0.62) | -16.45 | | -0.5(-0.63–-0.37) | | 6.39 | | 0.33(0.28–0.38) |  |
| Kenya | 28.96 | -0.15(-0.26–-0.03) | 26.24 | | -0.19(-0.26–-0.13) | 10.98 | | -0.84(-0.95–-0.72) | | 24.9 | | -0.26(-0.29–-0.23) |  |
| Kiribati | 12.68 | -0.05(-0.08–-0.01) | -24.4 | | -1.57(-1.61–-1.53) | 11.37 | | -0.21(-0.35–-0.08) | | 18.44 | | 0.04(0.03–0.05) |  |
| Kuwait | 61.85 | 0.18(0.11–0.25) | 30.1 | | -0.53(-0.69–-0.38) | 11.42 | | -1.04(-1.31–-0.76) | | -74.26 | | -2.85(-4.05–-1.64) |  |
| Kyrgyzstan | 18.06 | 0.13(0.1–0.17) | -2.78 | | -0.36(-0.44–-0.27) | 0.67 | | -0.94(-1.15–-0.73) | | 20.54 | | 0.33(0.25–0.42) |  |
| Lao People's Democratic Republic | -8.67 | -0.26(-0.43–-0.08) | -50.16 | | -2.8(-2.95–-2.64) | -5.03 | | -0.32(-0.36–-0.27) | | 0.02 | | -0.13(-0.16–-0.11) |  |
| Latvia | -45.47 | 0.17(0.12–0.23) | -57.36 | | -0.46(-0.6–-0.32) | -58.69 | | -0.21(-0.42–0) | | -98.12 | | -14.43(-16.64–-12.16) |  |
| Lebanon | 2.66 | 0.2(0.09–0.31) | 1.98 | | -0.09(-0.24–0.05) | 66.68 | | 2.13(2.01–2.24) | | -94.08 | | -6.28(-8.73–-3.76) |  |
| Lesotho | -37.32 | -0.73(-0.76–-0.71) | -25.66 | | -0.04(-0.1–0.01) | -22.75 | | -0.07(-0.15–0.01) | | -28.01 | | -0.22(-0.25–-0.2) |  |
| Liberia | 47.4 | -0.26(-0.38–-0.13) | 18.35 | | -1.02(-1.15–-0.89) | 42.32 | | -0.75(-0.95–-0.55) | | 42.95 | | -0.26(-0.37–-0.14) |  |
| Libya | -48.11 | -0.08(-0.19–0.02) | -50.19 | | -0.47(-0.56–-0.37) | -43.31 | | 0.37(0.17–0.56) | | -98.22 | | -10.31(-13.43–-7.08) |  |
| Lithuania | -51.24 | 0.09(0.04–0.15) | -67.26 | | -1.25(-1.46–-1.03) | 12.38 | | 3.82(3.13–4.51) | | -51.12 | | 0.07(0.02–0.12) |  |
| Luxembourg | 42.22 | 0.21(0.17–0.25) | 27.28 | | 0.08(-0.06–0.22) | 25.97 | | -0.22(-0.25–-0.19) | | 33.79 | | -0.02(-0.04–0.01) |  |
| Madagascar | 68.21 | 0.28(0.19–0.38) | 47.76 | | -0.24(-0.31–-0.18) | 51.6 | | -0.23(-0.37–-0.1) | | 49.5 | | -0.2(-0.26–-0.14) |  |
| Malawi | 5.01 | -0.69(-0.83–-0.54) | -0.1 | | -0.69(-0.78–-0.59) | 8.9 | | -0.09(-0.22–0.03) | | 8.86 | | -0.44(-0.5–-0.37) |  |
| Malaysia | 12.86 | 0.16(0.13–0.19) | -22.27 | | -1.11(-1.61–-0.61) | -12.24 | | -0.59(-0.73–-0.46) | | -4.97 | | -0.49(-0.66–-0.32) |  |
| Maldives | -8.21 | -0.22(-0.32–-0.12) | -51.26 | | -2.67(-2.85–-2.5) | 9.66 | | 0.73(0.53–0.93) | | -75.53 | | -2.29(-3.13–-1.44) |  |
| Mali | 120.12 | 0.16(0.09–0.24) | 89.78 | | -0.46(-0.56–-0.37) | 154.7 | | 0.4(0.26–0.54) | | 112.59 | | -0.08(-0.1–-0.06) |  |
| Malta | -19.69 | 0.28(0.15–0.42) | 1.33 | | 1.14(1.03–1.24) | -19.35 | | 0.28(0.19–0.37) | | -22.09 | | 0.06(0.04–0.09) |  |
| Marshall Islands | -20.99 | -0.03(-0.08–0.02) | -42.42 | | -1.44(-1.61–-1.26) | -36.27 | | -0.93(-1.01–-0.84) | | -17.64 | | 0.08(0.07–0.09) |  |
| Mauritania | 6.2 | -0.56(-0.61–-0.52) | 11.2 | | -0.41(-0.45–-0.36) | 47.74 | | 0.33(0.22–0.44) | | 15.53 | | -0.2(-0.21–-0.19) |  |
| Mauritius | -40.26 | 0.14(0.13–0.16) | -57.85 | | -1.35(-1.56–-1.14) | -34.03 | | 0.46(0.22–0.7) | | -40.57 | | 0.11(0.09–0.13) |  |
| Mexico | -18.13 | -0.16(-0.26–-0.07) | -52.73 | | -1.79(-1.96–-1.63) | 0.82 | | 0.46(0.22–0.7) | | -26.55 | | -0.4(-0.47–-0.33) |  |
| Micronesia  (Federated States of) | -48.2 | -0.23(-0.27–-0.19) | -71.11 | | -2.63(-2.76–-2.5) | -48.85 | | -0.45(-0.58–-0.32) | | -45.83 | | -0.11(-0.15–-0.07) |  |
| Monaco | -7.66 | -0.84(-0.89–-0.79) | -9.43 | | -0.89(-0.93–-0.84) | 10.11 | | -0.32(-0.36–-0.28) | | -11.1 | | -0.99(-1.05–-0.93) |  |
| Mongolia | 8.35 | -0.1(-0.15–-0.05) | -29.37 | | -1.54(-1.65–-1.44) | -6.39 | | -0.57(-0.74–-0.41) | | 13.21 | | 0.26(0.19–0.32) |  |
| Montenegro | -30.96 | 0.08(0.07–0.1) | -47.35 | | -0.67(-0.88–-0.47) | -56.27 | | -1.46(-1.6–-1.32) | | -97.88 | | -11.47(-14.39–-8.45) |  |
| Morocco | -31.88 | -0.32(-0.36–-0.27) | -25.97 | | -0.32(-0.4–-0.24) | -3.33 | | 0.73(0.59–0.87) | | -26.38 | | -0.13(-0.15–-0.11) |  |
| Mozambique | 17.67 | -1.82(-2.04–-1.59) | 51.4 | | -0.77(-0.89–-0.66) | 76.96 | | -0.44(-0.56–-0.32) | | 85.02 | | -0.03(-0.1–0.03) |  |
| Myanmar | -29.24 | -0.19(-0.28–-0.09) | -53.7 | | -2.01(-2.13–-1.88) | -26.68 | | -0.24(-0.37–-0.11) | | -22.54 | | 0.11(0.02–0.21) |  |
| Namibia | 27.53 | 0.07(0.03–0.1) | 29.3 | | 0.17(0.09–0.25) | 89.66 | | 1.59(1.48–1.7) | | 29.3 | | 0.04(0.03–0.06) |  |
| Nauru | -31.52 | -0.2(-0.24–-0.15) | -36.59 | | -0.49(-0.73–-0.25) | -33.46 | | -0.54(-0.83–-0.25) | | -28.94 | | -0.05(-0.08–-0.02) |  |
| Nepal | -40.49 | -1.08(-1.28–-0.89) | -52.87 | | -1.84(-2.02–-1.67) | -5.28 | | 1.19(1.01–1.37) | | -32.56 | | -0.48(-0.52–-0.43) |  |
| Netherlands | -3.81 | 0.29(0.27–0.31) | -7.76 | | 0.29(0.2–0.37) | 17.13 | | 1.17(1.04–1.29) | | -9.71 | | 0.01(0–0.02) |  |
| New Zealand | 20.35 | 0.67(0.51–0.83) | -12.37 | | -0.62(-0.69–-0.54) | 33.09 | | 1.69(1.49–1.9) | | 0.92 | | 0.08(0.05–0.11) |  |
| Nicaragua | -24.28 | -0.65(-0.88–-0.41) | -63.04 | | -3.09(-3.34–-2.85) | -20.54 | | 0.04(-0.22–0.31) | | -13.97 | | -0.18(-0.23–-0.13) |  |
| Niger | 182.1 | 0.27(0.1–0.45) | 121.15 | | -0.69(-0.74–-0.63) | 184.54 | | 0.34(0.28–0.39) | | 163.88 | | 0.05(0.04–0.06) |  |
| Nigeria | 69.97 | -0.21(-0.24–-0.17) | 63.16 | | -0.52(-0.61–-0.42) | 74.31 | | -0.24(-0.33–-0.15) | | 76.97 | | -0.13(-0.14–-0.11) |  |
| Niue | -52.94 | -0.12(-0.17–-0.06) | -56.3 | | -0.49(-0.59–-0.39) | -47.76 | | 0.45(0.29–0.6) | | -52.53 | | -0.08(-0.15–-0.01) |  |
| North Macedonia | -35.79 | 0.04(-0.05–0.13) | -25.82 | | 0.57(0.4–0.75) | 300.45 | | 8.41(7.72–9.1) | | -33.93 | | 0.13(0.04–0.23) |  |
| Northern Mariana Islands | -59.66 | 0.15(0.06–0.25) | -56.79 | | 0.31(0.21–0.41) | -55.33 | | 0.44(-0.06–0.94) | | -59.5 | | 0.19(0.13–0.26) |  |
| Norway | -4.49 | -0.03(-0.23–0.18) | -20.56 | | -0.6(-0.75–-0.45) | -6.4 | | -0.07(-0.1–-0.05) | | -5.99 | | -0.06(-0.09–-0.04) |  |
| Oman | 17.93 | -0.01(-0.06–0.04) | 38.22 | | 0.34(0.22–0.47) | 89.72 | | 1.45(1.37–1.52) | | -43.87 | | -2.54(-3.42–-1.65) |  |
| Pakistan | 28.49 | -0.14(-0.21–-0.07) | 40.44 | | 0.03(-0.04–0.1) | 74.98 | | 1.1(0.98–1.21) | | 30.03 | | -0.24(-0.26–-0.22) |  |
| Palau | -49.03 | -0.03(-0.06–0.01) | -60.64 | | -0.98(-1.09–-0.87) | -49.77 | | -0.19(-0.37–-0.01) | | -47.81 | | 0.06(0.03–0.09) |  |
| Palestine | 25.96 | -0.14(-0.2–-0.07) | 5.12 | | -1.03(-1.13–-0.93) | 53.39 | | 0.44(0.19–0.69) | | 30.11 | | -0.03(-0.04–-0.02) |  |
| Panama | 26.89 | -0.05(-0.09–-0.01) | 10.93 | | -0.35(-0.49–-0.21) | 27.08 | | -0.1(-0.27–0.07) | | 35.44 | | 0.15(0.13–0.18) |  |
| Papua New Guinea | 112.24 | -0.13(-0.19–-0.08) | 93.81 | | -0.55(-0.58–-0.53) | 107.39 | | -0.39(-0.5–-0.27) | | 124.62 | | 0.08(0.06–0.1) |  |
| Paraguay | -11.62 | -0.44(-0.55–-0.33) | -25.06 | | -1.08(-1.19–-0.97) | -59.67 | | -3.88(-4.25–-3.51) | | -48.87 | | -2.24(-2.89–-1.59) |  |
| Peru | -11.89 | -0.56(-0.69–-0.43) | -12.73 | | -0.43(-0.54–-0.33) | -6.28 | | 0.21(0.07–0.35) | | -4.22 | | -0.07(-0.11–-0.03) |  |
| Philippines | 8.06 | -0.53(-0.6–-0.46) | -14.49 | | -1.59(-1.67–-1.52) | -3.85 | | -1.08(-1.27–-0.89) | | 26.98 | | 0(-0.02–0.02) |  |
| Poland | -27.58 | 0.21(0.18–0.23) | -50.29 | | -1.24(-1.36–-1.13) | -74.77 | | -4.47(-5.36–-3.58) | | -30.64 | | -0.05(-0.1–0) |  |
| Portugal | -7.3 | 1.03(0.95–1.11) | -36.55 | | -0.44(-0.67–-0.21) | -41.26 | | -0.97(-1.1–-0.85) | | -28.71 | | 0.06(0.04–0.08) |  |
| Puerto Rico | -58.42 | 0.32(0.29–0.35) | -52.57 | | 0.61(0.51–0.71) | -45.28 | | 1.77(1.33–2.21) | | -60.95 | | 0.07(0.04–0.09) |  |
| Qatar | 176.95 | 0.27(0.24–0.31) | 183.47 | | 0.02(-0.07–0.11) | 199.61 | | 0.63(0.5–0.75) | | 156.55 | | -0.05(-0.08–-0.02) |  |
| Republic of Korea | -36.11 | 0.27(0.18–0.36) | -45.33 | | -0.33(-0.47–-0.18) | -25.31 | | 0.06(-0.26–0.38) | | -41.38 | | -0.04(-0.12–0.04) |  |
| Republic of Moldova | -57.63 | 0.02(0–0.05) | -74.01 | | -1.85(-2.12–-1.58) | -59.75 | | -0.55(-0.87–-0.22) | | -56.64 | | 0.19(0.15–0.24) |  |
| Romania | -35.76 | 0.24(0.16–0.32) | -31.18 | | 0.23(0.07–0.38) | -54.21 | | -1.12(-1.39–-0.84) | | -97.89 | | -7.72(-10.51–-4.84) |  |
| Russian Federation | -9.96 | 0.18(0.16–0.21) | -48.65 | | -1.93(-2.23–-1.63) | -17.05 | | -0.32(-0.44–-0.19) | | -81.35 | | -5.2(-6.12–-4.26) |  |
| Rwanda | -14.71 | -1.22(-1.49–-0.96) | -3.23 | | -0.68(-0.93–-0.43) | 37.73 | | 1.1(0.94–1.27) | | 3.55 | | -0.37(-0.6–-0.14) |  |
| Saint Kitts and Nevis | -18.48 | 0.2(0.19–0.21) | -42.98 | | -1.28(-1.38–-1.17) | -17.38 | | 0.29(0.2–0.38) | | -21.84 | | 0.05(0.05–0.06) |  |
| Saint Lucia | -52.86 | 0.02(0.01–0.04) | -45.86 | | 0.48(0.34–0.61) | -31.74 | | 1.65(1.31–1.99) | | -53.87 | | -0.06(-0.1–-0.02) |  |
| Saint Vincent and the Grenadines | -44.38 | -0.08(-0.11–-0.05) | -48.75 | | -0.49(-0.54–-0.44) | -41.38 | | 0.28(-0.14–0.7) | | -41.61 | | 0.07(0.05–0.09) |  |
| Samoa | 10.15 | 0.02(-0.01–0.05) | -30.32 | | -1.83(-1.99–-1.67) | -7.49 | | -0.9(-1.11–-0.7) | | 14.34 | | 0.11(0.09–0.14) |  |
| San Marino | 26.55 | 0.35(0.34–0.37) | 30.23 | | 0.38(0.35–0.41) | 7.32 | | -0.38(-0.44–-0.32) | | 19.51 | | 0.14(0.13–0.14) |  |
| Sao Tome and Principe | -16.32 | -0.76(-0.81–-0.71) | -0.59 | | -0.17(-0.22–-0.12) | 22.85 | | 0.52(0.4–0.65) | | -6.08 | | -0.3(-0.33–-0.28) |  |
| Saudi Arabia | -14.38 | 0.16(0.05–0.27) | -54.74 | | -2.52(-2.68–-2.36) | -34.23 | | -1.4(-1.59–-1.2) | | -94.06 | | -6.07(-8.38–-3.71) |  |
| Senegal | 34.78 | 0.28(0.17–0.4) | 17.16 | | -0.57(-0.6–-0.53) | 1.56 | | -1.35(-1.45–-1.24) | | 30.63 | | -0.08(-0.11–-0.05) |  |
| Serbia | -44.21 | -0.5(-0.6–-0.4) | -57.46 | | -1.42(-1.56–-1.28) | -78.64 | | -4.76(-5.26–-4.26) | | -41.31 | | -0.33(-0.41–-0.25) |  |
| Seychelles | -9.6 | 0.13(0.11–0.15) | -21.28 | | -0.36(-0.48–-0.25) | -6.5 | | 0.31(0.19–0.44) | | -9.24 | | 0.13(0.11–0.16) |  |
| Sierra Leone | 46.9 | -0.66(-0.73–-0.59) | 30.78 | | -1.15(-1.26–-1.04) | 48.92 | | -0.87(-0.99–-0.75) | | 67.39 | | -0.16(-0.22–-0.09) |  |
| Singapore | 23.7 | 0.75(0.44–1.05) | 45.53 | | 1.45(1.11–1.78) | 137.25 | | 3.06(2.6–3.53) | | 4.71 | | -0.07(-0.22–0.07) |  |
| Slovakia | -26.09 | 0.18(0.16–0.2) | 2.99 | | 1.35(1.28–1.42) | -27.47 | | -0.36(-0.63–-0.09) | | -28.47 | | -0.03(-0.07–0) |  |
| Slovenia | -8.33 | 0.3(0.27–0.33) | 8.88 | | 1.19(1.01–1.36) | 71.81 | | 2.84(2.6–3.09) | | -13.01 | | -0.02(-0.06–0.02) |  |
| Solomon Islands | 36.19 | -0.29(-0.35–-0.24) | -6.19 | | -1.82(-1.92–-1.72) | 24.22 | | -0.98(-1.21–-0.75) | | 42.78 | | -0.08(-0.13–-0.03) |  |
| Somalia | 161.97 | -0.02(-0.1–0.05) | 124.17 | | -0.61(-0.73–-0.5) | 121.63 | | -0.75(-0.84–-0.66) | | 161.43 | | -0.01(-0.08–0.07) |  |
| South Africa | 4.94 | -0.06(-0.1–-0.03) | -2.93 | | -0.2(-0.29–-0.12) | 28.96 | | 1.12(0.86–1.37) | | 9.81 | | 0.13(0.12–0.15) |  |
| South Sudan | 31.54 | -0.28(-0.37–-0.18) | 48.19 | | 0.27(0.13–0.4) | 28.94 | | -0.24(-0.32–-0.17) | | 47.28 | | 0.22(0.13–0.31) |  |
| Spain | -30.34 | -0.7(-0.85–-0.55) | -9.15 | | -0.19(-0.25–-0.13) | 4.16 | | 0.35(-0.05–0.76) | | -8.75 | | 0(-0.03–0.02) |  |
| Sri Lanka | -15.77 | 0.08(0.05–0.11) | -55.39 | | -2.28(-2.38–-2.19) | 8.2 | | 1.28(1.11–1.45) | | -18.12 | | -0.03(-0.04–-0.02) |  |
| Sudan | 9.95 | -0.35(-0.4–-0.29) | 2.08 | | -0.86(-0.95–-0.76) | 48.85 | | 0.52(0.33–0.71) | | 5.7 | | -0.51(-0.54–-0.47) |  |
| Suriname | -8.26 | -0.28(-0.33–-0.23) | -9.56 | | -0.47(-0.59–-0.35) | 7.08 | | 0.48(0.36–0.6) | | -2.06 | | -0.03(-0.06–-0.01) |  |
| Sweden | -11.22 | -0.25(-0.29–-0.2) | -21.4 | | -0.74(-0.89–-0.58) | -18.04 | | -0.99(-1.16–-0.82) | | -5.36 | | -0.05(-0.06–-0.04) |  |
| Switzerland | 8.82 | 0.03(0–0.05) | 59.27 | | 1.4(1.2–1.6) | -11.41 | | -0.98(-1.08–-0.87) | | 4.24 | | -0.12(-0.14–-0.1) |  |
| Syrian Arab Republic | -58.81 | -0.1(-0.28–0.09) | -61.99 | | -0.66(-0.99–-0.34) | -56.54 | | -0.08(-0.17–0.02) | | -97.03 | | -3.4(-5.16–-1.61) |  |
| Taiwan  (Province of China) | -47.18 | 0.17(0.08–0.27) | -38.68 | | 0.59(0.38–0.81) | -6.45 | | 2.33(2.01–2.66) | | -47.16 | | -0.01(-0.06–0.04) |  |
| Tajikistan | 9.64 | -0.5(-0.57–-0.43) | 22.01 | | 0(-0.06–0.05) | -19.5 | | -2.02(-2.3–-1.73) | | 21.79 | | -0.03(-0.09–0.03) |  |
| Thailand | -43.64 | 0.22(0.09–0.35) | -69.4 | | -2.35(-2.52–-2.18) | -10.3 | | 2.52(2.18–2.86) | | -86.32 | | -5.19(-6.32–-4.04) |  |
| Timor–Leste | -16.09 | -1.07(-1.17–-0.97) | -45.16 | | -2.8(-2.98–-2.62) | -4.6 | | -0.32(-0.39–-0.25) | | -3.03 | | -0.43(-0.46–-0.4) |  |
| Togo | 24.85 | -0.53(-0.58–-0.48) | 28.14 | | -0.55(-0.61–-0.49) | 28.9 | | -0.59(-0.69–-0.48) | | 40.81 | | -0.05(-0.07–-0.02) |  |
| Tokelau | -43.25 | -0.36(-0.45–-0.28) | -60.31 | | -1.83(-1.95–-1.71) | -28.64 | | 0.47(0.39–0.55) | | -39.09 | | -0.11(-0.19–-0.02) |  |
| Tonga | -14.63 | -0.23(-0.28–-0.19) | -20.76 | | -0.51(-0.58–-0.45) | -11.87 | | -0.1(-0.26–0.06) | | -7.5 | | 0.12(0.1–0.14) |  |
| Trinidad and Tobago | -31.68 | 0.12(0.11–0.12) | -45.44 | | -1.08(-1.24–-0.92) | -38.64 | | -0.12(-0.41–0.17) | | -31.91 | | 0.16(0.13–0.19) |  |
| Tunisia | -36.4 | -0.31(-0.41–-0.22) | -32.48 | | -0.37(-0.45–-0.29) | -5.56 | | 1.01(0.97–1.05) | | -31.89 | | -0.13(-0.15–-0.11) |  |
| Turkey | -42.12 | -0.18(-0.23–-0.13) | -37.47 | | -0.16(-0.38–0.06) | -14.04 | | 1.21(1.13–1.28) | | -41.44 | | -0.03(-0.09–0.02) |  |
| Turkmenistan | -14.15 | -0.06(-0.11–-0.02) | 4.1 | | 0.84(0.73–0.95) | 37.9 | | 1.75(1.52–1.98) | | -15.78 | | 0(-0.07–0.07) |  |
| Tuvalu | -29.16 | -0.2(-0.25–-0.14) | -63.76 | | -2.87(-3.05–-2.69) | -20.64 | | -0.12(-0.27–0.02) | | -25.94 | | -0.05(-0.12–0.02) |  |
| Uganda | 52.97 | -0.46(-0.62–-0.31) | 58.39 | | -0.09(-0.32–0.13) | 77.57 | | 0.33(0.16–0.49) | | 65.68 | | -0.14(-0.18–-0.1) |  |
| Ukraine | -36.83 | 0.3(0.25–0.35) | -50.07 | | -0.66(-0.85–-0.46) | -59.16 | | -1.08(-1.25–-0.92) | | -66.88 | | -2.71(-3.24–-2.18) |  |
| United Arab Emirates | 26.52 | 0.19(0.11–0.27) | 53.29 | | 0.71(0.63–0.8) | 51.62 | | 0.74(0.63–0.85) | | 17.89 | | -0.09(-0.18–0) |  |
| United Kingdom | -7.4 | -0.18(-0.28–-0.08) | -4.79 | | -0.16(-0.28–-0.04) | -5.97 | | -0.35(-0.42–-0.28) | | 20.13 | | 0.71(0.52–0.9) |  |
| United Republic of Tanzania | 22.83 | -0.98(-1.12–-0.84) | 72.09 | | -0.03(-0.18–0.12) | 93.73 | | 0.58(0.45–0.72) | | 64.89 | | -0.08(-0.11–-0.05) |  |
| United States of America | -0.28 | 0.28(0.09–0.46) | -12.65 | | -0.12(-0.21–-0.02) | -12.73 | | -0.16(-0.27–-0.04) | | -9.51 | | -0.05(-0.14–0.03) |  |
| United States Virgin Islands | -42.74 | 0.11(0.08–0.13) | -43.94 | | -0.09(-0.13–-0.05) | -41.96 | | 0.55(0.4–0.71) | | -43.87 | | 0.02(-0.01–0.04) |  |
| Uruguay | -11.49 | 0.26(0.21–0.31) | -25.59 | | -0.23(-0.35–-0.11) | -44.75 | | -1.44(-1.66–-1.21) | | -24.35 | | -0.45(-0.57–-0.33) |  |
| Uzbekistan | -3.16 | -0.19(-0.22–-0.16) | -9.52 | | -0.17(-0.32–-0.02) | 31.81 | | 0.9(0.73–1.07) | | -1.36 | | -0.01(-0.11–0.08) |  |
| Vanuatu | 26.93 | -0.2(-0.24–-0.16) | 13.36 | | -0.71(-0.75–-0.66) | 15.58 | | -0.75(-0.91–-0.58) | | 30.34 | | -0.16(-0.18–-0.15) |  |
| Venezuela | -10.57 | 0.35(0.26–0.44) | -22.11 | | 0.07(-0.13–0.27) | -8.12 | | 0.74(0.53–0.95) | | -14.4 | | 0.24(0.15–0.33) |  |
| Viet Nam | -44.65 | -1.22(-1.33–-1.12) | -52.97 | | -1.62(-1.76–-1.49) | -35.4 | | -0.42(-0.48–-0.36) | | -26.05 | | 0.06(0.04–0.08) |  |
| Yemen | 42.06 | 0.04(-0.01–0.09) | 25.58 | | -0.59(-0.67–-0.5) | 47.97 | | 0.4(0.33–0.48) | | 29.5 | | -0.29(-0.34–-0.25) |  |
| Zambia | 52.25 | -0.11(-0.22–0.01) | 46.86 | | -0.36(-0.43–-0.3) | 87.51 | | 0.72(0.6–0.85) | | 50.65 | | -0.22(-0.24–-0.2) |  |
| Zimbabwe | 6.57 | -0.36(-0.66–-0.06) | 26.21 | | 0.49(0.37–0.61) | 1.55 | | -0.6(-0.78–-0.42) | | 14.31 | | 0.01(-0.01–0.03) |  |

NPB, neonatal preterm birth; NE, neonatal encephalopathy due to birth asphyxia and trauma; NS, neonatal sepsis and other neonatal infections; HD, hemolytic disease and other neonatal jaundice; EAPC: estimated annual percentage change; CI, confidence interval.

**Supplementary table 4.** The percentage changes in number and the EAPCs of death caused by neonatal disorders in etiologies in global, SDI areas and geographic regions from 1990 to 2019

|  | **NPB** | | **NE** | | | **NS** | | | **HD** | |
| --- | --- | --- | --- | --- | --- | --- | --- | --- | --- | --- |
| **Characteristics** | Percentage (%) | EAPC  (95%CI) | | Percentage (%) | EAPC  (95%CI) | Percentage (%) | EAPC  (95%CI) | Percentage (%) | | EAPC  (95%CI) |
| **Overall** | -47.71 | -2.09(-2.19–-2.00) | | -29.89 | -1.16(-1.38–-0.94) | -12.93 | -0.53(-0.72–-0.35) | -58.83 | | -2.78(-3.00–-2.57) |
| **SDI** |  |  | |  |  |  |  |  | |  |
| Low | 4.62 | -1.32(-1.38–-1.27) | | 20.49 | -0.77(-0.88–-0.66) | 30.72 | -0.64(-0.72–-0.56) | -29.03 | | -2.48(-2.63–-2.32) |
| Low-middle | -50.05 | -1.94(-2.06–-1.83) | | -38.07 | -1.27(-1.53–-1.02) | -31.77 | -1.16(-1.31–-1.01) | -65.46 | | -3.09(-3.3–-2.89) |
| Middle | -66.85 | -3.02(-3.2–-2.84) | | -64.43 | -2.99(-3.32–-2.66) | -33.96 | -0.83(-1.11–-0.56) | -74.25 | | -3.73(-3.94–-3.51) |
| High-middle | -79.31 | -4.60(-4.74–-4.46) | | -77.91 | -4.53(-4.90–-4.15) | -48.81 | -1.59(-1.85–-1.33) | -80.96 | | -4.52(-4.77–-4.26) |
| High | -64.31 | -2.91(-3.00–-2.83) | | -61.96 | -2.82(-2.94–-2.70) | -56.38 | -2.77(-2.98–-2.55) | -82.37 | | -5.09(-5.4–-4.77) |
| **Regions** |  |  | |  |  |  |  |  | |  |
| East Asia | -85.94 | -5.32(-5.59–-5.06) | | -84.5 | -5.42(-6.30–-4.52) | -59.45 | -1.79(-1.96–-1.63) | -89.25 | | -6.23(-6.55–-5.9) |
| South Asia | -46.78 | -1.64(-1.77–-1.51) | | -33.79 | -0.98(-1.19–-0.76) | -39.17 | -1.67(-1.83–-1.52) | -58.29 | | -2.38(-2.57–-2.18) |
| Southeast Asia | -62.69 | -2.84(-2.92–-2.75) | | -59.93 | -2.60(-2.79–-2.41) | -40.81 | -1.21(-1.36–-1.07) | -76.46 | | -4.40(-4.54–-4.26) |
| Central Asia | -39.43 | -1.35(-1.72–-0.97) | | -52.26 | -2.60(-3.17–-2.03) | 9.62 | 0.92(0.66–1.18) | -76.99 | | -5.10(-5.72–-4.47) |
| High–income Asia Pacific | -82.52 | -4.27(-4.52–-4.03) | | -81.21 | -4.38(-4.60–-4.17) | -70.32 | -3.69(-4.00–-3.39) | -83.48 | | -3.91(-4.55–-3.26) |
| Oceania | 60.39 | -0.35(-0.44–-0.27) | | 53.24 | -0.56(-0.62–-0.49) | 88.84 | 0.17(-0.02–0.36) | 4.11 | | -1.91(-1.98–-1.84) |
| Australasia | -61.37 | -3.05(-3.42–-2.67) | | -34.13 | -1.81(-2.02–-1.60) | -58.23 | -3.91(-4.16–-3.66) | -82.18 | | -5.94(-6.91–-4.96) |
| Eastern Europe | -82.22 | -5.35(-5.69–-5.02) | | -84.78 | -6.15(-6.40–-5.90) | -40.69 | -1.37(-1.70–-1.04) | -89.18 | | -6.74(-7.04–-6.43) |
| Western Europe | -67.65 | -3.45(-3.59–-3.32) | | -63.2 | -2.94(-3.23–-2.65) | -50.61 | -2.01(-2.13–-1.89) | -80.94 | | -4.87(-5.32–-4.42) |
| Central Europe | -84.55 | -4.80(-5.04–-4.55) | | -88.61 | -6.36(-6.56–-6.15) | -78.02 | -4.58(-5.03–-4.13) | -91.25 | | -7.12(-7.37–-6.86) |
| High-income North America | -47.4 | -1.62(-1.73–-1.5) | | -43.25 | -1.49(-1.57–-1.41) | -27.35 | -0.81(-1.05–-0.57) | -70.19 | | -3.41(-3.86–-2.95) |
| Andean Latin America | -62.67 | -3.56(-3.76–-3.36) | | -61.26 | -3.32(-3.50–-3.15) | -25.9 | -1.24(-1.38–-1.1) | -78.83 | | -5.64(-5.87–-5.41) |
| Central Latin America | -70.78 | -3.82(-3.99–-3.65) | | -74.52 | -4.22(-4.34–-4.10) | -28.21 | -0.82(-1.03–-0.62) | -85.87 | | -5.98(-6.11–-5.85) |
| Caribbean | -36.68 | -1.08(-1.18–-0.99) | | -25.81 | -0.49(-0.61–-0.36) | 3.66 | 0.55(0.36–0.74) | -64.82 | | -3.1(-3.21–-2.99) |
| Tropical Latin America | -75.95 | -4.48(-4.59–-4.37) | | -60.86 | -2.63(-2.83–-2.42) | -53.03 | -2.3(-2.63–-1.97) | -85.38 | | -5.35(-5.64–-5.06) |
| Southern Latin America | -68.64 | -3.50(-3.62–-3.37) | | -72.78 | -4.36(-4.52–-4.20) | -58.87 | -3.21(-3.44–-2.99) | -86.85 | | -6.25(-6.51–-5.99) |
| Eastern Sub- Saharan Africa | -9.92 | -1.86(-1.95–-1.76) | | 12.11 | -0.95(-1.12–-0.78) | 25.11 | -0.64(-0.79–-0.49) | -27.22 | | -2.46(-2.62–-2.3) |
| Southern Sub- Saharan Africa | 9.00 | 0.62(0.22–1.01) | | -1.38 | 0.35(-0.11–0.81) | 9.68 | 0.55(0.25–0.85) | -40.74 | | -1.68(-2.04–-1.33) |
| Western Sub- Saharan Africa | 54.83 | -0.48(-0.55–-0.41) | | 40.04 | -0.86(-0.94–-0.77) | 49.76 | -0.72(-0.76–-0.68) | -15.73 | | -2.73(-2.82–-2.65) |
| North Africa and Middle East | -66.63 | -3.72(-3.82–-3.61) | | -65.88 | -3.77(-3.87–-3.67) | -33.2 | -1.66(-1.73–-1.60) | -74.93 | | -4.92(-5.04–-4.79) |
| Central Sub- Saharan Africa | 7.60 | -1.25(-1.42–-1.09) | | 14.75 | -1.03(-1.24–-0.82) | 38.54 | -0.44(-0.58–-0.29) | -19.49 | | -2.21(-2.46–-1.97) |

NPB, neonatal preterm birth; NE, neonatal encephalopathy due to birth asphyxia and trauma; NS, neonatal sepsis and other neonatal infections; HD, hemolytic disease and other neonatal jaundice; EAPC: estimated annual percentage change; CI, confidence interval; SDI: socio–demographic index.

**Supplementary table 5**. the percentage changes in number and the EAPCs of death caused by neonatal disorders in etiologies at national level from 1990 to 2019

|  | **NPB** | | | **NE** | | **NS** | | | **HD** | | |
| --- | --- | --- | --- | --- | --- | --- | --- | --- | --- | --- | --- |
| **Characteristics** | Percentage (%) | EAPC  (95%CI) | Percentage (%) | | EAPC  (95%CI) | Percentage  (%) | EAPC  (95%CI) | | Percentage (%) | EAPC  (95%CI) |  |
| Afghanistan | 39.88 | -2.4(-2.59–-2.21) | 49.75 | | -2.18(-2.37–-1.99) | 196.38 | 0.1(-0.02–0.22) | 42.64 | | -2.31(-2.64–-1.97) |  |
| Albania | -86.59 | -4.23(-4.6–-3.85) | -87.2 | | -4.16(-4.5–-3.82) | -78.73 | -2.79(-3.07–-2.52) | | -93.15 | -6.49(-6.82–-6.16) |  |
| Algeria | -53.79 | -2.96(-3.11–-2.82) | -56.48 | | -3.21(-3.34–-3.08) | -18.56 | -1.21(-1.27–-1.16) | | -72.13 | -4.82(-5.02–-4.62) |  |
| American Samoa | -62.77 | -1.79(-1.94–-1.64) | -69.84 | | -2.74(-3.01–-2.47) | -60.53 | -1.63(-2.33–-0.92) | | -78.29 | -3.92(-4.43–-3.4) |  |
| Andorra | -71.95 | -3.38(-3.86–-2.89) | -73.83 | | -3.7(-4.07–-3.32) | -50.78 | -2.2(-2.3–-2.11) | | -72.26 | -3.6(-4.02–-3.17) |  |
| Angola | 27.27 | -1.79(-1.91–-1.66) | 40.33 | | -1.44(-1.63–-1.25) | 65.96 | -0.86(-1.1–-0.61) | | -6.69 | -2.82(-3.03–-2.61) |  |
| Antigua and Barbuda | -59.71 | -2.81(-3.19–-2.42) | -65.73 | | -2.82(-3–-2.64) | -35.77 | -0.31(-0.51–-0.11) | | -72.8 | -4.04(-4.61–-3.48) |  |
| Argentina | -67.9 | -3.75(-3.9–-3.61) | -69.82 | | -4.44(-4.61–-4.27) | -58.3 | -3.53(-3.78–-3.28) | | -85.83 | -6.35(-6.62–-6.08) |  |
| Armenia | -89.97 | -6.07(-6.45–-5.68) | -91.26 | | -6.82(-7.45–-6.19) | -74.24 | -2.81(-3.22–-2.4) | | -98.46 | -13.08(-14.04–-12.11) |  |
| Australia | -63.33 | -3.43(-3.85–-3.01) | -25.1 | | -1.52(-1.8–-1.23) | -69.36 | -5.52(-5.84–-5.21) | | -82.7 | -6.21(-7.21–-5.2) |  |
| Austria | -68.4 | -4.18(-4.74–-3.63) | -74.01 | | -3.05(-4–-2.09) | -3.37 | 0.46(-0.67–1.61) | | -78.33 | -4.94(-5.6–-4.27) |  |
| Azerbaijan | -40.52 | -0.57(-1.18–0.04) | -42.88 | | -0.82(-1.43–-0.2) | -13.3 | 0.59(0.15–1.03) | | -52.1 | -1.13(-2.38–0.13) |  |
| Bahamas | -69.37 | -2.45(-2.8–-2.1) | -65.63 | | -2.6(-2.85–-2.36) | -38.67 | -0.65(-0.8–-0.5) | | -86.47 | -6.12(-6.3–-5.94) |  |
| Bahrain | -80.49 | -4.87(-5.55–-4.18) | -79.32 | | -4.95(-5.52–-4.39) | -34.77 | -0.24(-1.29–0.82) | | -74.27 | -3.72(-4.59–-2.85) |  |
| Bangladesh | -87.95 | -6.34(-6.56–-6.12) | -39.79 | | 0.89(0.12–1.67) | -54.18 | -1.07(-1.35–-0.8) | | -84.13 | -4.4(-4.76–-4.05) |  |
| Barbados | -60.88 | -1.9(-2.03–-1.77) | -60.15 | | -1.74(-1.8–-1.67) | -23.31 | 0.89(0.66–1.12) | | -80.86 | -4.44(-4.63–-4.25) |  |
| Belarus | -86.12 | -6.22(-6.65–-5.79) | -85.42 | | -5.98(-6.22–-5.74) | -53.35 | -1.62(-1.86–-1.37) | | -92.46 | -8.16(-8.48–-7.84) |  |
| Belgium | -75.67 | -4.52(-5.16–-3.87) | -53.61 | | -2.16(-2.52–-1.81) | -50.7 | -2.67(-3–-2.33) | | -84.54 | -6.94(-7.67–-6.21) |  |
| Belize | -48.75 | -3.32(-3.53–-3.1) | -35.9 | | -2.37(-2.48–-2.26) | 12.17 | -0.09(-0.36–0.18) | | -68.97 | -4.77(-5.03–-4.52) |  |
| Benin | 61.18 | -0.73(-0.8–-0.66) | 62.7 | | -0.78(-0.86–-0.7) | 46.81 | -1.18(-1.24–-1.13) | | -16.48 | -3.09(-3.16–-3.02) |  |
| Bermuda | -83.94 | -3.62(-4.41–-2.82) | -74.14 | | -2.32(-2.81–-1.82) | -60.04 | -0.88(-1.15–-0.61) | | -90.38 | -5.52(-6.19–-4.85) |  |
| Bhutan | -78.14 | -3.47(-3.57–-3.37) | -73.41 | | -2.89(-3.08–-2.7) | -62.57 | -1.88(-2.02–-1.73) | | -85.64 | -5(-5.16–-4.84) |  |
| Bolivia | -51.31 | -3.35(-3.5–-3.2) | -47.96 | | -3.12(-3.26–-2.98) | 18.74 | -0.09(-0.29–0.12) | | -74.88 | -5.66(-5.72–-5.61) |  |
| Bosnia and Herzegovina | -90.64 | -5.55(-6.08–-5.01) | -92.96 | | -6.61(-6.97–-6.25) | -60.51 | -0.08(-0.38–0.22) | | -94.77 | -7.92(-8.57–-7.26) |  |
| Botswana | 14.77 | 0.01(-0.25–0.28) | 11.13 | | -0.1(-0.39–0.19) | -7.75 | -0.67(-0.86–-0.48) | | -22.69 | -1.41(-1.7–-1.12) |  |
| Brazil | -76.11 | -4.49(-4.59–-4.4) | -60.62 | | -2.58(-2.79–-2.38) | -52.13 | -2.19(-2.52–-1.86) | | -85.44 | -5.35(-5.64–-5.07) |  |
| Brunei Darussalam | -15.39 | 0.3(0.03–0.56) | -21.01 | | 0.06(-0.21–0.34) | 7.1 | 0.99(0.78–1.2) | | -47.58 | -1.29(-1.63–-0.96) |  |
| Bulgaria | -60.93 | -1.33(-1.73–-0.94) | -54.57 | | -1.47(-1.93–-1.01) | 216.9 | 8.88(7.58–10.2) | | -90.12 | -7.71(-8.43–-6.99) |  |
| Burkina Faso | 76.38 | -0.09(-0.33–0.14) | 49.34 | | -1.04(-1.15–-0.92) | 60.17 | -1.03(-1.14–-0.91) | | -4.12 | -2.66(-2.81–-2.52) |  |
| Burundi | -10.78 | -2.38(-2.48–-2.27) | 30.69 | | -1.04(-1.25–-0.82) | 37.19 | -0.73(-0.89–-0.57) | | -19.45 | -2.72(-2.98–-2.47) |  |
| Cabo Verde | -51.91 | -1.9(-2.56–-1.25) | -46.46 | | -1.58(-2.12–-1.04) | -29.1 | -0.87(-1.22–-0.51) | | -69.81 | -3.88(-4.64–-3.11) |  |
| Cambodia | -55.35 | -2.04(-2.21–-1.87) | -53.32 | | -1.97(-2.19–-1.74) | -37.1 | -1.06(-1.24–-0.89) | | -75.26 | -4(-4.23–-3.77) |  |
| Cameroon | 39.91 | -1.08(-1.17–-0.99) | 36.19 | | -1.19(-1.25–-1.12) | 43.93 | -0.96(-1.06–-0.86) | | -28.25 | -3.32(-3.39–-3.26) |  |
| Canada | -42.35 | -1.05(-1.26–-0.84) | -29.62 | | -1.1(-1.29–-0.9) | -19.62 | -0.59(-0.77–-0.41) | | -70.07 | -3.56(-4–-3.11) |  |
| Central African  Republic | 38.81 | -0.07(-0.22–0.07) | 49.88 | | 0.16(0–0.32) | 69.81 | 0.48(0.33–0.63) | | 26.83 | -0.47(-0.68–-0.26) |  |
| Chad | 88.29 | -0.81(-0.93–-0.68) | 101.28 | | -0.6(-0.7–-0.51) | 76.42 | -1.3(-1.36–-1.23) | | 6.04 | -3.07(-3.2–-2.94) |  |
| Chile | -69.73 | -2.44(-2.73–-2.16) | -79.23 | | -3.86(-4.26–-3.46) | -64.3 | -2.4(-2.77–-2.03) | | -92.12 | -6.97(-7.44–-6.51) |  |
| China | -85.93 | -5.36(-5.62–-5.1) | -84.48 | | -5.48(-6.38–-4.57) | -59.99 | -1.91(-2.1–-1.72) | | -89.21 | -6.27(-6.6–-5.93) |  |
| Colombia | -74.18 | -4.23(-4.55–-3.92) | -82.9 | | -5.7(-5.83–-5.57) | 8.42 | 0.97(0.37–1.57) | | -92.28 | -8.23(-8.38–-8.07) |  |
| Comoros | -54.34 | -2.04(-2.22–-1.86) | -38.8 | | -1.06(-1.39–-0.72) | -41.86 | -1.2(-1.3–-1.11) | | -68.51 | -3.23(-3.49–-2.98) |  |
| Congo | 7.83 | -0.75(-0.97–-0.54) | 5.4 | | -0.82(-1.1–-0.54) | 28.7 | -0.14(-0.29–0.01) | | -25.53 | -1.98(-2.27–-1.69) |  |
| Cook Islands | -97.15 | -10.23(-10.98–-9.48) | -96.49 | | -9.6(-10.29–-8.9) | -81.73 | -3.97(-4.51–-3.43) | | -97.77 | -11.23(-11.68–-10.77) |  |
| Costa Rica | -60.6 | -2.31(-2.49–-2.13) | -59.69 | | -2.33(-2.48–-2.18) | -72.94 | -4.97(-5.76–-4.19) | | -62.34 | -1.81(-2.5–-1.11) |  |
| Croatia | -89.33 | -6.72(-7.16–-6.28) | -71.28 | | -1.72(-2.32–-1.11) | 166.57 | 3.19(0.58–5.86) | | -88.32 | -5.9(-6.42–-5.39) |  |
| Cuba | -85.73 | -4.53(-5.09–-3.97) | -86.28 | | -5.41(-5.78–-5.04) | -61.78 | -1.29(-1.61–-0.96) | | -91.6 | -6.12(-6.45–-5.8) |  |
| Cyprus | -81.31 | -6.22(-6.67–-5.77) | -81.29 | | -6.43(-6.84–-6.02) | -67.75 | -4.82(-5.41–-4.23) | | -72.03 | -4.58(-4.92–-4.25) |  |
| Czechia | -89.56 | -7.4(-8.67–-6.12) | -81.78 | | -4.91(-5.43–-4.38) | 71.4 | 3.05(2.16–3.95) | | -85.25 | -4.05(-5.32–-2.77) |  |
| Côte d'Ivoire | 37.5 | -0.27(-0.54–0) | 40.31 | | -0.17(-0.45–0.1) | 12.35 | -1.06(-1.15–-0.97) | | -36.62 | -3.09(-3.3–-2.88) |  |
| Democratic People's Republic of Korea | -87.43 | -4.27(-4.62–-3.92) | -85.37 | | -3.88(-4.34–-3.41) | -57.89 | -0.39(-0.55–-0.22) | | -90.95 | -5.3(-5.69–-4.91) |  |
| Democratic Republic of the Congo | -0.61 | -1.22(-1.41–-1.04) | 5.42 | | -1.02(-1.25–-0.79) | 29.52 | -0.39(-0.51–-0.28) | | -27.72 | -2.24(-2.52–-1.96) |  |
| Denmark | -36.06 | -0.55(-0.89–-0.2) | -59.06 | | -3.18(-3.31–-3.06) | -29.23 | -1.17(-1.76–-0.58) | | -66.61 | -2.81(-3.46–-2.15) |  |
| Djibouti | -0.29 | -1.65(-1.8–-1.5) | 32.38 | | -0.6(-0.88–-0.32) | 17.26 | -0.97(-1.15–-0.79) | | -20.34 | -2.42(-2.7–-2.14) |  |
| Dominica | -44.73 | 0.28(0.07–0.49) | -26.09 | | 2.05(1.64–2.46) | -35.79 | 1.47(1.21–1.73) | | -61.76 | -1.13(-1.31–-0.96) |  |
| Dominican Republic | -39.39 | -1.19(-1.37–-1) | -57.76 | | -2.39(-2.6–-2.17) | -0.08 | 0.44(0.17–0.72) | | -78.92 | -4.69(-5.06–-4.32) |  |
| Ecuador | -42.68 | -2.67(-3.22–-2.12) | -57.03 | | -4.03(-4.64–-3.41) | -45.67 | -3.68(-4.34–-3.02) | | -67.08 | -4.67(-5.4–-3.93) |  |
| Egypt | -85.75 | -7.4(-8.22–-6.56) | -83.05 | | -6.72(-7.42–-6.02) | -26.92 | -1.7(-2.03–-1.38) | | -87.09 | -7.6(-8.4–-6.79) |  |
| El Salvador | -83.35 | -3.99(-4.33–-3.64) | -87.01 | | -5.49(-5.66–-5.31) | -76.3 | -4.38(-4.8–-3.96) | | -88.04 | -5.42(-5.93–-4.91) |  |
| Equatorial Guinea | -5 | -1.98(-2.25–-1.71) | -10.65 | | -2.23(-2.57–-1.89) | 8.01 | -1.75(-1.96–-1.53) | | -53.66 | -4.56(-4.86–-4.25) |  |
| Eritrea | -9.14 | -1.45(-1.57–-1.32) | 28.08 | | -0.3(-0.47–-0.12) | 19.61 | -0.87(-0.97–-0.78) | | -27.57 | -2.16(-2.31–-2.02) |  |
| Estonia | -88.6 | -5.78(-6.39–-5.16) | -86.41 | | -5.68(-5.92–-5.44) | -88.56 | -7.29(-8.61–-5.95) | | -80.71 | -3.32(-3.72–-2.92) |  |
| Eswatini | -24.01 | -0.43(-0.6–-0.25) | -22.08 | | -0.45(-0.61–-0.28) | -19.98 | -0.38(-0.46–-0.3) | | -52.36 | -2.09(-2.29–-1.89) |  |
| Ethiopia | -38.52 | -3.18(-3.36–-3) | -9.88 | | -1.4(-1.64–-1.16) | 10.52 | -0.73(-0.98–-0.48) | | -44.48 | -3.08(-3.34–-2.82) |  |
| Fiji | -13.75 | -0.04(-0.18–0.1) | -38.15 | | -1.62(-1.9–-1.35) | -39.47 | -1.97(-2.4–-1.54) | | -56 | -2.77(-3.13–-2.41) |  |
| Finland | -83.07 | -5.03(-5.32–-4.74) | -66.48 | | -2.6(-2.8–-2.4) | -54.13 | -1.83(-2.2–-1.47) | | -75.29 | -3.23(-3.74–-2.72) |  |
| France | -67.1 | -3.21(-3.71–-2.71) | -49.23 | | -2.35(-2.54–-2.15) | -48.26 | -1.38(-1.89–-0.87) | | -81.98 | -6.1(-6.44–-5.76) |  |
| Gabon | -36.97 | -1.56(-1.87–-1.25) | -37.89 | | -1.55(-1.91–-1.19) | -13.63 | -0.43(-0.66–-0.2) | | -58.62 | -2.81(-3.21–-2.4) |  |
| Gambia | -15.06 | -1.66(-1.87–-1.44) | -9.08 | | -1.43(-1.63–-1.22) | -1.89 | -1.37(-1.45–-1.3) | | -56.72 | -4.19(-4.43–-3.95) |  |
| Georgia | -87.17 | -5.63(-6.23–-5.02) | -88.85 | | -6.82(-7.97–-5.65) | -6.41 | 2.63(1.6–3.67) | | -92.88 | -7.23(-8.31–-6.15) |  |
| Germany | -66.69 | -2.72(-2.93–-2.52) | -42.2 | | -0.54(-0.87–-0.2) | -59.16 | -2.93(-3.07–-2.79) | | -68.36 | -2.15(-2.58–-1.71) |  |
| Ghana | -10.31 | -2.27(-2.95–-1.58) | 1.69 | | -1.09(-1.25–-0.92) | 53.24 | 0.85(0.5–1.19) | | -40.2 | -2.85(-3.14–-2.55) |  |
| Greece | -74.29 | -4.2(-4.77–-3.64) | -74.3 | | -4.61(-5.43–-3.79) | -93 | -7.41(-9.32–-5.46) | | -78.21 | -5.37(-6.76–-3.95) |  |
| Greenland | -82.71 | -4.71(-4.94–-4.47) | -82.16 | | -4.77(-5.04–-4.49) | -52.66 | -1.49(-2.54–-0.43) | | -64.31 | -1.63(-1.91–-1.35) |  |
| Grenada | -60.93 | -1.85(-2.22–-1.49) | -56.57 | | -1.39(-1.61–-1.16) | -31.16 | 0.26(0.1–0.41) | | -78.87 | -4.18(-4.6–-3.77) |  |
| Guam | 28.63 | 1.93(1.47–2.39) | -21.66 | | -0.24(-0.41–-0.07) | -3.67 | 0.74(-0.05–1.54) | | -87.36 | -8.1(-8.9–-7.29) |  |
| Guatemala | -78.55 | -6.78(-7.26–-6.3) | -44.57 | | -2.28(-2.6–-1.95) | -32.17 | -2.31(-2.41–-2.2) | | -34.7 | -0.25(-1.28–0.78) |  |
| Guinea | -12.7 | -2.19(-2.31–-2.07) | -14.78 | | -2.31(-2.45–-2.17) | 11.8 | -1.19(-1.26–-1.12) | | -48.47 | -4.05(-4.22–-3.88) |  |
| Guinea-Bissau | -15.51 | -1.42(-1.58–-1.25) | -12.17 | | -1.31(-1.47–-1.16) | -10.97 | -1.35(-1.42–-1.28) | | -60.81 | -4.2(-4.32–-4.07) |  |
| Guyana | -73.76 | -2.85(-3.04–-2.66) | -70.41 | | -2.53(-2.69–-2.37) | -40.39 | -0.4(-0.8–0) | | -89.57 | -6.02(-6.29–-5.75) |  |
| Haiti | 26.32 | 0.02(-0.07–0.1) | 23.41 | | -0.13(-0.22–-0.04) | 49.69 | 0.41(0.28–0.55) | | -16.75 | -1.56(-1.68–-1.44) |  |
| Honduras | -47.5 | -3.02(-3.32–-2.72) | -46.48 | | -3.01(-3.22–-2.8) | -18.63 | -1.88(-2.02–-1.75) | | -68.59 | -4.91(-5.07–-4.75) |  |
| Hungary | -88.8 | -6.17(-6.43–-5.91) | -96.39 | | -9.44(-10.99–-7.87) | -56.13 | -0.34(-1.29–0.62) | | -85.7 | -2.44(-3.72–-1.14) |  |
| Iceland | -74.48 | -4.75(-5.16–-4.34) | -61.5 | | -3.4(-3.66–-3.14) | -63.36 | -3.16(-3.32–-2.99) | | -89.84 | -7.68(-7.92–-7.45) |  |
| India | -47.87 | -1.44(-1.63–-1.25) | -55.53 | | -2.31(-2.51–-2.12) | -43.98 | -1.98(-2.17–-1.8) | | -66.03 | -2.93(-3.18–-2.68) |  |
| Indonesia | -64.28 | -2.81(-2.93–-2.69) | -63.96 | | -2.75(-2.94–-2.56) | -52.17 | -1.77(-1.89–-1.65) | | -77.11 | -4.23(-4.35–-4.11) |  |
| Iran  (Islamic Republic of) | -86.48 | -5.14(-5.71–-4.56) | -85.47 | | -5.1(-5.59–-4.62) | -55.96 | -1.6(-1.87–-1.34) | | -95.33 | -9.29(-9.9–-8.68) |  |
| Iraq | -60.95 | -3.92(-4.34–-3.49) | -59.12 | | -3.73(-4.08–-3.37) | 1.46 | -0.67(-0.82–-0.51) | | -79.9 | -6.25(-6.58–-5.92) |  |
| Ireland | -62.65 | -3.93(-4.27–-3.6) | -55.51 | | -2.9(-3.29–-2.5) | 32.97 | 1.47(0.98–1.96) | | -64.58 | -2.99(-4.15–-1.81) |  |
| Israel | -73.07 | -6.6(-6.75–-6.44) | -33.28 | | -3.41(-3.99–-2.84) | -22.62 | -3.59(-3.98–-3.2) | | -62.82 | -4.72(-5.35–-4.07) |  |
| Italy | -83.8 | -5.63(-5.92–-5.35) | -82.64 | | -5.34(-5.74–-4.94) | -45.27 | -0.83(-1.17–-0.49) | | -87.58 | -5.82(-6.49–-5.14) |  |
| Jamaica | -54.68 | -1.51(-1.69–-1.33) | -51.89 | | -0.83(-0.96–-0.7) | -10.95 | 1.59(1.27–1.91) | | -92.4 | -9.07(-10.06–-8.07) |  |
| Japan | -82.94 | -4.81(-5.07–-4.54) | -79.02 | | -4.32(-4.59–-4.04) | -68.49 | -3.54(-3.76–-3.32) | | -78.21 | -2.87(-3.68–-2.05) |  |
| Jordan | -23.05 | -3.12(-3.34–-2.91) | -30.16 | | -3.34(-3.48–-3.19) | 18.53 | -1.51(-1.6–-1.41) | | -62.29 | -5.38(-5.64–-5.11) |  |
| Kazakhstan | -56.21 | -2.94(-3.77–-2.11) | -56.91 | | -2.87(-3.5–-2.24) | 1.23 | 0.15(-0.21–0.51) | | -80.62 | -5.52(-6.4–-4.64) |  |
| Kenya | -9.72 | -1.29(-1.44–-1.15) | 23.8 | | 0.11(-0.07–0.29) | 8.6 | -0.55(-0.62–-0.49) | | -30.72 | -2.11(-2.23–-2) |  |
| Kiribati | -33.54 | -1.89(-1.93–-1.85) | -34.69 | | -1.96(-2.07–-1.85) | 11.08 | -0.23(-0.5–0.04) | | -42.95 | -2.32(-2.44–-2.2) |  |
| Kuwait | -8.32 | -0.08(-0.63–0.48) | -61.63 | | -4.82(-5.09–-4.54) | -31.86 | -3.16(-3.57–-2.75) | | -86.63 | -8.67(-9.7–-7.62) |  |
| Kyrgyzstan | -37.34 | -0.93(-1.66–-0.18) | -44.53 | | -2.53(-3.03–-2.02) | 24.2 | 0.42(0.17–0.67) | | -89.82 | -8.24(-9.42–-7.04) |  |
| Lao People's Democratic Republic | -57.98 | -3.18(-3.38–-2.98) | -52.88 | | -2.8(-3.04–-2.56) | -18.63 | -0.84(-1.02–-0.65) | | -72.8 | -4.56(-4.78–-4.33) |  |
| Latvia | -88.91 | -6.21(-6.71–-5.71) | -82.04 | | -3.97(-4.55–-3.38) | -72.83 | -2.45(-3.07–-1.82) | | -91.57 | -6.74(-7.65–-5.82) |  |
| Lebanon | -78.62 | -5.08(-5.18–-4.98) | -78.99 | | -5.16(-5.25–-5.07) | -37.28 | -1.48(-1.58–-1.38) | | -84.3 | -5.9(-6.04–-5.76) |  |
| Lesotho | -40.25 | -0.82(-0.98–-0.66) | -40.07 | | -0.85(-1.05–-0.66) | -31.12 | -0.29(-0.43–-0.16) | | -57.64 | -2.09(-2.34–-1.84) |  |
| Liberia | -34.23 | -2.94(-3.14–-2.74) | -30.54 | | -2.81(-2.96–-2.66) | -15.07 | -2.33(-2.41–-2.26) | | -72.4 | -6.05(-6.29–-5.81) |  |
| Libya | -90.4 | -6.24(-6.42–-6.06) | -89.99 | | -6.09(-6.27–-5.92) | -70.23 | -2.11(-2.17–-2.05) | | -93.89 | -7.73(-7.94–-7.52) |  |
| Lithuania | -91.84 | -6.96(-7.44–-6.47) | -89.85 | | -6.41(-6.83–-5.98) | 29.96 | 5.71(4.29–7.14) | | -94.21 | -7.99(-8.98–-6.99) |  |
| Luxembourg | -76.49 | -5.94(-6.47–-5.4) | -69.96 | | -5.12(-5.7–-4.54) | -58.4 | -4.09(-4.6–-3.58) | | -88.07 | -8.22(-8.94–-7.5) |  |
| Madagascar | 0.05 | -1.36(-1.45–-1.28) | 23.9 | | -0.79(-0.93–-0.65) | 22.4 | -0.87(-0.96–-0.78) | | -27.71 | -2.5(-2.61–-2.39) |  |
| Malawi | -37.24 | -2.18(-2.27–-2.1) | -27.32 | | -1.53(-1.69–-1.38) | -13.7 | -1.06(-1.29–-0.82) | | -54.92 | -3.28(-3.46–-3.1) |  |
| Malaysia | -67.06 | -3.59(-4.33–-2.84) | -65.84 | | -3.59(-4.31–-2.87) | -45.01 | -2.56(-2.77–-2.36) | | -80.97 | -5.62(-6.01–-5.23) |  |
| Maldives | -76.73 | -4.87(-5.03–-4.72) | -66.85 | | -3.61(-3.91–-3.3) | -49.05 | -2.2(-2.38–-2.03) | | -81.32 | -5.65(-6–-5.29) |  |
| Mali | 48.07 | -1.22(-1.45–-1) | 39.17 | | -1.44(-1.59–-1.29) | 69.66 | -0.83(-0.95–-0.7) | | -4.5 | -2.89(-3.13–-2.65) |  |
| Malta | -65.93 | -2.78(-3.16–-2.39) | -56.88 | | -1.61(-1.93–-1.29) | -40.52 | -0.63(-0.91–-0.34) | | -80.24 | -4.62(-5.15–-4.09) |  |
| Marshall Islands | -49.16 | -1.72(-2.16–-1.28) | -53.93 | | -2.12(-2.54–-1.7) | -34.21 | -0.78(-1.04–-0.51) | | -76.06 | -4.41(-4.73–-4.09) |  |
| Mauritania | -30.38 | -1.69(-1.8–-1.57) | -27.72 | | -1.63(-1.75–-1.51) | -22.16 | -1.49(-1.62–-1.36) | | -59.78 | -3.59(-3.87–-3.31) |  |
| Mauritius | -77.77 | -3.77(-4.17–-3.37) | -77.35 | | -3.3(-3.57–-3.03) | -37.69 | 0.77(0.12–1.42) | | -93.26 | -6.87(-8.14–-5.58) |  |
| Mexico | -70.75 | -3.55(-3.73–-3.37) | -77.25 | | -4.4(-4.56–-4.24) | -27.3 | -0.68(-0.91–-0.44) | | -86.95 | -5.89(-6–-5.78) |  |
| Micronesia  (Federated States of) | -80.41 | -3.78(-3.91–-3.65) | -80.75 | | -3.88(-4.01–-3.74) | -60.97 | -1.3(-1.44–-1.16) | | -90.66 | -6.38(-6.58–-6.19) |  |
| Monaco | -67.96 | -4.33(-4.42–-4.24) | -63.75 | | -3.88(-3.96–-3.8) | -25.38 | -1.61(-1.64–-1.57) | | -68.72 | -4.37(-4.64–-4.1) |  |
| Mongolia | -59.95 | -3.44(-3.96–-2.91) | -63.13 | | -3.84(-4.39–-3.29) | -16.03 | -0.91(-1.15–-0.67) | | -74.01 | -5.03(-5.56–-4.49) |  |
| Montenegro | -89.52 | -6.69(-7.4–-5.98) | -86.5 | | -5.61(-6.27–-4.94) | -54.84 | -1.43(-1.67–-1.18) | | -89.29 | -6.7(-7.57–-5.83) |  |
| Morocco | -76.18 | -3.74(-4.06–-3.42) | -74.71 | | -3.56(-3.82–-3.31) | -43.23 | -0.85(-1.03–-0.67) | | -84.45 | -5.26(-5.7–-4.81) |  |
| Mozambique | 12.57 | -1.81(-1.95–-1.67) | 36.84 | | -1.02(-1.18–-0.86) | 51.62 | -0.79(-0.94–-0.64) | | -13.71 | -2.74(-2.92–-2.55) |  |
| Myanmar | -59.66 | -2.39(-2.61–-2.17) | -57.75 | | -2.09(-2.41–-1.78) | -47.28 | -1.47(-1.63–-1.31) | | -79.93 | -4.72(-4.88–-4.56) |  |
| Namibia | -13.57 | -0.96(-1.11–-0.81) | -12.84 | | -0.98(-1.14–-0.82) | 2.23 | -0.63(-0.71–-0.56) | | -48.53 | -2.8(-2.94–-2.66) |  |
| Nauru | -38.23 | 0.02(-0.65–0.69) | -47.59 | | -0.72(-1.35–-0.08) | -34.73 | -0.22(-0.59–0.15) | | -69 | -2.56(-3.18–-1.93) |  |
| Nepal | -79.39 | -4.96(-5.33–-4.59) | -75.89 | | -3.74(-4.2–-3.29) | -37.67 | -0.5(-0.69–-0.31) | | -76.2 | -3.48(-3.85–-3.11) |  |
| Netherlands | -63.7 | -3.17(-3.48–-2.86) | -59.31 | | -2.75(-2.9–-2.6) | -13.15 | 0.34(0.13–0.55) | | -89.44 | -6.59(-7.42–-5.76) |  |
| New Zealand | -53.41 | -1.6(-2.07–-1.12) | -70.66 | | -4(-4.22–-3.77) | 50.11 | 2.89(2.19–3.6) | | -78.81 | -4.48(-5.38–-3.57) |  |
| Nicaragua | -78.58 | -4.75(-4.84–-4.65) | -79.77 | | -5.24(-5.36–-5.12) | -56.44 | -2.78(-3.14–-2.42) | | -92.96 | -9.7(-10.26–-9.13) |  |
| Niger | 105.52 | -1.21(-1.42–-1.01) | 96.38 | | -1.33(-1.53–-1.13) | 107.07 | -1(-1.11–-0.89) | | 15.91 | -3.34(-3.6–-3.08) |  |
| Nigeria | 78.15 | 0.12(0.02–0.22) | 52.4 | | -0.5(-0.62–-0.38) | 59.67 | -0.56(-0.66–-0.46) | | 0.46 | -2.09(-2.23–-1.94) |  |
| Niue | -64.59 | -1.16(-1.45–-0.88) | -67.85 | | -1.57(-1.86–-1.28) | -62.76 | -0.85(-1.05–-0.66) | | -82.28 | -3.77(-3.98–-3.55) |  |
| North Macedonia | -77.34 | -2.55(-3.07–-2.04) | -82.68 | | -4(-4.47–-3.54) | 227.49 | 9.31(7.81–10.83) | | -73.31 | -3.06(-3.69–-2.43) |  |
| Northern Mariana Islands | -63.27 | 0.8(0.06–1.55) | -75.18 | | -0.88(-1.34–-0.41) | -57.61 | 0.93(0.34–1.53) | | -93.62 | -6.82(-7.82–-5.82) |  |
| Norway | -76.35 | -4.02(-4.34–-3.69) | -72.72 | | -4.1(-4.38–-3.81) | -43.76 | -1.59(-2.08–-1.1) | | -75.15 | -3.37(-3.9–-2.85) |  |
| Oman | -69.2 | -4.15(-5.22–-3.06) | -71.49 | | -4.53(-5.41–-3.64) | -26.41 | -1.46(-1.68–-1.24) | | -78.7 | -5.47(-6.51–-4.43) |  |
| Pakistan | 9.79 | -0.61(-0.69–-0.52) | 16.64 | | -0.41(-0.6–-0.23) | 9.7 | -0.83(-0.96–-0.7) | | -15.83 | -1.4(-1.64–-1.16) |  |
| Palau | -79.47 | -2.68(-2.87–-2.5) | -77.83 | | -2.57(-2.73–-2.42) | -66.41 | -1.48(-1.59–-1.37) | | -89.2 | -5.01(-5.35–-4.66) |  |
| Palestine | -62.65 | -3.8(-4.28–-3.33) | -62.87 | | -3.83(-4.12–-3.54) | -4.8 | -0.72(-0.98–-0.46) | | -78.72 | -5.02(-5.6–-4.44) |  |
| Panama | -40.6 | -2.87(-3.16–-2.59) | -39.03 | | -2.21(-2.38–-2.05) | -4 | -0.85(-1.05–-0.64) | | -86.86 | -9.1(-10.07–-8.12) |  |
| Papua New Guinea | 90.45 | -0.35(-0.45–-0.26) | 92.33 | | -0.32(-0.4–-0.25) | 137.19 | 0.41(0.22–0.61) | | 15.23 | -2.12(-2.2–-2.04) |  |
| Paraguay | -66.38 | -3.61(-4.11–-3.1) | -71.64 | | -4.52(-5.01–-4.03) | -78.13 | -6.26(-6.98–-5.53) | | -79.3 | -4.98(-5.77–-4.17) |  |
| Peru | -76.28 | -4.53(-4.7–-4.35) | -68.59 | | -3.3(-3.53–-3.07) | -37.16 | -1.19(-1.33–-1.05) | | -86.6 | -6.85(-7.05–-6.65) |  |
| Philippines | -24.86 | -1.53(-1.66–-1.39) | -30.72 | | -1.85(-2.01–-1.69) | -8.54 | -0.94(-1.24–-0.64) | | -55.03 | -3.52(-3.88–-3.15) |  |
| Poland | -86.82 | -5.34(-5.88–-4.8) | -90.75 | | -7.62(-7.93–-7.32) | -87.11 | -7.03(-7.74–-6.32) | | -94.85 | -8.55(-9.03–-8.08) |  |
| Portugal | -91.91 | -7.17(-7.58–-6.76) | -86.8 | | -4.47(-5.3–-3.64) | -77.92 | -3.57(-3.97–-3.17) | | -93.56 | -7.18(-7.97–-6.39) |  |
| Puerto Rico | -87.13 | -3.92(-4.28–-3.56) | -83.44 | | -3.01(-3.14–-2.88) | -54.87 | 1.03(0.35–1.73) | | -90.6 | -5.13(-5.7–-4.55) |  |
| Qatar | -34.54 | -5.06(-5.21–-4.91) | -35.52 | | -5.28(-5.5–-5.06) | 24.66 | -3.14(-3.38–-2.9) | | 1.66 | -2.56(-3.08–-2.04) |  |
| Republic of Korea | -82.74 | -3.48(-3.98–-2.97) | -84.27 | | -4.27(-4.61–-3.93) | -72.77 | -3.45(-3.87–-3.02) | | -88.9 | -4.92(-5.39–-4.44) |  |
| Republic of Moldova | -76.38 | -1.44(-2.17–-0.71) | -90.29 | | -5.75(-6.2–-5.29) | -68.51 | -1.09(-1.58–-0.59) | | -89.01 | -4.24(-4.81–-3.67) |  |
| Romania | -63.43 | -1.45(-1.75–-1.15) | -86.03 | | -6.08(-6.64–-5.51) | -68.81 | -2.77(-3.35–-2.18) | | -87.9 | -6.09(-6.77–-5.41) |  |
| Russian Federation | -81.82 | -5.67(-6.11–-5.23) | -86.56 | | -7.1(-7.4–-6.8) | -33.78 | -1.59(-2.07–-1.11) | | -88.1 | -6.79(-7.14–-6.43) |  |
| Rwanda | -53.14 | -2.85(-3.17–-2.53) | -29.99 | | -1.47(-1.76–-1.18) | -21.13 | -0.99(-1.21–-0.77) | | -59.46 | -3.3(-3.65–-2.95) |  |
| Saint Kitts and Nevis | -67.36 | -2.9(-3.28–-2.52) | -65.34 | | -2.74(-2.96–-2.53) | -25.06 | 0.17(0.04–0.3) | | -81.77 | -5.25(-5.61–-4.89) |  |
| Saint Lucia | -69.41 | -1.21(-1.36–-1.06) | -70.75 | | -1.34(-1.55–-1.13) | -52.26 | 0.56(0.28–0.84) | | -84.27 | -3.79(-4.22–-3.36) |  |
| Saint Vincent and the Grenadines | -74.94 | -2.72(-2.92–-2.52) | -79.26 | | -3.23(-3.32–-3.14) | -24.37 | 0.32(-0.22–0.87) | | -93.39 | -6.79(-7.06–-6.52) |  |
| Samoa | -64.36 | -3.64(-3.78–-3.5) | -63.39 | | -3.52(-3.6–-3.45) | -24.72 | -1.23(-1.35–-1.1) | | -79.76 | -5.62(-5.82–-5.41) |  |
| San Marino | -57.94 | -3.59(-3.83–-3.35) | -54.02 | | -3.26(-3.49–-3.03) | -33.49 | -2.19(-2.35–-2.03) | | -71.96 | -5.03(-5.44–-4.62) |  |
| Sao Tome and Principe | -34.61 | -1.21(-1.43–-0.98) | -34.45 | | -1.22(-1.47–-0.96) | -33.66 | -1.46(-1.58–-1.34) | | -69.71 | -4.02(-4.29–-3.76) |  |
| Saudi Arabia | -91.86 | -7.87(-8.28–-7.45) | -92.5 | | -8.79(-9.23–-8.35) | -77.66 | -5.3(-5.7–-4.9) | | -93.25 | -8.06(-8.46–-7.66) |  |
| Senegal | 3.12 | -0.91(-1.04–-0.78) | 5.36 | | -0.85(-0.99–-0.71) | -10.19 | -1.26(-1.33–-1.18) | | -52.81 | -3.56(-3.69–-3.42) |  |
| Serbia | -90.82 | -6.34(-6.93–-5.73) | -93.21 | | -7.93(-8.59–-7.28) | -90.23 | -8.66(-9.83–-7.47) | | -88.52 | -5.41(-6.04–-4.78) |  |
| Seychelles | -60.76 | -1.97(-2.28–-1.66) | -60.15 | | -2.03(-2.29–-1.78) | -36.01 | -0.76(-0.9–-0.62) | | -67.83 | -2.46(-2.87–-2.06) |  |
| Sierra Leone | -7.14 | -2.31(-2.38–-2.24) | -10.95 | | -2.48(-2.55–-2.41) | 21.94 | -1.43(-1.49–-1.37) | | -49.99 | -4.49(-4.59–-4.38) |  |
| Singapore | -83.33 | -5.8(-6.22–-5.38) | -84.26 | | -6.1(-6.77–-5.43) | -1.38 | 0.41(-0.38–1.21) | | -43.64 | -1.35(-1.79–-0.91) |  |
| Slovakia | -76.66 | -3.66(-3.92–-3.39) | -67.3 | | -2.48(-2.65–-2.31) | -40.17 | -0.95(-1.32–-0.59) | | -81.26 | -4.43(-4.65–-4.21) |  |
| Slovenia | -86.81 | -5.84(-6.14–-5.53) | -87.84 | | -7.05(-7.34–-6.77) | -7.4 | 2.43(1.51–3.36) | | -70.43 | -2.9(-3.2–-2.59) |  |
| Solomon Islands | -22.67 | -2.22(-2.43–-2.02) | -26.24 | | -2.51(-2.74–-2.28) | 8.24 | -1.19(-1.51–-0.88) | | -60.87 | -4.74(-4.85–-4.62) |  |
| Somalia | 70.02 | -1.24(-1.38–-1.1) | 139.53 | | -0.09(-0.25–0.08) | 146.57 | -0.21(-0.32–-0.11) | | 64.24 | -1.3(-1.45–-1.15) |  |
| South Africa | 7.83 | 0.95(0.37–1.53) | -4.08 | | 0.58(0–1.16) | 14.66 | 0.93(0.49–1.36) | | -45.15 | -1.77(-2.21–-1.34) |  |
| South Sudan | -8.02 | -1.26(-1.41–-1.11) | 33.18 | | 0.01(-0.13–0.15) | 17.15 | -0.69(-0.75–-0.63) | | -10.96 | -1.29(-1.44–-1.14) |  |
| Spain | -81.99 | -5.23(-5.61–-4.84) | -68.51 | | -3.21(-3.71–-2.71) | -42.17 | -1.52(-1.8–-1.24) | | -82.83 | -4.72(-5.33–-4.11) |  |
| Sri Lanka | -69.82 | -2.75(-3.23–-2.28) | -81.14 | | -5.15(-5.5–-4.8) | -35.58 | -0.42(-0.62–-0.22) | | -49.06 | 0.06(-0.91–1.05) |  |
| Sudan | -46.86 | -2.41(-2.56–-2.26) | -49.92 | | -2.75(-2.87–-2.64) | -8.8 | -0.83(-0.94–-0.72) | | -63.31 | -3.71(-3.85–-3.57) |  |
| Suriname | -50.16 | -2.51(-2.64–-2.37) | -44 | | -2.03(-2.28–-1.79) | -9.36 | -0.01(-0.2–0.18) | | -68.59 | -4.32(-4.72–-3.91) |  |
| Sweden | -69.68 | -3.49(-4.07–-2.91) | -59.75 | | -2.38(-2.86–-1.89) | -37.88 | -2.3(-2.7–-1.89) | | -77.84 | -3.49(-4.32–-2.65) |  |
| Switzerland | -50.16 | -2.18(-2.41–-1.96) | 2.02 | | 0.59(0.24–0.94) | -35.25 | -2.25(-2.65–-1.85) | | -48.58 | -2.02(-2.3–-1.75) |  |
| Syrian Arab Republic | -91.97 | -5.41(-6.79–-4.02) | -90.86 | | -4.98(-6.11–-3.83) | -76.05 | -2.07(-2.42–-1.73) | | -94.83 | -6.72(-8.01–-5.41) |  |
| Taiwan  (Province of China) | -53.46 | -2.25(-3.57–-0.91) | -56.36 | | -0.19(-0.39–0.01) | 178.93 | 8.48(7.45–9.52) | | -93.5 | -7.98(-8.84–-7.11) |  |
| Tajikistan | -8.6 | -1.19(-1.47–-0.9) | -13.3 | | -1.34(-1.63–-1.05) | 28.53 | 0.15(-0.05–0.35) | | -47.39 | -3.18(-3.54–-2.82) |  |
| Thailand | -90.36 | -6.25(-6.56–-5.94) | -88.59 | | -5.47(-5.73–-5.21) | -35.83 | 1.85(1.23–2.47) | | -74.23 | -1.25(-1.91–-0.59) |  |
| Timor-Leste | -45.4 | -2.34(-2.44–-2.23) | -43.12 | | -2.26(-2.41–-2.11) | -19.15 | -1.2(-1.35–-1.06) | | -63.86 | -3.93(-4.09–-3.77) |  |
| Togo | -8.72 | -1.49(-1.53–-1.46) | -6.14 | | -1.39(-1.43–-1.34) | -5.34 | -1.56(-1.63–-1.49) | | -50.61 | -3.74(-3.83–-3.65) |  |
| Tokelau | -86.44 | -5.3(-5.57–-5.04) | -86.7 | | -5.39(-5.69–-5.09) | -65.17 | -2.1(-2.36–-1.85) | | -93.46 | -7.82(-7.97–-7.66) |  |
| Tonga | -57.11 | -2.47(-2.62–-2.32) | -57.94 | | -2.54(-2.74–-2.34) | -31.55 | -0.88(-1–-0.76) | | -78.44 | -4.97(-5.08–-4.87) |  |
| Trinidad and Tobago | -68.09 | -3.1(-3.54–-2.66) | -76.1 | | -4.53(-5.04–-4.02) | -50.3 | -1.57(-2.16–-0.97) | | -83.18 | -5.22(-6.23–-4.21) |  |
| Tunisia | -86.1 | -5.39(-5.54–-5.23) | -85.24 | | -5.21(-5.32–-5.09) | -58.9 | -1.81(-1.86–-1.76) | | -89.93 | -6.53(-6.67–-6.39) |  |
| Turkey | -87.51 | -5.2(-5.33–-5.07) | -83.85 | | -4.16(-4.28–-4.03) | -67.06 | -2.03(-2.12–-1.94) | | -92.02 | -6.38(-6.54–-6.22) |  |
| Turkmenistan | -23.57 | 0.27(-0.31–0.85) | -54.87 | | -2.28(-2.58–-1.97) | 33.77 | 2.12(1.75–2.5) | | -65.24 | -2.65(-3.12–-2.18) |  |
| Tuvalu | -84.49 | -5.39(-5.47–-5.31) | -84.09 | | -5.38(-5.5–-5.27) | -52.55 | -1.6(-1.74–-1.47) | | -92.6 | -7.92(-8.07–-7.77) |  |
| Uganda | 30.36 | -0.58(-0.75–-0.41) | 27.67 | | -0.63(-0.84–-0.43) | 29.55 | -0.59(-0.77–-0.42) | | -9.29 | -1.77(-2–-1.53) |  |
| Ukraine | -82.44 | -4.59(-4.98–-4.19) | -70.85 | | -2.54(-2.77–-2.32) | -45.6 | -0.31(-0.47–-0.15) | | -91.95 | -6.98(-7.53–-6.43) |  |
| United Arab Emirates | -82.57 | -5.65(-6.11–-5.2) | -81.2 | | -5.83(-6.2–-5.47) | -39.75 | -2.07(-2.24–-1.9) | | -85.1 | -6.31(-6.75–-5.87) |  |
| United Kingdom | -43.57 | -1.79(-2–-1.58) | -70.14 | | -3.81(-4.12–-3.49) | -62.59 | -4.08(-4.45–-3.71) | | -82.35 | -4.99(-5.66–-4.32) |  |
| United Republic of Tanzania | 25.25 | -0.68(-0.95–-0.42) | 40.24 | | -0.52(-0.66–-0.37) | 44.38 | -0.39(-0.53–-0.25) | | -9.01 | -1.92(-2.06–-1.77) |  |
| United States of America | -47.67 | -1.65(-1.76–-1.53) | -44.58 | | -1.54(-1.65–-1.44) | -27.76 | -0.81(-1.05–-0.58) | | -70.2 | -3.39(-3.86–-2.92) |  |
| United States Virgin Islands | -82.92 | -3.91(-4.07–-3.76) | -80.03 | | -3.41(-3.56–-3.26) | -51.97 | -0.28(-0.44–-0.13) | | -80.56 | -3.31(-3.43–-3.18) |  |
| Uruguay | -78.53 | -4.98(-5.27–-4.69) | -82.04 | | -5.55(-5.74–-5.35) | -55.45 | -2.23(-2.55–-1.91) | | -88.82 | -5.85(-6.89–-4.81) |  |
| Uzbekistan | -4.59 | 0.08(-0.14–0.31) | -56.09 | | -2.91(-3.58–-2.23) | 94.31 | 3.4(2.93–3.88) | | -72.53 | -5.2(-6.03–-4.37) |  |
| Vanuatu | -3.94 | -0.89(-1.12–-0.66) | -11.59 | | -1.27(-1.5–-1.04) | 13.09 | -0.56(-0.78–-0.33) | | -51.18 | -3.34(-3.44–-3.24) |  |
| Venezuela | -58.99 | -2.06(-2.41–-1.72) | -53.28 | | -1.91(-2.1–-1.73) | -33.9 | -0.8(-1.06–-0.54) | | -80.56 | -4.67(-5.02–-4.31) |  |
| Viet Nam | -79.99 | -4.31(-4.45–-4.17) | -78.54 | | -3.95(-4.14–-3.75) | -60.35 | -2.16(-2.22–-2.1) | | -85.62 | -5.46(-5.64–-5.27) |  |
| Yemen | -22.41 | -1.91(-1.97–-1.86) | -26.56 | | -2.14(-2.19–-2.1) | 25.23 | -0.14(-0.24–-0.04) | | -40.66 | -2.89(-3.02–-2.75) |  |
| Zambia | 12.86 | -1.04(-1.3–-0.78) | 18.96 | | -0.79(-1.07–-0.51) | 25.91 | -0.75(-0.91–-0.6) | | -29.8 | -2.58(-2.92–-2.24) |  |
| Zimbabwe | 27.54 | 0.58(0.4–0.75) | 23.68 | | 0.67(0.34–1.01) | 13.72 | 0.31(0.14–0.47) | | -1.38 | -0.16(-0.43–0.12) |  |

NPB, neonatal preterm birth; NE, neonatal encephalopathy due to birth asphyxia and trauma; NS, neonatal sepsis and other neonatal infections; HD, hemolytic disease and other neonatal jaundice; EAPC: estimated annual percentage change; CI, confidence interval.
